# Dynamic spatiotemporal activation of a pervasive neurogenic competence in striatal astrocytes supports continuous neurogenesis following injury

**DOI:** 10.1016/j.stemcr.2024.08.006

**Published:** 2024-09-19

**Authors:** Marco Fogli, Giulia Nato, Philip Greulich, Jacopo Pinto, Marta Ribodino, Gregorio Valsania, Paolo Peretto, Annalisa Buffo, Federico Luzzati

**Affiliations:** 1 Neuroscience Institute Cavalieri Ottolenghi, Orbassano (Turin), Italy; 2 Department of Life Sciences and System Biology, University of Turin, Turin, Italy; 3 Department of Neurosciences “Rita Levi Montalcini”, University of Turin, Turin, Italy; 4 School of Mathematical Sciences, University of Southampton, Southampton, UK; 5 Institute for Life Sciences (IfLS), University of Southampton, Southampton, UK

**Keywords:** neural stem cells, astrocytes, parenchymal neurogenesis, neurogenic potential, stem cell quiescence, lesion induced neurogenesis, astrocyte reactivity, stem cell dynamics, clonal analysis, spatial statistics

## Abstract

Adult neural stem cells (NSCs) are conventionally regarded as rare cells restricted to two niches: the subventricular zone (SVZ) and the subgranular zone. Parenchymal astrocytes (ASs) can also contribute to neurogenesis after injury; however, the prevalence, distribution, and behavior of these latent NSCs remained elusive. To tackle these issues, we reconstructed the spatiotemporal pattern of striatal (STR) AS neurogenic activation after excitotoxic lesion in mice. Our results indicate that neurogenic potential is widespread among STR ASs but is focally activated at the lesion border, where it associates with different reactive AS subtypes. In this region, similarly to canonical niches, steady-state neurogenesis is ensured by the continuous stochastic activation of local ASs. Activated ASs quickly return to quiescence, while their progeny transiently expand following a stochastic behavior that features an acceleration in differentiation propensity. Notably, STR AS activation rate matches that of SVZ ASs indicating a comparable prevalence of NSC potential.

## Highlights

• A widespread dormant neurogenic potential is present among striatal astrocytes
• Striatal neurogenesis is supported by the stochastic activation of local astrocytes
• NSC prevalence and dynamics are similar in striatal and neurogenic niche astrocytes
• Activated striatal astrocytes are maintained with high probability

## Teaser

The spatiotemporal dynamics of striatal astrocyte neurogenic activation after a lesion unveil an unprecedented level of neurogenic competence within the mature brain parenchyma, comparable to that found in canonical niches.

## Introduction

Stem cells are typically rare cells confined to specialized anatomical regions, called niches, that ensure their maintenance and regulate their activity (Altshuler et al., 2023). In the adult mammalian brain, two such regions have been identified, the subventricular zone (SVZ) and the subgranular zone (SGZ), where astrocyte (AS)-like cells act as neural stem cells (NSCs) throughout life (Bond et al., 2015; Dray et al., 2021a; Obernier and Alvarez-Buylla, 2019). These cells are mostly quiescent (qNSC) but sporadically activate generating transit amplifying progenitors (TAPs) that further divide before differentiating into neuroblasts (NBs) (Pilz et al., 2018; Ponti et al., 2013). The stochastic activation of widespread qNSCs within the niche ensures continuous neuron production (Basak et al., 2018; Than-Trong et al., 2020). Outside these niches, the mature brain parenchyma has been traditionally considered non-permissive for neurogenesis (Götz et al., 2015). However, single-cell RNA sequencing (RNA-seq) revealed striking similarities between parenchymal ASs and qNSC (Llorens-Bobadilla et al., 2015). and all major elements maintaining stem cells in canonical niches are present in the parenchyma (Boda et al., 2017). Parenchymal ASs may thus represent NSCs in a deep quiescent state. According to this notion, during early postnatal development (Laywell et al., 2000) or after injuries inducing proliferative AS reactivity, subsets of parenchymal ASs can expand *in vitro* as neurospheres (Buffo et al., 2008; Sirko et al., 2013) and become transcriptionally similar to NSC primed for activation (Zamboni et al., 2020). Notably, in the mouse striatum (STR), some ASs move beyond the primed state and express their neurogenic capacity *in vivo*, after stroke or quinolinic acid (QA)-mediated excitotoxic lesion, supporting neurogenesis for several months (Magnusson et al., 2014; Nato et al., 2015; Thored et al., 2006). However, the prevalence, spatial distribution, and dynamics of these ectopic NSCs were not resolved. Consequently, how widespread is NSC potential among parenchymal ASs and to what extent the parenchyma is permissive for its maintenance and expression remain unclear. STR neurogenic ASs may simply represent a new rare NSC population. Accordingly, the mainstream view in the field still adheres to the concept of the anatomical restriction of NSCs potential (Urbán et al., 2019). To address these issues, we investigated the spatiotemporal dynamics of neurogenic activity and lineage progression of STR ASs after QA lesion.

To reach this goal, we built upon our previous demonstration that in this model new STR neurons originate exclusively from STR ASs through neurogenic foci scattered around the lesion border. These foci are made of cells expressing the proliferation marker Ki67 and organized in clusters (KCs). The KCs include a few putative ASs but mostly TAPs-like cells and proliferating NBs (prNBs) and are often associated with their early postmitotic NBs (pmNBs) (Nato et al., 2015).

Now we demonstrate that these neurogenic foci are transient structures continuously generated by the stochastic activation of a widespread population of neurogenic ASs residing in a globally permissive environment. By clarifying the spatiotemporal dynamics of these atypical progenitors, we unveiled an unprecedented level of neurogenic competence among parenchymal ASs, comparable to that of canonical niches ASs.

## Results

### Neurogenic foci organize in a 3D germinal matrix centered around the lesion border

**Figure 1:**
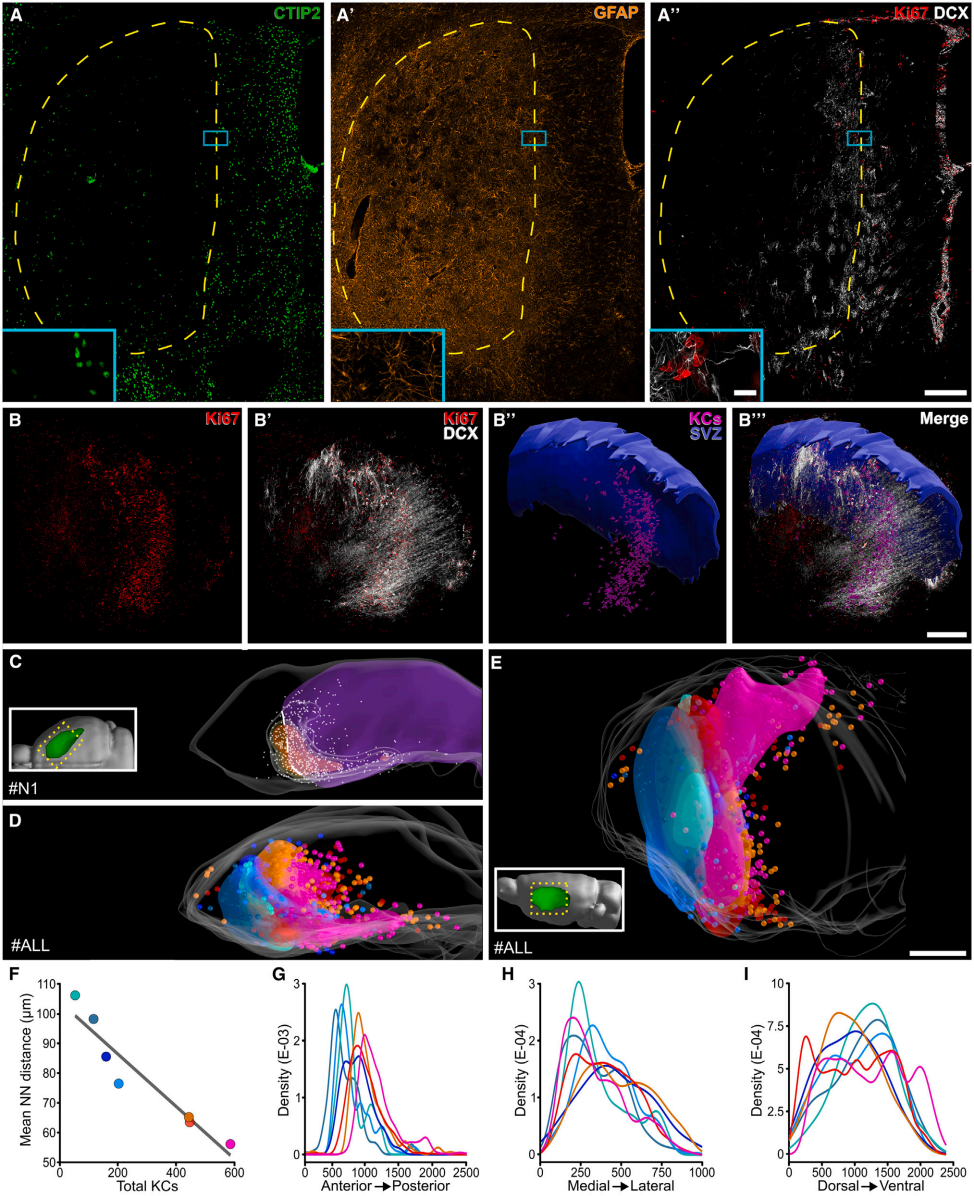
Neurogenic foci form a 3D germinal matrix (A–A″) CTIP2-, GFAP-, Ki67-, and DCX-stained section. Dashed yellow line: lesioned area. Inset: high magnification of a KC. (B–B‴) 3D reconstruction of STR Ki67 and DCX staining (specimen #N1; see also Video S1). SVZ is hidden. In (B″-B‴) segmented STR KCs are in magenta. (C) Dorsal view of #N1. The lesion is in purple. White dashed line: the lesion border in the neurogenic area. Gray dots: KC (25 μm diameter). Increasing relative KC density is rendered as transparent, yellow, and orange volumes. (D and E) Overlap of 7 specimens in a common coordinate frame. The KC (100 μm radius) and the volume rendering of KC density are colored by specimen. (F) Correlation between the number of KC and mean nearest neighbor distance among them. Black line: linear regression. (G–I) Relative KC distribution along the antero-posterior (G), medio-lateral (H), and dorsoventral (I) axis. Scale: (A) 200 μm; (inset A) 20 μm; (B–B‴), (C), (D), and (E) 500 μm.

To analyze the spatial distribution of the KCs, defined as groups of at least 4 cells in direct contact, we 3D reconstructed the STR of 7 specimens at 5 weeks post lesion (wpl). As previously described (Nato et al., 2015), QA lesions caused the loss of STR neurons in a large dorsolateral domain that was filled by densely arranged GFAP^+^ ASs (Figures 1A–1A′). KCs organized in a 3D germinal matrix centered around the rostro-medial part of the lesion border in both lesioned and spared tissue (Figures 1A″–1C, S1A, and S1B; Video S1).

Video S1. 3D reconstruction of seven striata at 5 wpl, related to Figures 1 and S1 The 528 focal planes of the 40 sequential sections 3D reconstructed from specimen #N1 ^∗^voxel size 0.76 × 0.76 × 3.78 μm are shown from the most anterior to the posterior and then are presented all together as an MAX projection. Note that the DCX^+^ pmNBs generated by KC further extended rostrally and caudally, partly inside white matter tracts. After a 360° rotation of the whole reconstruction, a couple of isolated KCs are shown. Ki67 staining is presented alone, and the KC segmentation (magenta) is overlaid. Note the sparse Ki67^+^ cells that are excluded from the segmentation as they may represent new AS activation events or other proliferating cell types. Clustered Ki67^+^ cells in the SVZ are shown in blue. By min 1:14 blender renderings of surfaces and manually annotated KC, represented as balls, are shown. A measure of relative KC density is shown as an object of different colors, with the hottest representing the highest value. The KCs distribute along the lesion (purple) border, mainly at its rostro-medial border, the ventricle is in blue, and the STR is transparent. From 1:25 onward each of the seven reconstructed specimens is shown one at a time with their lesion in purple, while their KC and KC densities are shown with the same colors used in Figures 1 and S1. Specimens are observed from the rostral part rotating alternatively from medial to lateral and lateral to medial as shown by the reference STR in the inset. Specimens are ordered from the one having the most posterior neurogenic response to the one displaying the most anterior one. From 1:53 all specimens are overlaid together highlighting that, on the whole, KCs cover most of the medial and rostral portion of the STR. In both Imaris and Blender, the 3D views are in orthographic mode.

The peak KC density was always far from the SVZ, in line with the STR origin of these structures (Figures 1C and S1A). The KC number varied greatly, ranging from 55 to 586 (mean ± SD = 288 ± 202), and correlated with their mean nearest neighbor distance (Figure 1F; Table S1, *p < 0.001*), indicating that stronger neurogenic responses resulted in increased foci density within similar areas. When all specimens were registered together, neurogenic areas collectively occupied most of the rostral and medial STR (Figures 1D, 1E, 1G–1I, S1A, and S1B; Video S1). In particular, the neurogenic foci were preferentially distributed in the medial STR (Figure 1H), an associative functional domain (Figures S1A′ and S1B′; Hintiryan et al., 2016). By contrast, these structures were more sparse in the lateral somato-motor STR and extremely rare in the caudal multi-modal domain (Figures S1C–S1E′).

In summary, STR neurogenic foci organize in a complex 3D germinal matrix whose spatial disposition is contingent on the lesion border. The potential of neurogenic foci induction is widespread in the STR with a higher probability in its medial domain.

### Neurogenic foci exist along a continuum of maturation profiles

**Figure 2:**
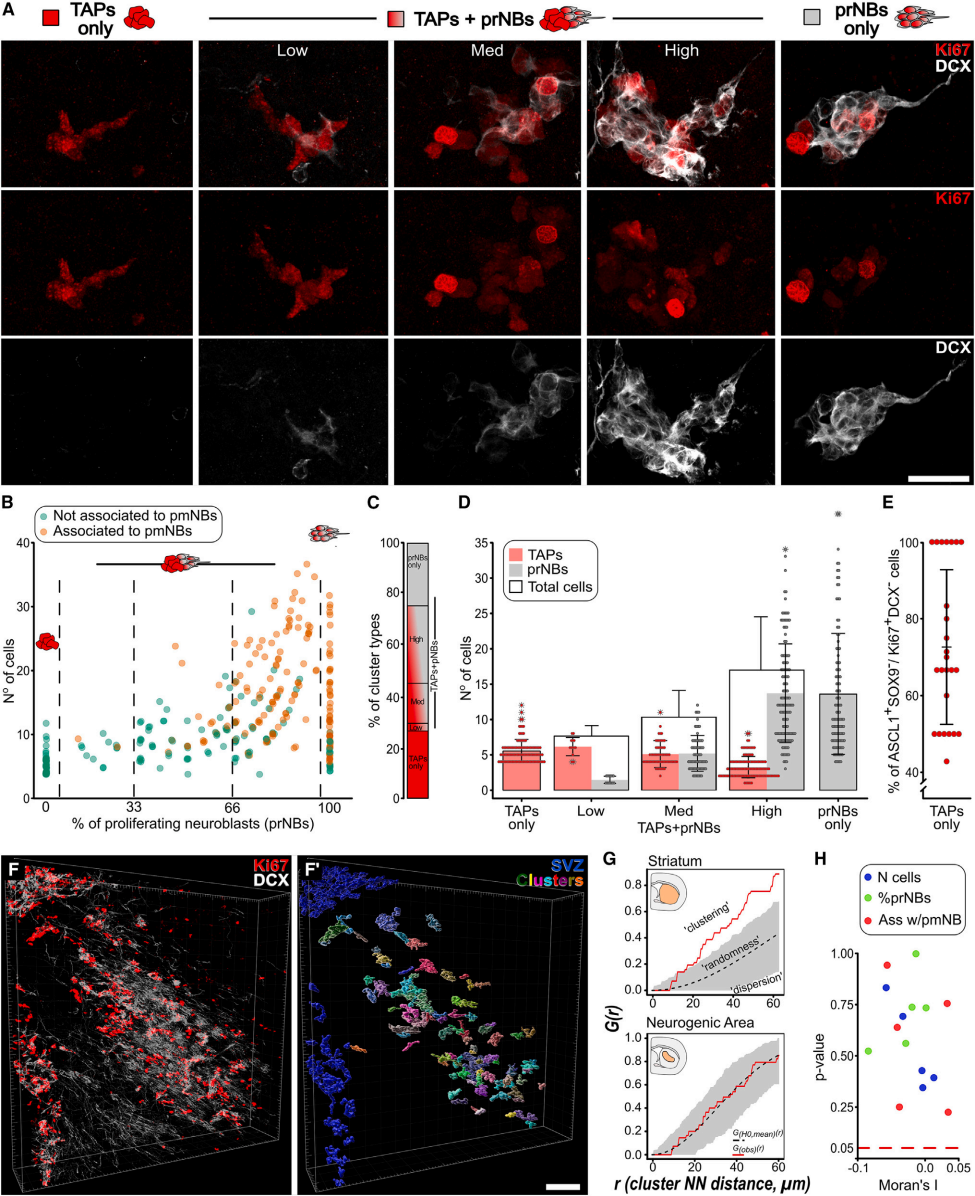
Neurogenic foci exhibit heterogeneous cellular composition and are spatially independent (A) DCX and Ki67 staining in KC subtypes. (B) n° of cells vs. percentage of prNBs per KC; KCs associated with pmNBs are in orange. (C) Percentage of KC types among reconstructed KC. (D) N° of TAPs (red) and prNBs (gray) per KC type (see also Table S1). (E) Percentage of ASCL1^+^ cells in TAPs-only (*n* = 25). (F and F′) 3D reconstruction of rostral neurogenic area and (F′) segmented KC in STR (random colors) and SVZ (blue) (see also Video S2). (G and H) Representative G functions of STR or neurogenic area KC distribution of the specimen in (F). Gray areas: pointwise envelopes of 999 simulations of complete spatial randomness. *G*_*(obs)*_*(r)* (red line): experimental value; *G*_*(H0,mean)*_*(r)* (black dashed line): mean of the simulations. (H) Moran’s I index and associated *p* value for each tested specimen (*n* = 5) and variable (*n* = 3) (see method details and Table S1). Data in (D) and (E) are expressed as mean ± SD. Scale: (A) 25 μm (F) 100 μm.

KCs have been described in different models of STR neurogenesis, but their individual cellular composition has never been resolved (Luzzati et al., 2006, 2011a; Magnusson et al., 2014; Nato et al., 2015). The size and fraction of TAPs and prNBs in these foci could (1) vary along a continuum of maturation profiles as in adult neurogenic niches or (2) could be more invariant as in stem cell systems, like the skin, where continuous activity at fixed locations maintains a stationary proportion of maturation stages (Altshuler et al., 2023). To distinguish between these models, here we evaluated the TAPs (DCX^-^Ki67^+^) and prNBs (DCX^+^Ki67^+^) content of 430 KCs 3D reconstructed from serial sections at 5 wpl (*n* = 8 mice; Figures 2A and S2A–S2E).

The KCs varied greatly in size, ranging from 4 to 38 cells (mean ± SD = 11.8 ± 7.6 cells), and in the proportion of TAPs and prNBs (Figures 2A and 2B). About half of the KCs were composed of only TAPs (TAPs-only KC) or only prNBs (prNBs-only KC) while the other half included a mix of the two cell types (TAPs+prNBs KC; Figures 2A–2C). We further subdivided TAPs+prNBs KCs into TAPs+prNBs_Low, Med, and High according to the prNB fraction (Figures 2A–2C). Both KC size and number of prNBs increased with the prNB fraction, progressing from TAPs-only to TAPs+prNBs_High KCs (Figure 2D; Table S1, *size: p < 0.001; prNBs*: *p < 0.001*). Conversely, TAPs exhibited an opposite trend (Figure 2D; Table S1, *p < 0.001*). The increase in prNB content and size further correlated with a higher probability of pmNBs (DCX^+^Ki67^-^) being part of the neurogenic foci (Figures 2B and S2F; Table S1, *percentage of prNBs: p < 0.001; size: p < 0.001*). Of note, in all the TAPs-only KCs, most of the cells expressed the TAP marker ASCL1 (Parras et al., 2004) but not the AS marker SOX9 (Cheng et al., 2009; Sun et al., 2017; Figures 2E and S2G–S2G′), confirming the neuronal commitment of these structures.

Neurogenic foci cellular composition thus varies along a continuous spectrum of transitions that may represent sequential stages of a common developmental process. KCs may be initially composed only of TAPs, progressively accumulate prNBs, generate pmNB, and ultimately deplete the progenitor pool. Alternatively, this variability may be contingent on spatial or temporal factors.

### Neurogenic foci are spatially independent

STR neurogenic foci heterogeneity could result at least in part from regional differences in lineage progression. To verify this possibility we performed a spatial analysis of 318 3D reconstructed KCs at 5 wpl (*n* = 5 mice; Figures 2F–2F′; Video S2). As expected, spatial point pattern analyses detected significant clustering of KCs along the lesion border (Figures 2G and S3A; *pooled p < 0.001*). On the contrary, within the neurogenic area, KCs were randomly distributed (Figures 2G and S3B; *pooled p = 0.229*). We next employed a measure of global spatial autocorrelation, the Moran’s I index, to understand if the KC size, prNB content, and association with pmNBs are distributed following specific spatial patterns (Figures S3C–S3C″). For all the specimens, the spatial distribution of these features did not deviate significantly from simulations of complete spatial randomness (Figure 2H; Table S1).

Video S2. High-resolution 3D reconstruction of a portion of the neurogenic area at 5 wpl, related to Figure 2 and S2 A portion of the neurogenic area of specimen #G14.3 is shown in this video. SVZ Ki67^+^ cell segmentation is shown in blue, while STR KC segmentation is shown in random colors to highlight that each of them is an independent structure. Starting from 24 s the random color segmentations are shown as transparent objects allowing to appreciate individual Ki67^+^ and/ or Ki67^+^DCX^+^ cells within each structure. KCs at different maturation stages are zoomed in at different time points: a TAPs-only KC at 10 s, a TAPs+prNBs KC at 25 s, and a prNB-only KC at 35 s

Hence, we conclude that KCs are randomly distributed in the neurogenic area and that their maturation profiles are spatially independent.

### Neurogenic foci number, distribution, and cellular composition are stable after neurogenesis onset

**Figure 3:**
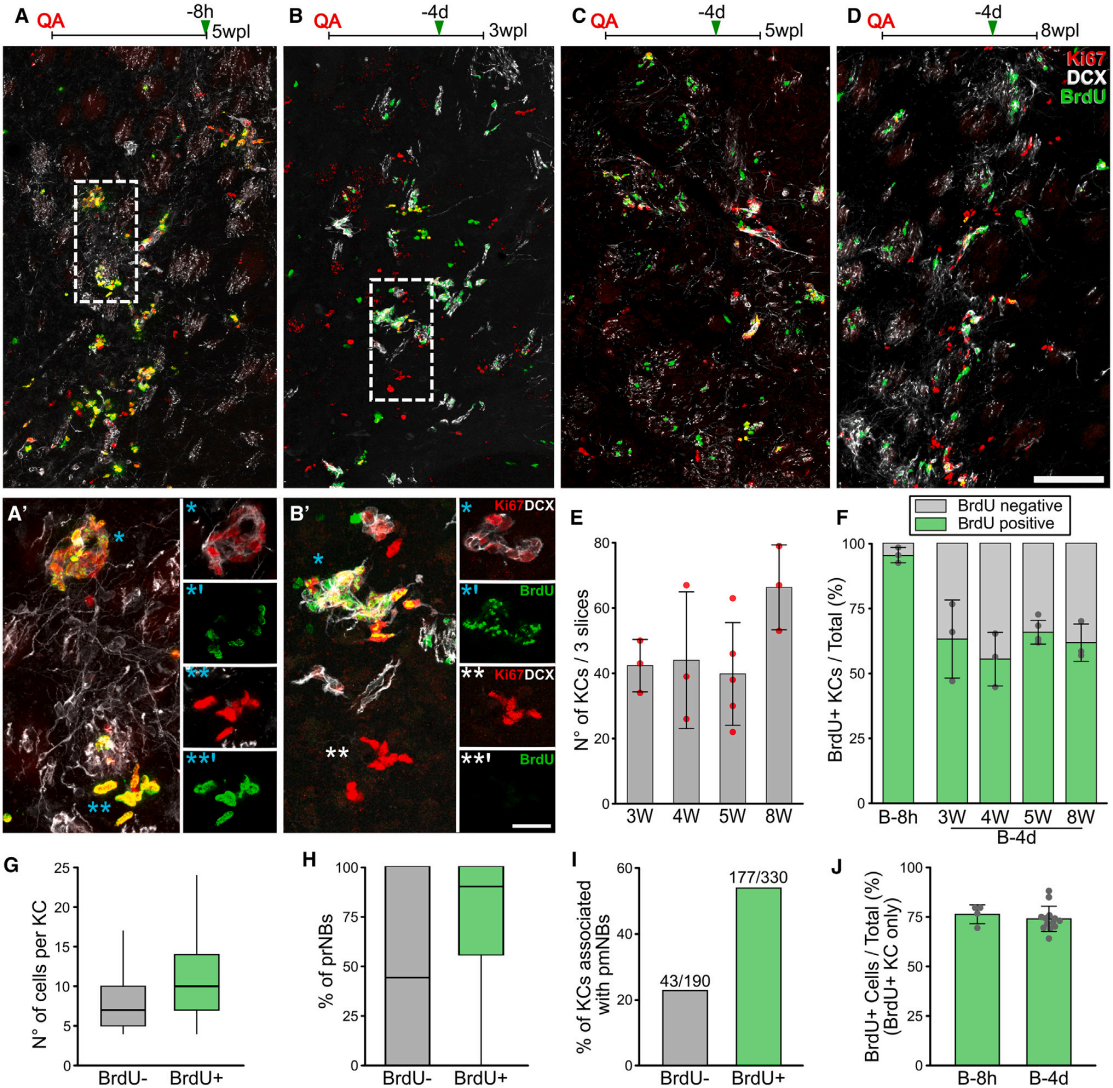
Neurogenic foci undergo a constant turnover (A–D) BrdU, Ki67, and DCX labeling from B-8h (A) and B-4d specimens at 3 wpl (B), 5 wpl (C), and 8 wpl (D) in the neurogenic core. (A′–B′) Higher magnification of (A) and (B). Maximum intensity (MAX) projections and single focal planes, respectively, in the left and right panels. ^∗^ indicates more mature and ^∗∗^ more immature KC. (E) n°r of KC in three non-consecutive slices. (F) Percentage of BrdU^+^ KC at B-8h and B-4d. (G–I) Comparison of the n° of cells (G), percentage of prNBs (H), and the fraction associated with pmNBs (I) per KC between the BrdU^-^ and the BrdU^+^ KC at B-4d. (J) Mean ± SD percentage of BrdU^+^ cells in BrdU^+^ KC in B-8h and B-4d specimens. Data in (E–F) and (J) are expressed as mean ± SD. Data in (G–H) are reporterd as box and whisker plots. See Table S1 for statistical analyses of (E–J). Scale: (A–D) 100 μm; (A′) and (B′) 20 μm.

To ascertain the temporal dynamics of STR neurogenesis, KC number, distribution, and composition were analyzed at 3, 4, 5, or 8 wpl. As observed at 5 wpl, KCs were always centered around the lesion border and their overall distribution did not vary in time (Figure S4A). Similarly, their total number remained stable over time (Figures 3B–3E and S4A; Table S1, *p = 0.157*). Of note, all the KC types described by the 3D reconstruction analysis (Figures 2A–2C) were identified in all specimens and time points and both their relative proportions and size were constant (Figures S4B–S4D; Table S1). These data show that, shortly after their appearance, between 2 and 3 wpl, the number, distribution, and composition of STR neurogenic foci reach a stable level that is maintained up to 8 wpl.

### Neurogenic foci undergo continuous turnover

The KCs stable heterogeneity suggests they are turned over. To assess KC turnover rates, we first established a saturating bromodeoxyuridine (BrdU) protocol. Two BrdU injections over 8 h (“B-8h” group) at 5 wpl labeled 97% ± 2% of the KCs (Figures 3A and 3F) and 74% ± 6% of their cells (Figures 3A′ and S4E) indicating that virtually all KCs are made of actively dividing cells. As in the SVZ (Ponti et al., 2013), TAPs had a slightly higher proliferation rate than prNBs (Figure S4F; Table S1, *p = 0.040*), independent of the KC maturation profile (Figure S4F; Table S1, *TAPs: p = 0.125*; *prNBs: p = 0.132*).

We then analyzed the fraction of BrdU^+^ KCs after a four-day chase at 3, 4, 5, or 8 wpl (“B-4d” groups; Figures 3B–3D). At all time points, about 40% of the KCs lacked BrdU^+^ cells (BrdU^−^ KCs, Figure 3F). Compared to BrdU^+^ KCs, the BrdU^−^ KCs exhibited smaller sizes, lower fractions of prNBs, and less association with pmNBs (Figures 3B′ and 3G–3I; Table S1, *p < 0.001 in each case*). The lack of BrdU^+^ cells and lower maturation profile indicate that BrdU^−^ KCs were established within four days after the BrdU injection. Interestingly, when only the BrdU^+^ KCs were considered, neither the percentage of BrdU^+^ cells (Figure 3J, *p = 0.319*) nor the frequency distribution of the BrdU^+^ cells per KC varied between B-8h and B-4d (Figure S4G; Table S1, *BrdU*^*+*^ *KCs*: *p = 0.247*). Thus, in the BrdU^+^ KCs, neither BrdU dilution under detection levels nor the addition of new TAPs from quiescent cells occurred in the four-day chase. Hence, BrdU^−^ KCs are not derived from loss of BrdU staining, but they actually represent newly formed KCs. To sum up, for at least 5 weeks, new neurogenic foci are continually established and undergo progressive maturation in the STR parenchyma. Since their number remains stable (Figure 3E), the genesis of new KCs must be balanced by the exhaustion of old ones. KCs are thus transient structures for which we calculated a lifetime of 10.6 ± 2.1 days and a turnover rate of 9.4%/day (Methods S1, section 5).

Together with the lack of spatial patterns in KC organization, these data suggest that in the neurogenic area KC initiation continuously occurs at random locations within a globally permissive environment.

### Clonal expansion of STR ASs generates individual KCs

AS could support the establishment of these transient KCs in several ways. Each KC could arise from individual or multiple ASs. In parallel, each ASs could expand locally to generate an individual clone or produce multiple migrating progenitors that secondarily expand. To distinguish among these possibilities, we conducted a clonal analysis of STR ASs.

**Figure 4:**
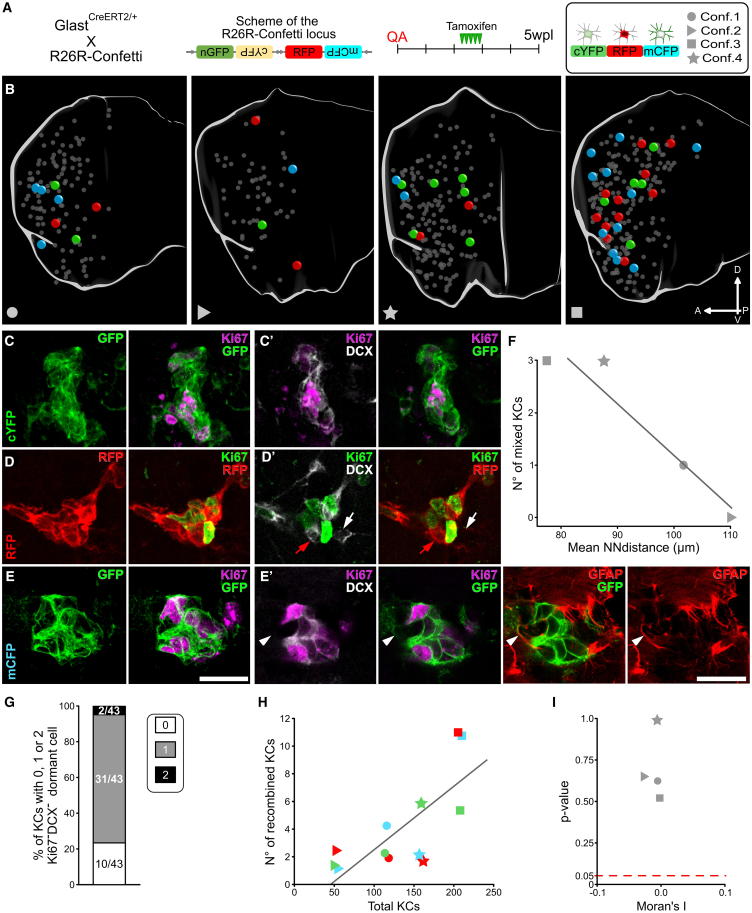
Clonal expansion of STR ASs generates individual KC (A) *R26R*-Confetti locus and experimental timeline. Right panel: reporter color and specimen symbols legend. (B) Medial view of the four 3D reconstructed STR. KCs are depicted as spheres, bigger and colored for Confetti KCs, smaller and gray for the others. (C–E) MAX projection of confetti KC: expressing cYFP, RFP, and mCFP, respectively. (C′–E′) Single focal planes of (C), (D), and (E), respectively. Red and white arrows in (D′): color-matching and unlabeled pmNB, respectively. Arrowhead in (E′): dormant GFAP^+^ AS. (F) Correlation between the mean KC nearest neighbor distances and the n° of mixed KC. (G) Percentage of Confetti KC containing 0, 1, or 2 dormant Ki67^−^DCX^−^ cell. (H) Correlation between the total n° of KC and the n° of KC expressing each reporter. (I) Moran’s I index and associated *p* value calculated for each specimen (see method details and Table S1). Black lines in (F) and (H) represent linear regression models. Scale: (C–E′) 20 μm.

*Glast*^CreERT2/+^ mice expressing the inducible form of CRE recombinase under the astrocytic promoter *Glast* (Mori et al., 2006) were crossed with *R26R*-Confetti reporter mice (Snippert et al., 2010) in which tamoxifen (TAM) administration leads to the stochastic expression of 1 out of 4 fluorescent proteins (Figures 4A and S5A). At 5 wpl, 15 days after TAM administration, 2.4% ± 0.7%, 1.2% ± 0.7%, and 1.7% ± 1.4% of the SOX9^+^GFAP^+^ STR ASs expressed RFP, mCFP, and cYFP, respectively (Figures S5B–S5E, see also S5I–S5J).

Whole STR 3D reconstruction of four specimens (Figure 4B) led to the identification of 537 KCs (mean ± SD = 134 ± 63), 49 of which showed reporter expression (Confetti KCs; 8.4% ± 3.3%; Figures 4C–4E′). The Confetti KC maturation profiles matched those observed at 5 wpl (Figure S5K). Among Confetti KCs, 42/49 were clones of cells expressing the same reporter (87% ± 11%; Figures 4C–4E′) and thus originated from the same AS. Non-recombined cells represented only 2% of all Confetti KC cells (25/1,048 of total counted cells) and were confined to 7 KCs. These few mixed KCs were found in the specimens with the lowest mean KCs nearest neighbor distance (Figure 4F; Table S1, *p = 0.042*), suggesting that increased density favored the rare fusion of neurogenic foci.

Interestingly, about 75% of Confetti KCs included a clonally related Ki67^−^DCX^−^ cell, rarely two, (Figure 4G) that in 70% of the cases we could confirm being a GFAP^+^ AS (Figure 4E′; 10/14 tested cells). Overall, these results demonstrate that STR KCs derive almost exclusively from the clonal expansion of a single AS and suggest that it often remains associated with its progeny in a dormant state.

Further, we addressed if a single AS can give rise to multiple KCs. The number of KCs sharing the same reporter in each specimen was extremely low, ranging from 1 to 11 (mean ± SD = 4.1 ± 3.6). However, the same-color KCs were still within the expected values given the number of KCs in that specimen (Figures 4H and S5F–S5H; Table S1, *p = 0.004*). Moreover, Confetti KCs sharing the same reporter did not show any evident spatial association (Figure 4B), as confirmed statistically by Moran’s I and by permutation tests (Figures 4I; Table S1). This indicates that KCs are clonally independent structures deriving from distinct ASs. The presence of a dormant AS within the neurogenic foci (Figures 4E′ and 4G) further supports this conclusion and indicates that these cells locally expand. STR neurogenic activity thus closely resembles that of canonical niches but is distributed over a much wider 3D environment, thus leading to a negligible intermixing of different AS progeny.

### STR ASs activate at a constant rate

**Figure 5:**
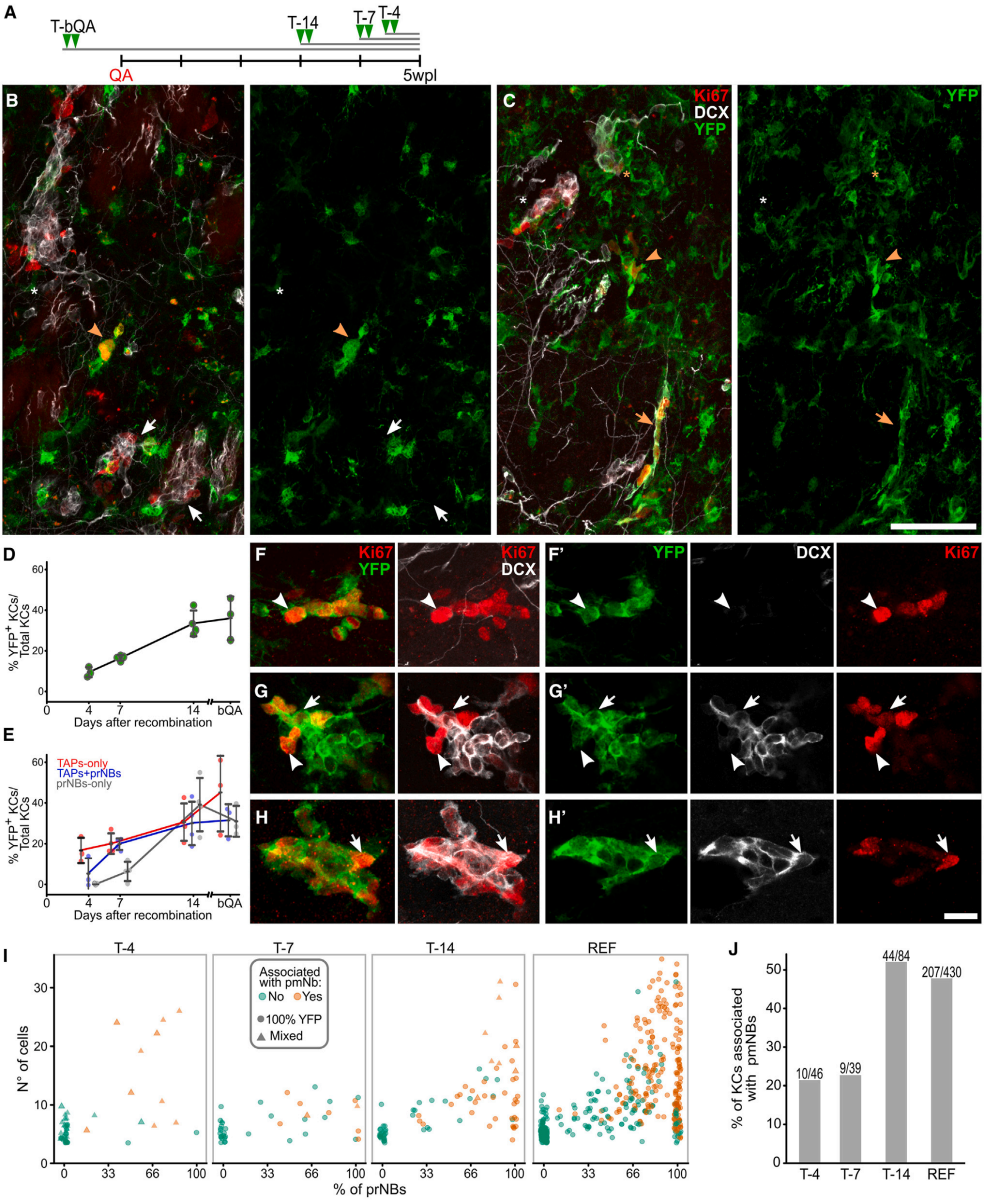
Constant AS activation generates TAPs-only KCs that autonomously mature overtime (A) Experimental timeline. (B and C) YFP^+^ and YFP^−^ KC in T-4 (B) and T-14 (C). Arrowheads: TAPs-only; asterisks: TAPs+prNBs; arrows: prNBs-only. White and orange: YFP^−^ and YFP^+^ KC, respectively. (D) LI by TAM administration of all KC or (E) divided by KC type. (F–H) MAX projections of completely YFP^+^ TAPs-only (F), TAPs+prNBs (G), and prNBs-only (H) KC. (F′–H′) Single focal planes of (F–H), respectively. Arrowheads: TAPs; arrows: prNBs. (I) Percentage of prNBs vs. n° of cells per KC at different time points (see also Figures S6A and S6B). KCs associated with pmNBs are in orange. REF as in Figure 2B. Circles: fully recombined KC; triangles: mixed YFP^+^ and YFP^-^ KC. (J) Percentage of KC associated with pmNBs at different time points and in the REF (see also Table S1). Data in (D) and (E) are shown as mean ± SD at each time point (see also Table S1). Scale: (B and C) 50 μm; (F–H′) 15 μm.

BrdU analysis did not detect any cellular turnover in neurogenic foci, strongly suggesting that AS activation initiates new KCs that subsequently autonomously expand and mature. To validate this model, we used lineage tracing by dating back the AS activation events contributing cells to 5 wpl KCs. Different cohorts of *Glast*^CreERT2/+^x*R26R*-YFP animals were sacrificed at 5 wpl and received TAM 4, 7, or 14 days before sacrifice (T-4, T-7, and T-14; Figure 5A). At each time point we measured the KC labeling index (LI), that is, the fraction of KCs that received at least one YFP^+^ cell from their AS progenitor (YFP^+^ KCs; Figures 5B and 5C). As a reference for the maximum LI, we analyzed specimens receiving TAM before the lesion (T-bQA; Figure 5A). The KC LI linearly increased from 9.4% ± 2.5% in T-4 to 33.5% ± 6.3% in T-14 and remained stable in T-bQA (36.1% ± 10.9%; Figure 5D; Table S1, *p < 0.001*; *T-4* vs*. T-14: p = 0.002*; *T-14* vs*. T-bQA: p = 0.938*). This linear increase in the YFP^+^ KC proportion means that AS activation occurs in a staggered manner at a constant rate (see mathematical analysis in the following) until it reaches a plateau: around 14 days are needed for all the 5 wpl KCs to have experienced at least one AS activation event.

### Each AS activation event initiates a new KC that progressively matures

To explore if AS activation preferentially occurs in specific phases of the KC life, we evaluated the LI of the main KC subtypes across time points (Figures 5E–5H′). The LI of TAPs-only KCs was already at plateau at T-4 (Figures 5E–5F′; Table S1, *p* = 0.359), showing that TAPs-only KCs are on average within four days from an AS activation event. By contrast, TAPs+prNBs and prNBs-only KCs showed a delayed increase reaching their peaks only by T-7 and T-14, respectively (Figures 5E and 5G–5H′; Table S1, *p = 0.006, p = 0.001*). This indicates that these KC types are progressively more distant in time from the last AS activation event. In line with these observations, the number of Ki67^+^ cells, the fraction of prNBs, and the association with pmNBs progressively increased with time (Figures 5I, S6A, S6B, and 5J; Table S1, *p < 0.001 in each case*) and became indistinguishable from the reference population by T-14 (Figures 5I, S6A, S6B, and 5J; Table S1, *T-14 vs. REF: p=0.249, p=0.549, and p=1, respectively*). Thus, STR AS activation predominantly occurs in the initial phases of KC life, giving rise to TAPs-only KCs which then gradually mature.

**Figure 6:**
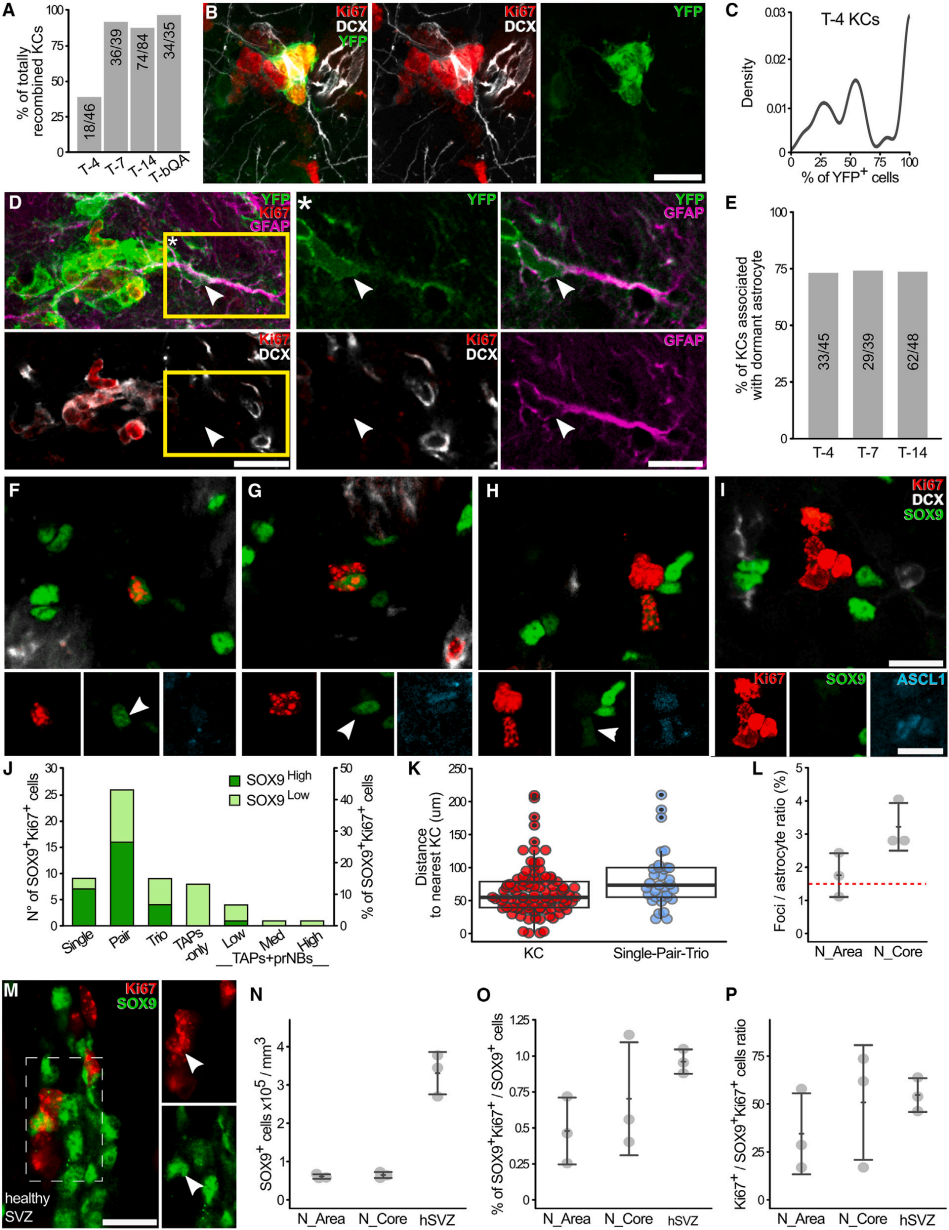
ASs activate in KC-free areas and shortly come back to quiescence (A) Percentage of fully YFP^+^ over total YFP^+^ KC. (B) Immature mixed KC at T-4. (C) Frequency distribution of the fraction of YFP^+^ cells per KC at T-4. (D) YFP^+^ KC associated to a dormant GFAP^+^ AS. (^∗^) single focal plane of the yellow box in (D). (E) Percentage of KC associated with at least a YFP^+^ dormant cell over time. (F–I) Single (F), pair (G), trio (H), or clustered (I) Ki67^+^ cells in which at least 1 proliferating cell is SOX9^+^. The lower panels show SOX9 and ASCL1 expression in Ki67^+^ cells. Arrowheads: SOX9^High^ cells; arrows SOX9^Low^ cells. (J) n°(left) and percentage (right) of Ki67^+^SOX9^High^ and SOX9^Low^ cells in Ki67^+^ groups. (K) Comparison of the distance to the nearest KC between KC (red) and single/pair/trio (gray). (L) Foci/AS ratio in the neurogenic area (N_Area) and in its core (N_Core). Red dashed line: AS activation rate in the SVZ (Calzolari et al., 2015). (M) A SOX9^High^Ki67^+^ cell (arrowhead) in the SVZ of a healthy mouse. Left panel: MAX projection. Right panel: single focal plane of the dashed rectangle. (N–P) Comparison of the AS density (N), the percentage of proliferating ASs (O), and the ratio between TAPs/prNBs and proliferating ASs, among STR neurogenic areas and healthy SVZ. Data in (K) are reported as box and whisker plot. Data in (L) and (N–P) are expressed as mean ± SD. Scale: (B), (D), (F–I), and (M) 15 μm.

To further validate that AS activates only at KC initiation, we examined the prevalence of YFP labeling in YFP^+^ KCs (Figure 6A). In contrast to T-14 and T-7, at T-4 only 40% of the YFP^+^ KCs were 100% recombined (18/46; Figures 6A, S6A, and S6B; Table S1). These 100% YFP^+^ KCs were almost exclusively small TAPs-only KCs (Figure S6D). Interestingly, with the exception of 5 putative fused KCs (Figures S6C–S6C′), the mixed KCs (including YFP^+^ and YFP^−^ cells) had only slightly more mature profiles suggesting they have a similar age as the 100% YFP KCs (Figures 6B, S6A, S6B, S6E–S6G, and S6I; Table S1). As most mixed KCs contained about 30%–60% of YFP^+^ cells (Figure 6C), we can infer that in these KCs *Glast*^CreERT2/+^-driven recombination occurred at 2- or 3-cell stage in ASs or their earliest progeny. These results confirm that AS activates virtually exclusively to initiate a new KC. As for Confetti mice, at all time points a Ki67^-^DCX^-^ dormant cell, in rare cases two, clustered with 75% of YFP^+^ KCs (Figures 6D and 6E), and it turned out to be GFAP^+^ in 26/27 tested cases (Figure 6D). Thus STR ASs rapidly return to quiescence after division and their reactivation is extremely rare, at least within KC life.

A mathematical analysis of KC initiation dynamics (Methods S1, section 1) shows that the initial slope of the increase of the KC LI over time corresponds to the KC initiation rate (Figure 5D), which also applies to the AS activation rate. Given that this slope is approximately linear, we can conclude that the AS initiation rate is approximately constant. When approaching the KC lifetime, the KC LI is expected to saturate to a plateau (Methods S1, section 1). This occurs at around 10–14 days, consistent with the previously estimated KC lifetime of 10.6 days. These results thus conclusively demonstrate that KCs undergo steady-state turnover and provide direct proof that ASs initiate new KCs that progressively mature before undergoing exhaustion.

### ASs activate in neurogenic foci-free areas

The aforementioned data predict that ASs activate in KC-free areas. To directly verify this possibility, we analyzed the organization and distribution of proliferating ASs in the neurogenic area at 5 wpl (Figures 6F–6I, S6I, and S2G–S2G′). We distinguished between cells expressing SOX9 at similar or slightly lower levels as the non-proliferating ASs (SOX9^High^) from those showing substantially lower levels (SOX9^Low^; Figures 6F–6I) that may represent TAPs (Cheng et al., 2009). Most Ki67^+^SOX9^+^ cells were in groups smaller than four cells with a strong prevalence for pairs (Figures 6G and 6J). The fraction of SOX9^High^ cells among Ki67^+^SOX9^+^ cells progressively decreased from 78% in individual cells to 62% in pairs, 44% in trios, and 7% in KCs (Figures 6F–6I). This decrease was paralleled by an increase in ASCL1 expression (Figure S6H; Table S1, *p < 0.001* in both cases) confirming that early neurogenic foci cells lose their astrocytic identity before reaching the KC stage.

The groups of less than four Ki67^+^ cells containing activated ASs never contacted pmNBs, and their nearest KC was on average farther away than the mean nearest neighbor distance between KCs (Figures 6K and S6I; Table S1, *p = 0.013*). These results show that STR ASs preferentially activate in areas devoid of pre-existing KCs and far from previous activation events.

### STR and SVZ ASs activate at similar rates

Neurogenic foci represent activation events that occurred over the last 10 days. To evaluate STR AS activation rate, we thus evaluated the KC/AS ratio in the neurogenic area or its core, respectively, comprising 95% or 25% of the KCs. The AS density did not differ between these areas, and, although it tended to be higher than in the rest of the STR (Figure S6I), it was still about 5 times lower than in the SVZ of healthy mice (hSVZ; Figures 6M and 6N; Table S1, *N_Area vs. hSVZ: p = 0.0001, N_Area vs. hSVZ: p = 0.0001*). The KC/AS ratio ranges from 1% to 4%, corresponding on average to 0.18% activations per day in the neurogenic area, a value that is strikingly similar to that calculated for SVZ ASs (0.15% activations per day; Figure 6L; Calzolari et al., 2015). In support of this observation, the fraction of proliferating ASs (Ki67^+^SOX9^+^/SOX9^+^ cells) as well as the ratio between these cells and clustered Ki67^+^ cells in SVZ and STR were also similar (Figures 6O and 6P; Table S1, *p = 0.168*, *p = 0.52*). STR ASs thus share with SVZ ASs not only the activity pattern but also the activation rate, implying a comparable prevalence of NSC potential.

### Reactivity of neurogenic ASs

Neurogenic activation and reactivity are independent AS states that can co-occur, particularly after acute lesions inducing AS proliferation (Sirko et al., 2013). To unveil the overall relationships between these states after QA, we analyzed the AS reactive response. At 1 wpl, STR ASs were all strongly reactive: they were hypertrophic, and virtually all upregulated GFAP, NESTIN, and C3 (Figure S7A–S7K; Escartin et al., 2021; O’Shea et al., 2024). Inside the lesion they further strongly proliferated at this stage (Figures S7A–S7C; Table S1). Interestingly, at 1 wpl, C3 was transiently upregulated also in virtually all SVZ ASs, indicating that it is compatible with the neurogenic state (data not shown). At 5 wpl, while generic features of AS reactivity such as GFAP expression were still present, NESTIN and to a lesser extent C3 expression was decreased and mostly confined to the lesion and bordering intact tissue (Figures S7F–S7K; Table S1). In this area, both proliferating SOX9^+^ and *Glast*-CreERT2::YFP^+^ quiescent SOX9^+^ ASs associated with YFP^+^ KCs were similarly heterogeneous for the expression of C3 as their SOX9^+^ neighbors (Figures S7I–S7O). Thus, as in other models of acute lesions (Escartin et al., 2021; O’Shea et al., 2024), QA causes an early strong proliferative AS reactivity that subsequently matures in border ASs showing a reduced but persistent reactive state. Neurogenically active ASs are part of the overall AS reactivity, but interestingly their activation probability does not perfectly coincide with the early AS proliferation gradient being more equally distributed around the lesion border (Figure S7D). Activation of the neurogenic program is thus likely compatible with multiple reactive AS states, at least after QA.

### Cell fate choice timings in the turnover of neurogenic foci

As ASs divide mainly once per KC, TAPs and prNBs account for most of the NB production. The autonomous growth and steady-state turnover of STR neurogenic clones allowed us to exploit our rich collection of reconstructed KCs (Figure 2) to model TAPs and prNBs expansion and fate choices. Intermediate progenitors are usually thought to expand stereotypically; however, their behavior is still poorly characterized (Götz, 2018). We implemented a stochastic mathematical model (Haccou et al., 2007; Methods S1) to discern (1) how the differentiation propensity (TAP→prNB; prNB→pmNB transitions) varies over time and whether it depends on KC ages and (2) whether the differentiation of TAPs and prNBs is coupled to cell division via asymmetric and symmetric divisions, or whether it can occur independently of cell divisions.

**Figure 7:**
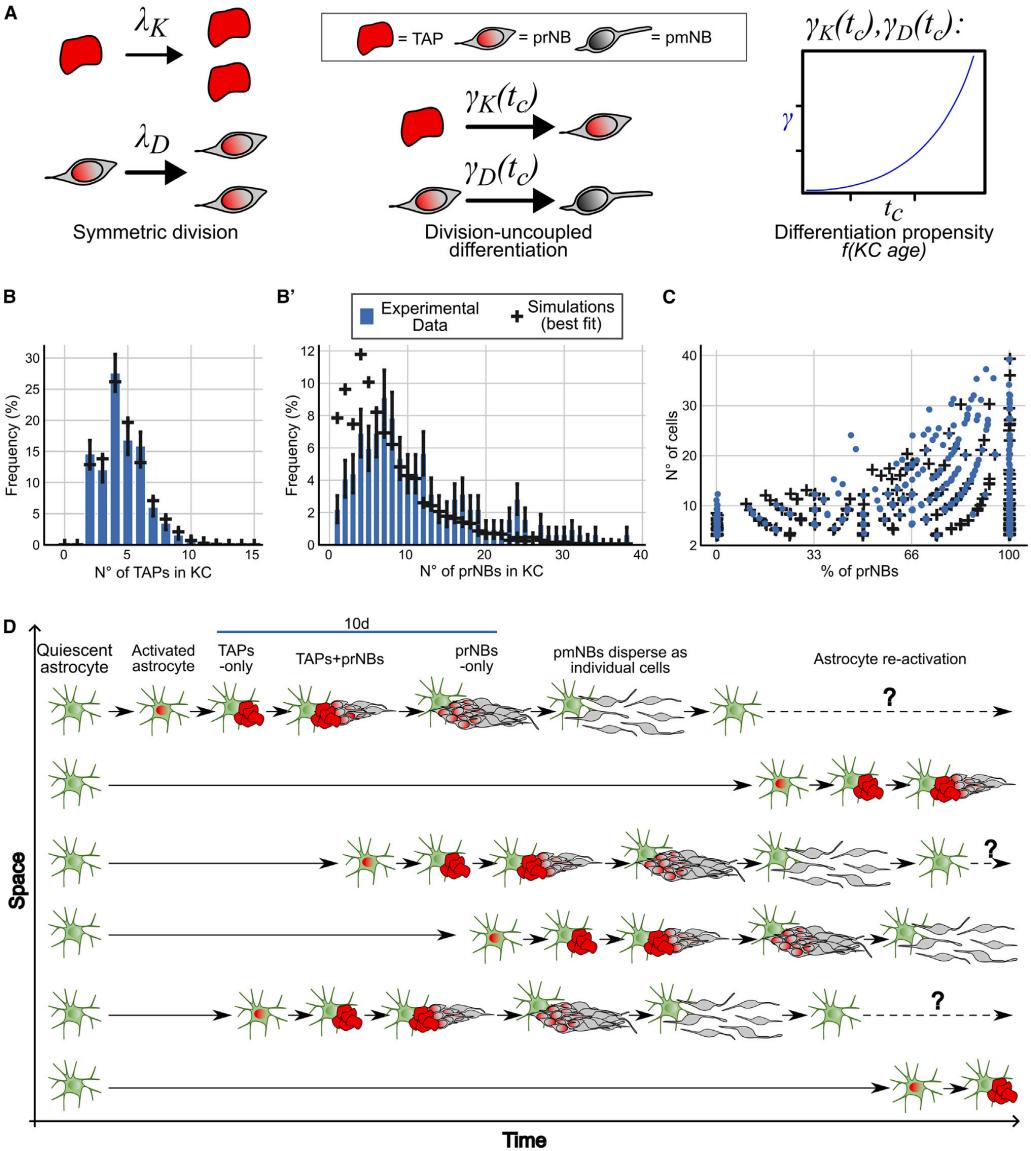
Mathematical modeling of cell fate choices in KC (A) Schematics of the best fitting model features. TAPs and prNBs mostly undergo symmetric duplicating division with *λ*_*K*_ and *λ*_*D*_ rates, respectively. The differentiation propensities of TAPs and prNBs (*γ*_*K*_ and *γ*_*D*_, respectively) change as a power law function over the KC age. (B and B′) Best model fitting of TAPs (B) and prNBs (B′) frequency distributions in KC. Bars are measured frequencies, and black crosses represent simulation output, obtained from the best fitting model (see also Methods S1). (C) Scatterplot showing the n° of cells vs. percentage of prNBs per KC. Similar to Figure 2B, with the addition of simulated KC obtained from the best fitting model (data not used for fitting the model). (D) Scheme of the spatiotemporal dynamics of STR AS activation and lineage progression.

Models of the distribution of TAPs per KC showed an excellent fit by allowing cell fate choices to be stochastic, yet only if the differentiation propensity increases in an accelerated way over time (Figures 7A and 7B; Methods S1; section 4.1.3). The best fit is achieved if we assume that differentiation can occur independently of cell division although we cannot strictly exclude the option that differentiation is coupled to symmetric cell division (Methods S1, sections 4.1.2 and 4.1.3).

As for TAPs, only if the prNB differentiation propensity increases over time in an accelerated way, do we obtain a reasonable model fit (Figures 7A and 7B′; Methods S1; section 4.2). However, for prNBs, we can exclude that differentiation is coupled to cell division; thus, the exit of the cell cycle occurs independently of the previous division. We note that some deviations between data and model predictions remain even for the best fit, for very small and very large cell numbers, respectively, over- and underrepresented in the model (Figure 7B′). This is likely due to the dispersion of the last prNBs within clustered NB at KC end (very small) and KC fusions (very large).

Neurogenic foci cells thus show features of both stochastic and deterministic fate choices that ultimately result in their invariant exit from the cell cycle but with consistent variability. Hence, these data fully validate the presented model of neurogenic foci initiation, maturation, and dynamic turnover (Figures 7C and 7D) and mechanistically confirm the intermediate progenitor nature of the KC cells.

## Discussion

Here, we demonstrated that the spatiotemporal pattern of STR AS neurogenic activity closely resembles that of classic adult vertebrate brain niches. However, these patterns emerged within the 3D lattice of parenchymal ASs, distinguishing them from conventional NSC monolayers. STR AS activation events were constant in time, at a rate similar to SVZ ASs, and random in space resulting in a steady state of continuous and widespread neurogenesis. The mouse STR can thus be considered as a dormant neurogenic niche hosting a widespread population of qNSCs that can be focally activated.

### NSC activation dynamics in the STR neurogenic niche

The local expansion and minimum overlap of AS proliferating progeny allowed us to use neurogenic foci as a proxy for the location of ASs activation events over the last ∼10 days. By integrating two complementary dynamic approaches, we unveiled that ASs neurogenically activate at a constant rate. In parallel, the neurogenic foci spatial independence revealed that activation events are randomly distributed in space and time. These events preferentially occur in new locations, far from pre-existing foci, overall demonstrating the widespread presence of neurogenically competent ASs within the STR. A similar random pattern of NSC activations inhibited by previous events was described in zebrafish (Dray et al., 2021b). In that case, inhibition was proposed to be mediated by NBs, which inhibit NSC activation also in mammalian niches through γ-aminobutyric acid (GABA) release or cell-cell contacts (Liu et al., 2005; Rolando et al., 2012). The fact that proliferating STR ASs rarely contact NBs supports the hypothesis that similar inhibitory mechanisms might be at play in the STR. Following activation, in both SVZ and SGZ, NSCs undergo consuming neurogenic divisions with high probability (Calzolari et al., 2015; Obernier et al., 2018; Pilz et al., 2018), while asymmetric self-renewing divisions characterize neurogenesis during aging (Bast et al., 2018; Harris et al., 2021). This latter mode of division is preferred also by STR ASs, ensuring the maintenance of the population. Of note, a recent study suggests that in the SVZ NSCs divide always symmetrically and daughter cells locally compete for the occupancy of “a restricted niche” (Basak et al., 2018). According to this model, the lower AS density in the parenchyma may increase the chance of at least one cell reoccupying the niche. The division of TAPs and NBs was regulated by stochastic processes although an accelerating differentiation propensity introduces a deterministic factor constraining their expansion. Similar stochastic behavior of TAPs has been described in the SGZ by *in vivo* imaging (Götz, 2018; Pilz et al., 2018).

### Neurogenic permissiveness of the STR parenchyma

Our results demonstrated that the STR acts as a neurogenic niche by constitutively hosting a widespread population of dormant NSCs. Notably, all major known NSC quiescence stimulating pathways such as NOTCH (Imayoshi et al., 2010), BMP (Lim et al., 2000), β1 INTEGRIN (Porcheri et al., 2014), S1P (Codega et al., 2014), or GABA (Liu et al., 2005; Song et al., 2012) are active in parenchymal AS (Lim et al., 2000; Magnusson et al., 2020; Nagai et al., 2019; Robel et al., 2009; Singh et al., 2022). Unlike canonical niches, in the parenchyma, inhibition of these pathways alone mostly fails to induce neurogenic activation. Nonetheless, at least for NOTCH inhibition, single-cell RNA-seq indicates induction of a primed NSC state (Magnusson et al., 2020; Zamboni et al., 2020). Acute lesions inducing ASs to proliferate and upregulate immature markers such as NESTIN can also reduce NSC quiescence in both canonical niches (Llorens-Bobadilla et al., 2015), and parenchyma (Sirko et al., 2023; Zamboni et al., 2020). Only in the striatum after QA, stroke, and to a lesser extent NOTCH inhibition (Magnusson et al., 2014; Nato et al., 2015) however, these state transitions progress to neurogenic activation *in vivo*, suggesting that additional signals are needed to regulate this switch. After QA, neurogenic ASs were likely part of the early reactivity response and still show some reactive features during the neurogenic phase, such as GFAP, NESTIN, and some C3 expression. Interestingly, however, both lesioned and intact sides of the lesion border, originally associated with strong or no AS proliferation, can host neurogenic activation later on. Activation of the neurogenic program is also temporally dissociated from this early proliferative response as it starts during the third wpl, which may be consistent with the tissue remodeling phase (Burda and Sofroniew, 2014). This suggests that neurogenic activation is compatible with multiple AS reactive states and could be regulated by specific factors. Moreover, although neurogenic potential was present throughout the STR, it was more likely to activate in the medial functional domain. Interestingly, this domain showed a higher frequency of neurogenic foci also in normal rabbits (Luzzati et al., 2006), under progressive degeneration (Luzzati et al., 2011a), stroke, or NOTCH inhibition (Magnusson et al., 2014). Activatory stimuli diffusing from the SVZ have been proposed to cause this spatial bias (Magnusson et al., 2020); however, at least after QA the pattern of activation events was not shaped as an SVZ centered gradient but peaked more laterally, at the lesion border. This suggests the involvement of local factors preferentially associated with the overlap between the medial striatum and the mature lesion border. Whether the medial striatum has a higher density of dormant NSCs or is subjected to stronger stimulating factors remains to be determined. The existence of such factors is supported by the high variability in neurogenic foci number in both pathological (this work) and physiological (Luzzati et al., 2006) conditions. An intriguing possibility is that activation factors may be related to neuronal afferents, which have been shown to modulate neurogenic activation in neurogenic niches (Káradóttir and Kuo, 2018) and represent the main structural difference between the striatal domains.

### How widespread is the neurogenic potential among ASs?

NOTCH pathway blockage causes widespread NSC priming in cortical and STR ASs (Magnusson et al., 2020; Zamboni et al., 2020), leading the authors to speculate that all AS possess a neurogenic potential. Here, we demonstrate that naive STR ASs do have such widespread potential. Establishing whether all STR ASs harbor NSC potential is challenging, a question that is unresolved even for the canonical NSC (Dray et al., 2021a; Obernier et al., 2018). The close similarity in both the pattern and rate of neurogenic activation indicates that these two populations have comparable prevalence of NSCs. However, as in both cases the reactivation rate is unknown, the exact AS fraction that is recruited to neurogenesis cannot be established. In STR, ASs do not reactivate at least over the KC lifetime, but their quiescence likely lasts longer because (1) clusters of pmNBs remain for some time after proliferative pool exhaustion (data not shown) and (2) AS activate far from pre-existing KC. Moreover, the local activation density may be much higher than the average, as suggested by cases of KC fusion. Thus, the maximum density of recruited ASs, a proxy of the neurogenic potential per unit area, is likely much higher than our measure over 10 days (up to 1/25 ASs), potentially including all ASs.

In non-mammalian vertebrates, radial glia persists into adulthood where they act both as NSC and AS-like support cells (Jurisch-Yaksi et al., 2020). In mammals, it was thought that these two functions were fulfilled by distinct cell types and that AS differentiation implies a permanent loss of NSC potential. However, our results definitely demonstrate that this potential can be maintained in parenchymal ASs. Whether all AS subtypes/states have the same probability to activate an NSC potential, both between and within regions, remains to be established. NSCs are a heterogeneous population of progenitors committed to generate specific neuron types and, at least in adults, are regulated by distinct conditions and neuronal circuits (Chaker et al., 2023; Káradóttir and Kuo, 2018). Within the mammalian parenchyma, ASs, while specializing in local circuit-specific control, may have also acquired a circuit-specific regulation of their neurogenic capacity.

We already showed activation of a dormant niche in the ventral STR around weaning in guinea pigs, but, similarly to the dorsal SVZ, this niche was confined to the narrow pallial-subpallial-boundary (Luzzati et al., 2014). Our new study now extends the complexity of adult NSC spatial heterogeneity to the 3D lattice of parenchymal ASs. This radically challenges a traditional view of neurogenic niches as unique domains for stem cell maintenance acting as barriers to parenchymal differentiation signals. The brain may rather have a widespread quiescent neurogenic potential organized as a mosaic of progenitor domains regulated by specific activation signals. In canonical niches, these signals are constitutively active, while in other regions they may be triggered only in specific conditions. The size, distribution, regulation, and cell fate potential of these progenitor domains await further analysis, thereby paving the way for a deeper understanding of the full adult brain neurogenic potential.

### Limitation of the study

We could not precisely measure how many ASs harbor an NSC potential, yet distinguishing between reversible state transitions of multipotent cells and epigenetically constrained subpopulations is a major unsolved issue in AS and NSC biology. Our data provided the first indication for the dynamic regulation of a functionally defined AS reactive state, specifically the neurogenically active state, and suggest that it acts as an independent module. However, more detailed analyses of the spatiotemporal dynamics of other functionally defined AS states/modules will be required to understand their neurogenic potential and more in general to define the limits and regulation of AS plasticity.

## Experimental procedures

### Experimental model and subject details

#### Animal procedures

The experimental plan was designed according to the guidelines of the European Communities Council (2010/63/EU) and the Italian Law for Care and Use of Experimental Animals (DL26/2014). It was also approved by the Italian Ministry of Health (authorization 327/2020-PR) and the Bioethical Committee of the University of Turin. The study was conducted according to the Animal Research: Reporting of In Vivo Experiments guidelines.

#### Mouse lines

Experiments were performed on 8- to12-weeks-old animals of the following mouse lines: C57BL/6J mice (Harlan Laboratories; *n* = 18 males), *Glast*^CreERT2^ (Mori et al., 2006), *R26R*-YFP (Srinivas et al., 2001), and *R26R*-Confetti (Snippert et al., 2010).

### Method details

#### Stereotaxic injections

Mice were anesthetized with 0.3 mL/kg ketamine (Ketavet, Gellini) and 0.2 mL/kg xylazine (Rompun, Bayer), positioned in a stereotaxic apparatus (Stoelting) and injected with a pneumatic pressure injection apparatus (Picospritzer II, General Valve Corporation). Injection coordinates are as follows: QA (1 μL; diluted to 120 mM in 0.1 M PB), +0.1 mm AP, −2.1 mm ML, and −2.6 mm DV.

#### BrdU pulse labeling

C57BL/6J lesioned mice received two intraperitoneal injections of BrdU (Merck; 50 mg/kg in 0.1 M Tris pH 7.4) 6 h apart and were sacrificed either 2 h (B-8h) or 4 days (B-4d) after the last injection. Animals of the B-8h group were sacrificed at 5 wpl while B-4d at 3, 4, 5, or 8 wpl.

#### TAM administration

TAM (Merck) was dissolved in corn oil (Merck) and administered by oral gavage at a dose of 2.5 mg per administration with a 24-h interval. *Glast*^CreERT2/+^x*R26R*-YFP mice received 2 TAM administrations, while *Glast*^CreERT2/+^x*R26R*-Confetti mice received 5 administrations.

#### Histology and immunofluorescence staining

Coronal sections of 50 μm were obtained and immunostained as described previously (Nato et al., 2015, see also supplemental information). Images were acquired on a Leica SP5 confocal microscope (Leica Microsystems) equipped with 40× and 63× objectives (HCX PL APO lambda blue: 40×, NA 1.25; 63×, NA 1.4). Voxel size is 0.76 × 0.76 × 2.5 μm, for whole striatal 3D reconstruction, 0.38 × 0.38 × 1.5 μm for AS and KC analyses, and 0.24 × 0.24 × 1 μm for GFAP staining.

#### 3D reconstructions

3D reconstructions were performed by modifying a previous method (Luzzati et al., 2011b). For detailed protocol, see supplemental information.

#### Imaris visualization

Videos S1 and S2 and Figures 1B–1B‴ and 2F–2F′ were prepared in Imaris by importing the Ki67 and DCX channels of aligned #N1 and #G14.3 specimens in Imaris (v9.7.2). After adjusting brightness and contrast, the SVZ was manually drawn in order to exclude the fluorescence in that area. KCs in Videos S1 and S2 and Figures 1B′–1B‴’ and 2F′ were segmented semi-automatically based on fluorescence intensity, object size, and striatal location.

#### Data visualization

See supplemental information.

### Statistical analysis

#### Quantifications

See supplemental information.

#### Spatial statistics (point pattern analysis)

To define KC distribution, we used the G(*r*) function. To analyze the 3D distribution of KC features, we used the Moran’s I index of global spatial autocorrelation (see supplemental information).

## Resource availability

### Lead contact

Further information and requests for resources and reagents should be directed to the corresponding author, Federico Luzzati (federico.luzzati@unito.it).

### Materials availability

This study did not generate any unique reagents.

### Data and code availability

Primary data and information to re-analyze them are available from the lead contact upon request. The collection of scripts originally created to perform the 3D reconstructions is available at the following link: https://github.com/bunbunet/FogliNato2024_3Drec.

## Acknowledgments

We thank Sara Trova and Alessia Caramello for preliminary work; Valentina Cerrato, Stefano Zucca, and Ilaria Bertocchi for critically reviewing the manuscript; and reviewer 2 for urging the analysis of AS reactivity. This research was supported by: 10.13039/100015558Fondazione Cecilia Gilardi, 10.13039/501100004710Fondazione Umberto Veronesi, funding from PNRR MUR – M4C2 – Investimento 1.4 to P.P., the 10.13039/501100007601European Union’s Horizon 2020 research and innovation programme under grant agreement no. 874758 to A.B., funds of the University of Turin and Compagnia di San Paolo (S1618 grant) to A.B. and F.L., MIUR project “10.13039/100017336Dipartimenti di Eccellenza 2018–2022 and 2023–2027 to Dept. of Neuroscience “Rita Levi Montalcini.”, and UKRI Medical Research Council grant (MR/R026610/1) to P.G.

## Author contributions

Conceptualization, M.F., G.N., and F.L.; software – 3D reconstructions, F.L.; investigation, M.F. and G.N.; formal analysis, M.F., G.N., and F.L.; spatial analyses, M.F.; mathematical modeling, P.G.; formal analyses – BrdU time course, J.P.; AS proliferation time course, M.R.; 3D reconstruction assistance, G.V.; writing – original draft, M.F., G.N., P.G., and F.L.; writing – review and editing, M.F., G.N., P.G., P.P., A.B., and F.L.; visualization, M.F., G.N., and F.L.; supervision, P.P., A.B., and F.L.; funding acquisition, P.P., A.B., and F.L.

## Declaration of interests

The authors declare no competing interests.

## Supplemental information

Document S1. Figures S1–S7, Table S1 and supplemental experimental procedures

Methods S1. Detailed description of mathematical modeling campaign

Document S2. Article plus supplemental information
