# Supplementary material for: Dynamic spatiotemporal activation of a pervasive neurogenic competence in striatal astrocytes supports continuous neurogenesis following injury
Source: Stem Cell Reports. 2024 Sep 19;19(10):1432–50. doi: 10.1016/j.stemcr.2024.08.006 (PMC11561465; doi:10.1016/j.stemcr.2024.08.006)
Supplement: Document S1. Figures S1–S7, Table S1 and supplemental experimental procedures [file mmc1.pdf]

Stem Cell Reports, Volume *19*

Supplemental Information

Dynamic spatiotemporal activation of a pervasive neurogenic competence in striatal astrocytes supports continuous neurogenesis following injury

Marco Fogli, Giulia Nato, Philip Greulich, Jacopo Pinto, Marta Ribodino, Gregorio Valsania, Paolo Peretto, Annalisa Buffo, and Federico Luzzati

Supplemental figures

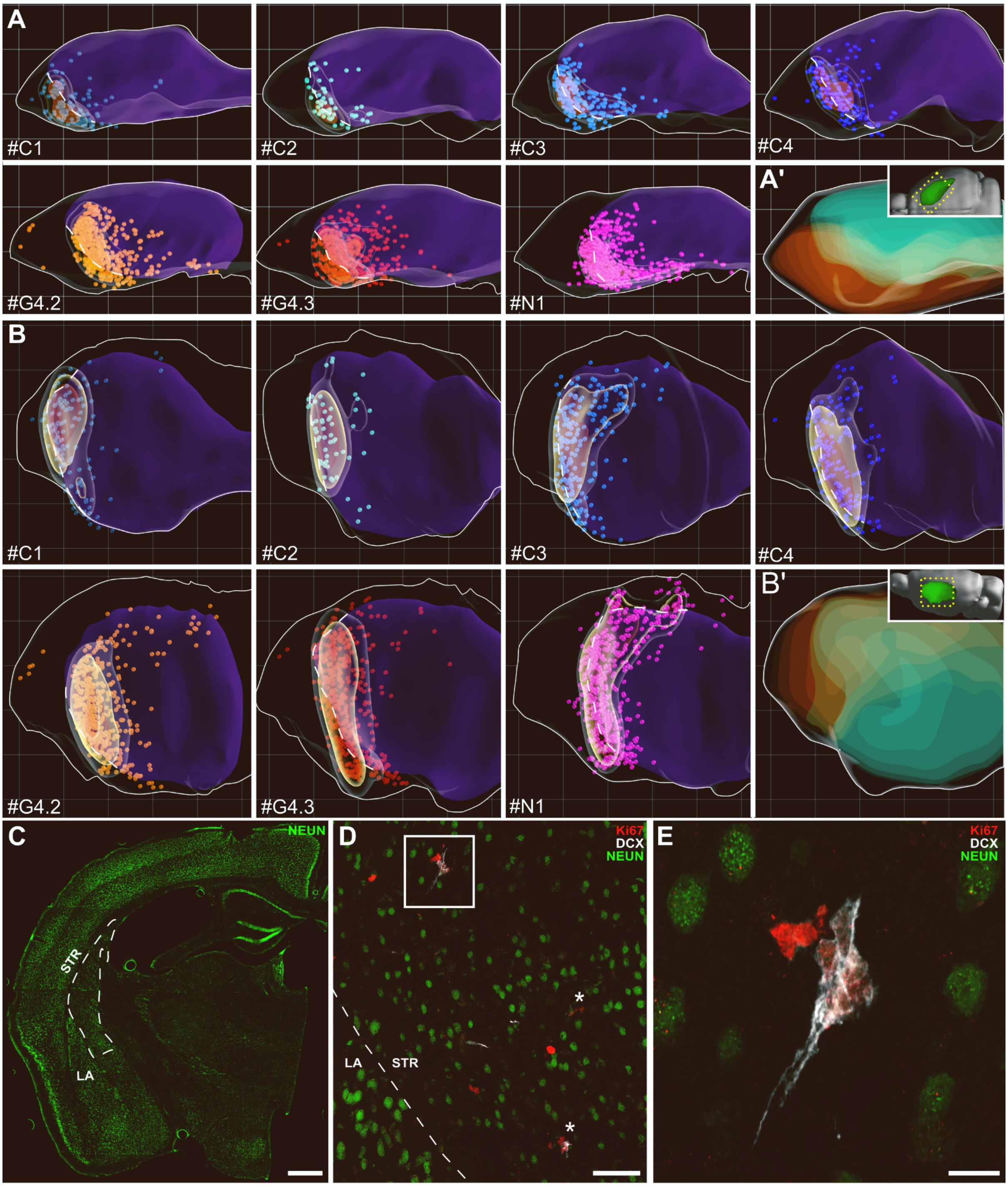

Figure S1. Neurogenic foci distribution in individual specimens, related to Figure 1

(A) Dorsal and (B) Medial views of the 3D reconstructions of the KCs over the entire STR of 7 specimens registered to a common reference space (see also Figures 1D-1E and Video S1). The lesion is in purple. A white dashed line shows the lesion

border in the neurogenic area. Dots: KCs (25 µm diameter) colour-coded as in Figures 1D-1I. Increasing KC density is rendered as transparent, yellow and orange volumes. (A') Dorsal and (B') medial view of a reference STR with the associative functional domain of the striatum, receiving projections from the anterior cingulate cortex, in orange and the somato-motor domain, receiving afferents from the somatosensory cortex, in cyan. It highlights the medial and lateral striatum, respectively (3D reconstructed from Hintiran et al. 2016, see Method details). Grid lines are 500 µm spaced. (C-E') In a few specimens displaying a strong neurogenic response and partially intact caudal STR, rare KCs were found also at this level, corresponding to the caudal multimodal STR domain. These few KCs were observed on the posterior margin of the lesion border, where spared neurons were present. (C) Section labeled for neuronal marker NEUN (green) at the level of the caudal STR (dashed-line). (D) Higher magnification of the same section showing also Ki67 (red) and DCX (white) staining, highlighting the presence of KCs (asterisks). LA: Lateral Amygdala. (E) Higher magnification of the KC in the rectangle in (D). Scale: (C) 500 µm; (D) 50 µm; (E') 10 µm.

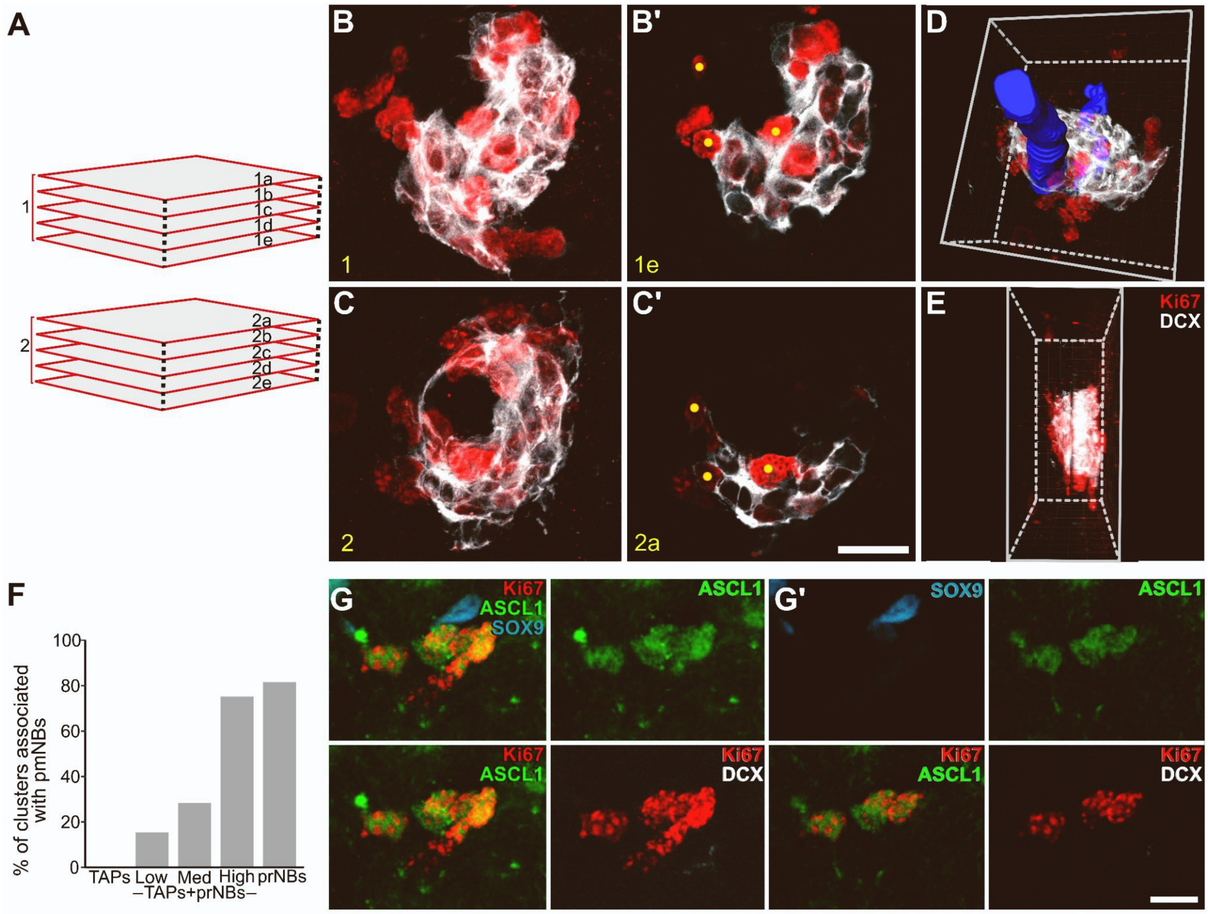

Figure S2. Method of high resolution 3D reconstruction and cellular composition of KCs, related to Figure 2

(A) Schematic view of the focal planes of two confocal stacks acquired from two consecutive sections. (B,C) Maximal intensity projection of sections 1 and 2 showing a KC splitted between the two sections. (B',C') The last intact focal plane (the section surface is usually not perfectly flat) of section 1 and the first one of section 2, respectively. Yellow dots highlight corresponding Ki67^{+} cells in the two sections. (D) Imaris 3D rendering of the reconstructed KC. A blood vessel was manually segmented and shown in blue. (E) Side view of the reconstructed KC. (F) Percentage of KCs associated with pmNBs subdivided by KC types. Note that TAPs-only KC were never associated with pmNBs, while the probability of this association progressively rose from TAPs+prNBs_Low to prNBs-only (Table S1, *p<0.001*). (G,G') TAPs-only KC stained for ASCL1 and SOX9. (G) Maximal intensity (MAX) projection; (G') Single focal plane. Scale: (B-C') 15 µm; (G,G') 5 µm.

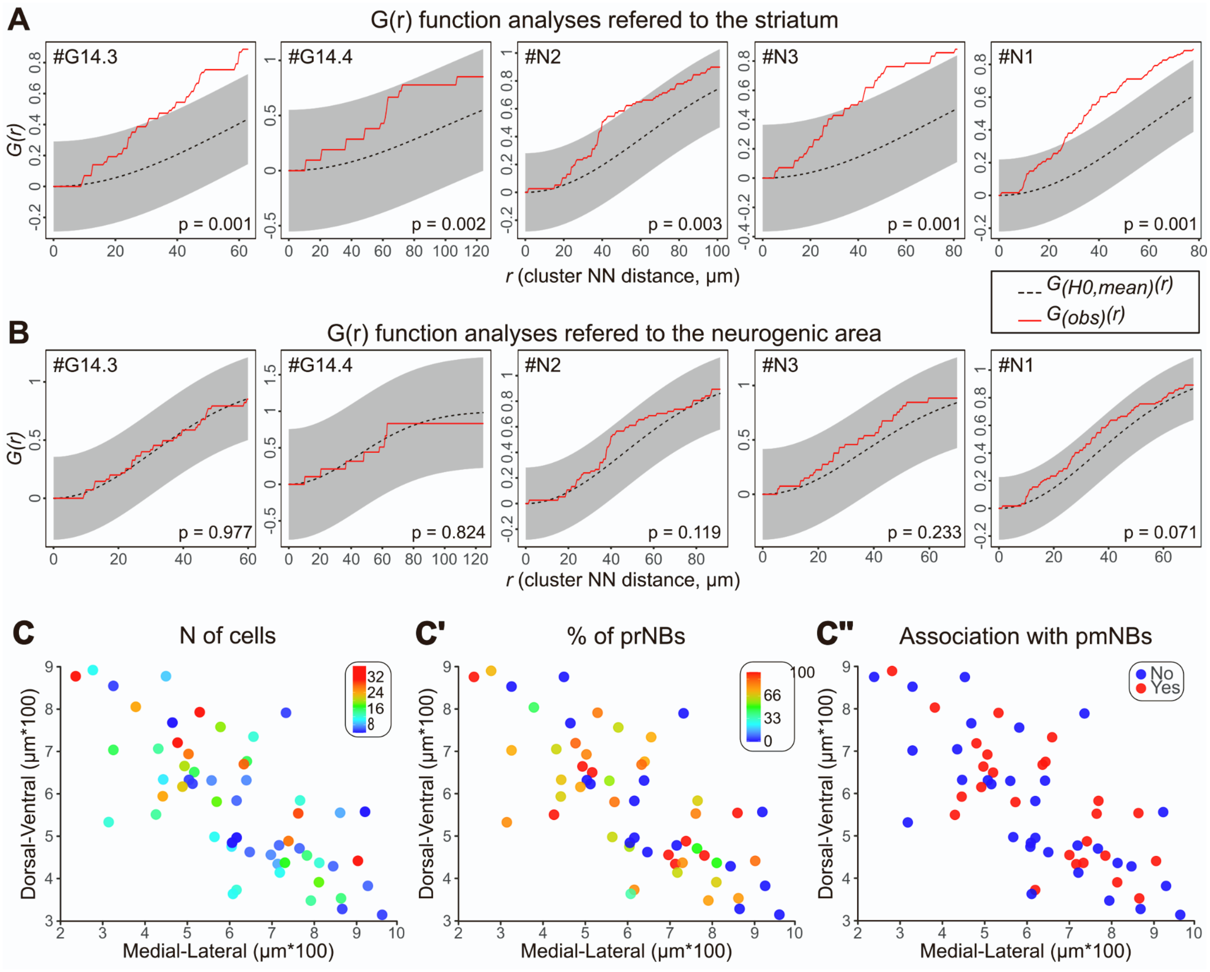

Figure S3. Spatial analysis of KC distribution, related to Figure 2

(A,B) G function analyses of KC distribution in respect of the striatum (A) or the neurogenic area (B) in five specimens. Red lines: $G_{(obs)}(r)$, functions calculated from the experimental data. Black dashed lines: $G_{(H0,mean)}(r)$, mean functions of the simulations of complete spatial randomness (*H0* = homogeneous Poisson process). Grey areas: 95% confidence envelopes. Unlike in Figure 2G, here simultaneous - instead of pointwise - envelopes were constructed so that we could reject the null hypothesis if the $G_{(obs)}(r)$ function lay outside the envelope at any value of *r.* As an additional approach for statistical comparison, the Diggle-Cressie-Loosmore-Ford (DCLF) test was performed and the resulting p-values are reported for each specimen and area (see also Method details). (C-C'') X-Y projection of KCs shown in Figure 2F coloured by size, percentage (%) of prNBs and association with pmNBs.

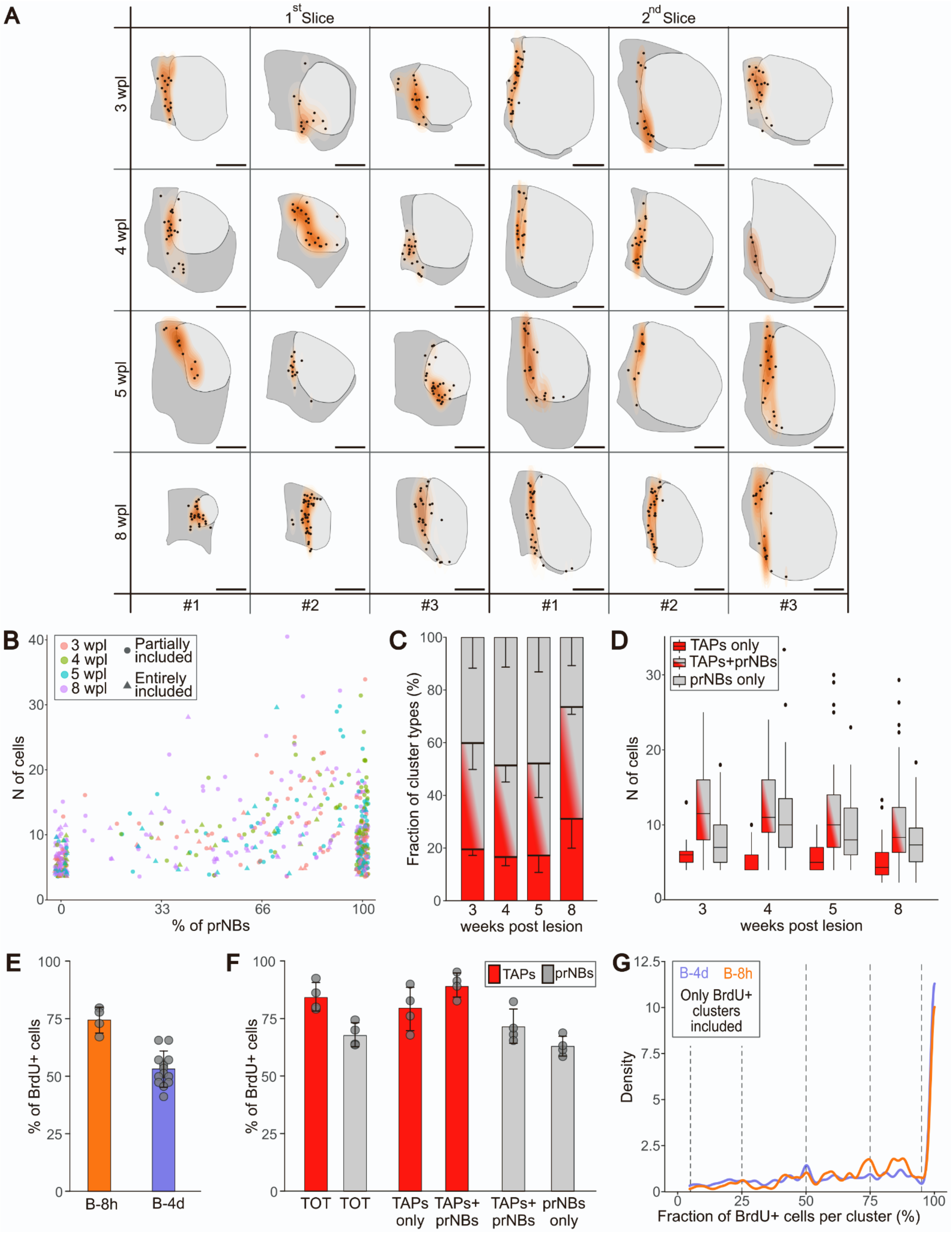

Figure S4. KC distribution and cellular composition are stable after neurogenesis onset, related to Figure 3

(A) Schematic view of the KC distribution in the two more anterior sections of the three analysed. Only three specimens (#) per group were included in this representation. Each KC is shown as a dot and the kernel density estimate of KC distribution is shown on a graded orange scale. Higher KC density corresponds to

higher orange intensity. Of note, the highest KC density is always ob-served at the border between the lesioned (light grey) and spared (darker grey) striatal tissue. (B) Percentage of prNBs vs number of cells in each KC. The colour of the dots indicates the group from which those KCs belong, while the shape indicates if the KC was partially (circle) or entirely (triangle) included in the analysed sections. (C) Relative fraction of KC types at each time point (Table S1, *TAPs-only:* $p = 0.049$, *TAPs+prNBs:* $p = 0.873$, *prNBs-only:* $p = 0.213$). Only concerning TAPs-only KCs, we found a significant difference overtime. Yet, post hoc analyses revealed a barely significant difference only when comparing 5wpl with 8wpl (Table S1, $p = 0.047$). The low number of specimens and the high variability may explain this small difference. (D) Number of cells per KC at different time points. Data were splitted into the three main KC types and shown as box and whisker plots to highlight the data distribution (Table S1, *TAPs-only:* $p = 0.468$, *TAPs+prNBs:* $p = 0.530$, *prNBs-only:* $p = 0.073$). Outliers are shown as black dots. (E) Percentage of $BrdU^+$ cells among all the KC cells in B-8h and B-4d groups. (F) Percentage of $BrdU^+$ TAPs (red) or prNBs (grey) in the whole population (TOT) or by KC type (TAPs-only, TAPs+prNBs, prNBs-only) in the B-8h group. (G) If only the $BrdU^+$ KCs are considered, the relative distribution of the % of $BrdU^+$ cells per KC is similar between the B-8h and B-4d groups (Table S1, $p = 0.247$). If BrdU dilution under detection level or cellular turnover occurred at substantial levels in KCs, we would expect a progressive leftward shifting of the orange curve. Thus, these analyses strongly support that neither BrdU dilution under detection levels, nor cellular turnover occurred within KCs. Thus, $BrdU^-$ KCs observed at each time point are newly formed structures. Data in (C), (E) and (F) are expressed as mean ± SD. Dots in (E-F) refer to individual specimens. Data in (D) are reported as box and whisker plots, where dots indicate outliers. Scale: (A) 500 µm.

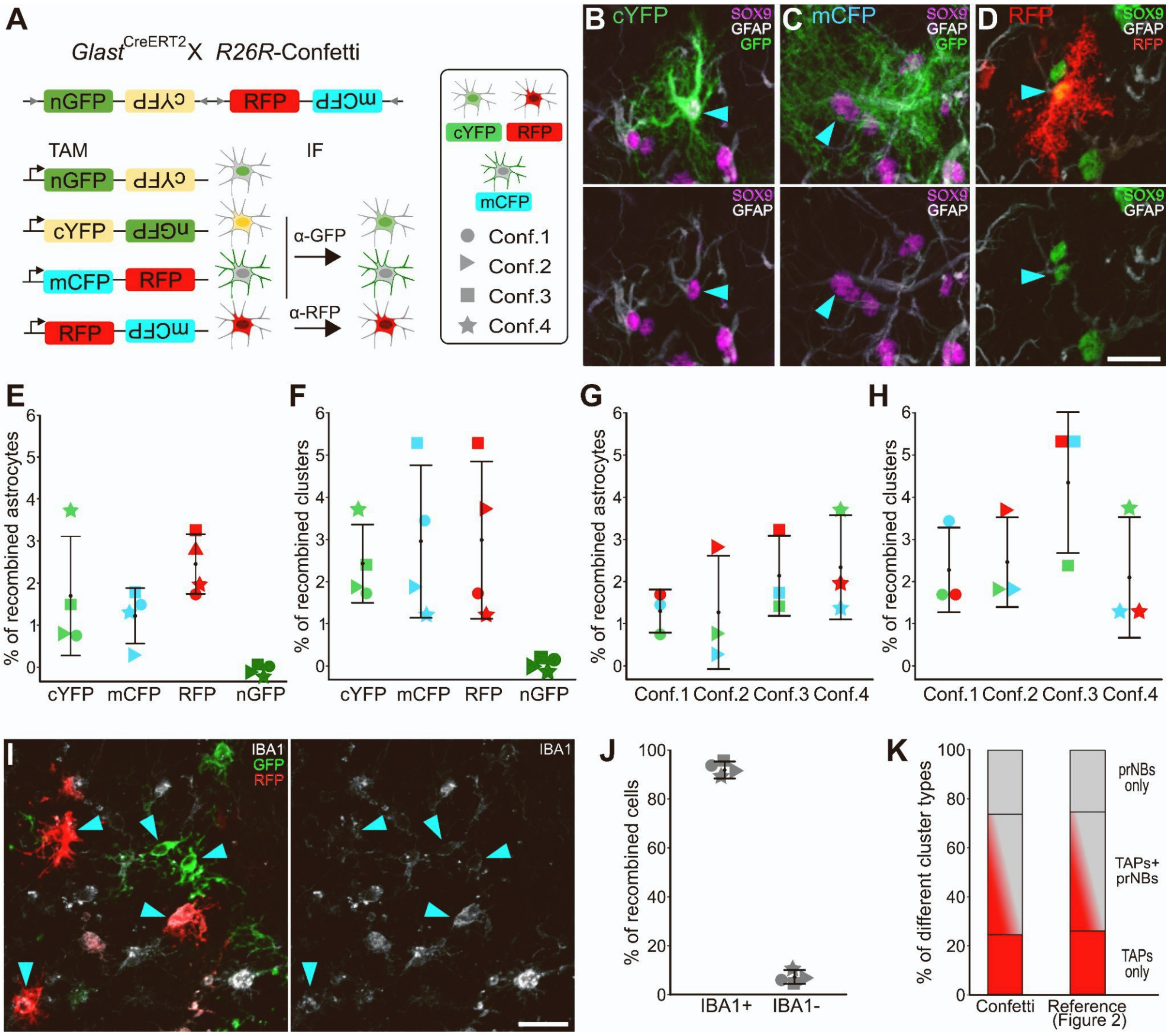

Figure S5. Confetti reporters are expressed homogeneously among striatal ASs, KCs and microglial cells of different specimens, related to Figure 4

A) Schematics of the *R26R*-Confetti locus. Tamoxifen (TAM) administration induces the stochastic and non-combinatorial expression of 1 of the 4 reporters. After immunostaining with α-GFP and α-RFP antibodies recombined cells showed either a cytoplasmic GFP staining (cYFP), a membrane-localised GFP staining (mCFP) or an RFP staining (RFP). In line with previous reports, we never observed the nGFP reporter (Calzolari et al., 2015). Each specimen is represented with a different symbol throughout the figure. (B-D) Representative images of recombined SOX9^{+}GFAP^{+} striatal ASs. (E) Fraction of SOX9^{+}GFAP^{+} cells (n = 1597 ASs, n = 4 mice). (F) KCs expressing the cYFP, mCFP or RFP (n = 49 KCs; n = 4 mice). (G,H) Same as (E,F) but different specimens are on the x-axis to show that they have similar recombination efficiency of striatal ASs and KCs (see also Table S1). The representations from (E) to (H) show that the fraction of striatal ASs and KCs expressing the different Confetti reporters were similar between reporter and specimens. (I) Consistent with the higher confetti recombination efficiency of confetti allele recombination in non-neural tissues (Snippert et al., 2010) we observed numerous IBA1^{+} (grey) microglial cells, that also express *Glast*, expressed the confetti reporters (cyan arrowheads). As demonstrated by this image these cells are

easily recognized by their morphology. (J) Fraction of recombined cells (GFP^+ and RFP^+ pooled together) expressing the microglial marker IBA1. (K) Relative frequencies of KC maturation profiles in the Confetti KCs and the reference population of KCs analysed in Figure 2, (Table S1, *TAPs-only: p = 1; TAPs+prNBs: p = 0.454; prNBs-only: p = 0.301)*. Data in (E-H) and (J) are expressed as mean ± SD; dots refer to individual specimens. Scale (B-D) and (I) 20 µm.

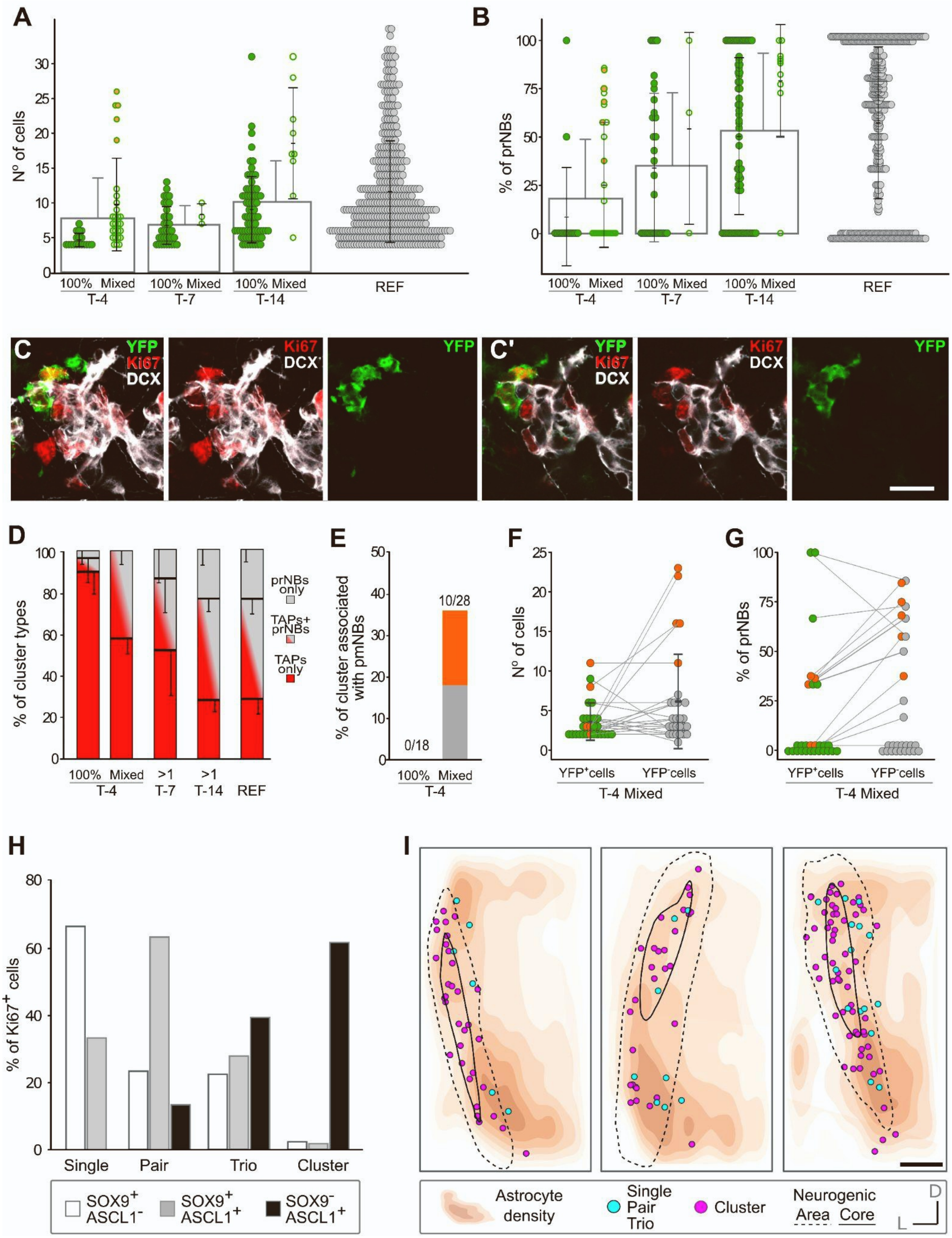

Figure S6. Striatal ASs activate only at the beginning of neurogenic foci life, related to Figures 5 and 6

(A,B) Number of Ki67^{+} cells and % of prNBs per KC. The data of the lineage tracing groups (T-4, T-7 and T-14) were compared with the data from the reference population of STR KCs analysed in Figure 2 ("REF", see also Table S1). At each

time-point, data from different specimens were pooled together and shown as mean (white box with grey contour) ± SD. The data are also reported splitted between KCs entirely composed of YFP$^+$ cells (“100%”) and those composed of a mixture of YFP$^+$ and YFP$^-$ cells (“Mixed”). Like in Confetti mice, all the Mixed KCs in T-7 and T-14 likely represent KC fusion as they displayed a more advanced maturation profile than their 100% counterparts and their number correlated with the mean nearest neighbour distance among KCs, a measure of KC density (*Regression analysis with exponential decay fit: p = 0.004*). Concerning T-4 Mixed KCs, only 5 could be derived from KC fusion for the above mentioned criteria (the orange ones, as in Figures S6E-S6G; see also Figure 5I). All others displayed only slightly more advanced maturation profiles than the 100% YFP$^+$ ones. Further, their number did not correlate with the mean nearest neighbour distance among KCs (*Regression analysis with exponential decay fit: p = 0.957*) suggesting that fusion due to high KC density cannot explain all the T-4 mixed KCs. (C,C’) Representative image of one of the five T-4 KCs that likely resulted from KC fusion. (C) MAX projection including the entire KC while (C’) single focal plane in which it is easier to see which cell is YFP$^+$ and which one is not. (D) Relative proportion of different KC types among YFP$^+$ KCs. T-4 KCs were splitted into 100% YFP$^+$ and Mixed KCs, while in other groups all KCs with at least 1 YFP$^+$ cell (>1) were considered together. Data are shown as mean ± SD among specimens. The 100% T-4 KCs are the most immature ones, while the T-4 Mixed resemble the T-7 but are much more immature than T-14 and REF KCs. (E) Fraction of KCs associated with pmNBs among 100% YFP$^+$ and Mixed KCs of T-4. (F) Number of cells and % of prNBs (G) among the YFP$^-$ and the YFP$^+$ cells of T-4 Mixed KCs. Each dot represents a KC. Of note, when the five putatively fused KCs were excluded from the analyses, we did not observe any difference in the number of cells and in the % of prNBs among the YFP$^+$ and YFP$^-$ compartments of T-4 Mixed KCs (Table S1). These data indicate that both groups of cells originated almost simultaneously at the beginning of the KC life. This is in line with the hypothesis that striatal ASs activate only at the beginning of KC life and with the possibility that *Glast*^{CreERT2} recombination occurs up to the 2- or 3-cell stage. (H) Percentage of Ki67$^+$ cells organised as single, pair, trio or KCs that express only SOX9 (white), SOX9 and ASCL1 (grey) or only ASCL1 (black). (I) AS density map (increasing orange intensity) and spatial distribution of KC (magenta) and single/pair/trios (light blue) in two consecutive 3D-reconstructed slices (*n* = 3 mice). The neurogenic area and its core used to quantify cell densities in Figures 6L and 6N-SP are depicted as black lines (see also Method details). Quantifications in Figures 6K, 6L and 6N-6P were taken from these distributions, also considering the Z dimension. Scale: (C-C’) 15 µm; (I) 250 µm.

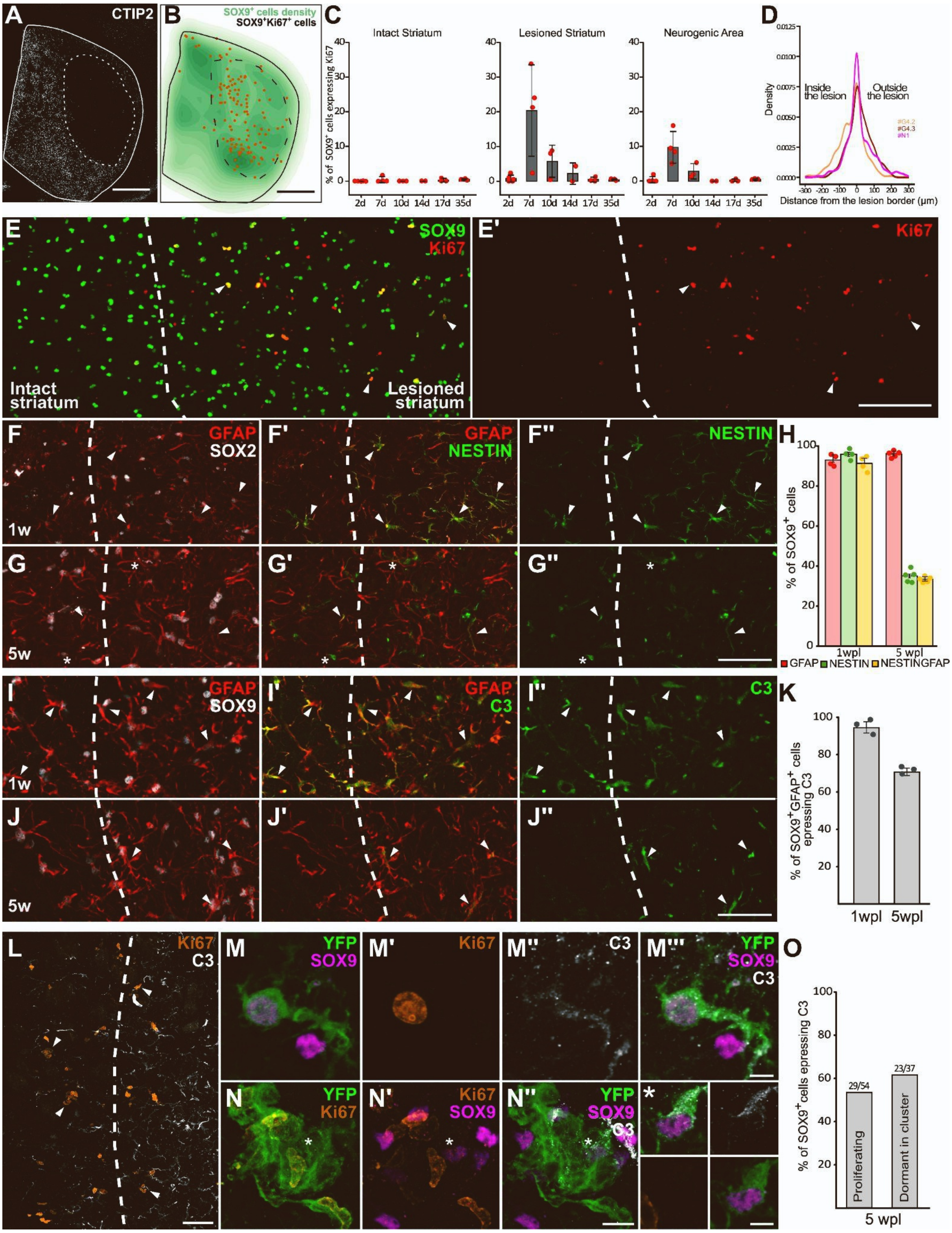

Figure S7. STR AS reactivity

(A,B) A couple of subsequent sections at the level of the rostral part of the lesion in a specimen at 1 wpl showing in B the density of SOX9⁺ cells (green) and the position of SOX9⁺Ki67⁺ proliferating ASs (orange dots). SOX9 and Ki67⁺ cells were counted automatically after segmenting them with Ilastik. The lesion border was drawn after

aligning with the immediately adjacent rostral section (A), labeled with CTIP2 (white). Note how the lesion border is already sharp at this stage. (C) Quantification of the fraction of ASs proliferating inside and outside the lesion or in the neurogenic area, which lies in between. For this analysis cells were counted manually in a section at the level of the central part of the lesion. The neurogenic area at 1 wpl was drawn as a 200-300 μm band centered on the lesion border. (D) 3D distances of neurogenic foci relative to the lesion border in the entirely reconstructed specimens #G4.2, #G4.3 and #N1 (Figures 1 and S1; Video S1). Note that these structures are relatively homogeneously distributed around the border. (E,E') Confocal stack spanning the entire section acquired around the lesion border from the section in (B) labeled for SOX9 (green) and Ki67 (red). Double labeled cells are located mostly inside the lesion. (F-G'') Confocal stack spanning 5 μm thickness of sections labeled for GFAP (red), NESTIN (green) and the AS marker SOX2 (white) taken at 1wpl and 5wpl. Arrowheads indicate double labeled cells, NESTIN also labels non-astrocytic cells, mostly within blood vessels (asterisk). (H) Quantification of the fraction of $SOX2^+GFAP^+$, $SOX2^+NESTIN^+$ and co-labelled cells in the neurogenic areas at 1wpl and 5wpl. (I-J'') Confocal stack spanning 5 μm thickness of sections labeled for GFAP (red), C3 (green) and SOX9 (white) taken at 1wpl and 5wpl. Arrowheads indicate double labeled cells. (K) Quantification of the fraction of $C3^+SOX9^+$ cells in the neurogenic area. Although C3 was originally proposed as a marker of neurotoxic ASs (Liddelow et al. 2017), it shows a delayed upregulation also in conditions that are not expected to stimulate this state such as 7 days after stroke (Zamanian et al. 2012) or in mature border ASs after spinal cord injury and it is physiologically expressed in the glia limitans (O'Shea et al. 2024). (L) Low magnification of the neurogenic area labeled for C3 and Ki67, arrowheads indicate KCs. (M) Proliferating AS in the neurogenic area labeled with Ki67, SOX9, and YFP in a *Glast*$^{CreERT2/+}$x*R26R*-YFP mice 14 days after tamoxifen injection. (N) Recombined KC containing a dormant $YFP^+SOX9^+Ki67^-$ AS (asterisk). (O) Fraction of C3 expression among $SOX9^+$ cells also expressing $Ki67^+$ in the neurogenic area (proliferating ASs) or $YFP^+SOX9^+Ki67^-$ directly associated with a YFP^+ KC (Dormant in Cluster). Data in ©, (H) and (K) are expressed as mean ± SD; dots indicate individual specimens. Scale: (A,B) 500 μm; (E,E') 150 μm; (F-G'') 50 μm; (I-J'') 50 μm; (L) 50 μm; (M-M'''') 5 μm; (N-N'') 10 μm; (*) 5 μm.

Supplemental experimental procedures

Histology

Animals were anesthetised with a ketamine (100 mg/kg) and xylazine (33 mg/kg) solution and perfused with a solution of 4% paraformaldehyde (PFA, Sigma-Aldrich) and 2% picric acid (AnalytiCals) in 0.1 M sodium phosphate buffer (PB) pH 7.4. Brains were then post-fixed for 5 hours, cryoprotected in 30% sucrose (Sigma-Aldrich) in 0.1M PB pH 7.4, embedded at −80°C in Killik/OCT (Bio-Optica), and cryostat sectioned in series of 50 µm-thick coronal sections.

3D reconstructions

Confocal microscopy serial section 3D reconstructions were performed by modifying a previous method (Luzzati et al., 2011). All the following image processing and alignment procedures were performed with Fiji using a set of custom-made scripts available at the following link: https://github.com/bunbunet/FogliNato2024_3Drec. Whole-section epifluorescence acquisitions (Zeiss Axio scan) or cryotomography images served as a backbone anatomical reference for the 3D reconstructions of high resolution confocal acquisitions.

Low resolution whole sections registration

Epifluorescence images were extracted from the original Carl Zeiss Image (.czi) file format using the script *"Export tif from czi.ijm"*. This script allows saving as .tif files the images of each section splitted by channel, assigning a prefix with the channel name and the desired LUTs. Additionally, it includes a "zN" tag codifying the position of each section relative to the entire brain, allowing a straightforward image alignment (see below).
Epifluorescence images were then imported in the Fiji plugin TrakEM2 (Cardona et al., 2012) using the script *"Import patches z names.py"* which imports the images into the appropriate layer based on their "zN" tag. The script also scales each image based on its pixel size to reach a final resolution of 1 µm/pixel. Thus, all the images that are imported with this script can be easily overlaid even though they were acquired at different resolutions.
Sequential sections were manually aligned with a landmark-based registration method to obtain a registration of all the sections that will work as the anatomical reference of the 3D reconstruction; we only used rigid transformations, i.e. translation and rotation.
To ease this initial alignment phase, in some specimens, we performed cryotomography imaging during the cryostat cutting procedure. Briefly, images were acquired with a customised system composed of a Raspberry Pi camera (V2 8MP) with additional 16mm M12 objective mounted through a customised support on the cryostat anti-roll glass, which was controlled through the script *"CRIOpi Capture.py"*. Images were then calibrated and processed using the script *"CRIOpi Processing.ijm"*

and imported as the first images in the TrakEM2 project using the same importing script. In those cases, epifluorescence images were simply overlaid with the cryotomography images and minimal adjustments to the overall image alignment were needed.

Confocal high-resolution images

The multi-channel z-stacks confocal acquisitions of the striatum (“z-stacks”) were named including the same “zN” tags of corresponding epifluorescence/cryotomography images. The z-stacks were initially batch-converted from the Leica image file (.lif) format into .tif files with the script *“Export tif from lif.ijm”*. Then, the z-stacks of each specimen were processed separately using the script *“Split tif to MAX and Sequence.ijm”* to obtain: i) the maximal intensity projections (“MAX projections”) of each channel and section; ii) the sequence of the single focal planes for each channel and section (“sequences”); iii) a .csv file containing the information for importing the sequences (see below). Similarly to what is done with the epifluorescence images, the script allows to apply the desired LUT to each channel and adds a prefix with the marker name (e.g. “Ki67^{-}”).
Then, the MAX projections of each confocal section were imported using the same importing script and manually overlaid to the corresponding epifluorescence images. Finally, the sequences of each section were imported at the right positions with the “import from text file” TrakEM2 function, using the .csv file previously generated by the script “*Split tif to MAX and Sequence.ijm”*. As a last step, the same transformation previously applied on each MAX projection was applied to its corresponding sequences to register all the z-stacks.
For each layer in TrakEM2 are now available separate image files for each channel that could be visualised as a merged image using the script *“Set patch composite mode.py”*. For inspection and manual counting, the script *“Set visibility toggle channels.py"* enables the use of customizable keyboard shortcuts for changing the visibility of each channel.

Annotations, region drawing and calibration

The KCs were manually annotated as TrakEM2 “ball objects”. The areas of the dorsal striatum were manually drawn according to the Allen Brain Atlas subdivisions. The lesioned areas were manually drawn based on GFAP expression. To correct the section shrinking along the z-axis and adjust the voxel size accordingly, the z-spacing of each focal plane was calculated by dividing the total depth of the reconstructed volume, according to cryostat sectioning, by the total number of focal planes. Of note, this is a fundamental requirement for obtaining proper distance measurements and performing 3D spatial analyses.

3D model alignment and visualisation in Blender

Surface areas drawn in TrakEM2 of the striatum, lesioned area and lateral ventricle, were exported as .obj files from TrakEM2 using the script *“Export Arealists.py”* and

imported in Blender (v2.79) with the script "*Blender_Import Surfaces and Reference points.py*". KC coordinates were exported using the script *"Export Balls.py"* or *"Export Balls Confetti.py"* and imported in Blender as spheres using the script "*Blender_Import_Coordinates_as_Balls.py*". Each specimen was imported in a different layer, after assigning materials, renderings were performed exclusively as orthographic views. All surfaces have been smoothed with a *smooth* modifier and additional imperfections were manually smoothed in sculpt mode. Reconstructions of the striatal projection domains of the anterior cingulate cortex (ACA) and the primary somatosensory areas(SSp) were obtained by aligning and manually drawing in TrakEM2 the segmentations of anterograde tracers injections obtained from (http://www.mouseconnectome.org/CorticalMap/page/map/5)(Hintiryan et al., 2016) and overlaid to the Allen reference atlas CCF V3. Reference samples were the SW110323-03 and SW110321-04 for ACA and SW120525-02, SW110418-01, SW110516-02, SW110418-02, SW110419-02 for SSp. Surfaces were imported in Blender as described. The registration of the different specimens to a common reference space has been performed manually based on the overall 3D shape of the striatum.

Data visualisation

All the graphs were generated with ggplot2 (Wickham, 2016), except for the G function analyses (Figures 2G and S3A-B) whose plots were produced with a dedicated function of the spatstat package (Baddeley et al., 2015).
The relative distributions reported in Figures 1F-1I, 6C, S4G and S7D actually represent the kernel density estimates of such distributions: the area under each curve is 1 and represents 100% of the observations. Although the term "relative" is not completely correct from a mathematical point of view, it accurately describes what is shown in the graph in a more understandable manner.
The volume rendering of the KC densities reported in Figures 1C-1E, S1A and S1B were obtained by computing 3D kernel density estimates of the KC in each specimen, by using the R package ks (Duong, 2007). Increasing KC density is shown with three objects of different colours: transparent (white boundary), yellow and orange. The obtained 3D objects were visualised and exported as .obj files with the R package rgl (Murdoch and Adler, 2023).

STATISTICAL ANALYSIS

Cellular composition of KCs at 5wpl

The cellular composition of KC at 5 wpl (Figure 2) was analyzed on entirely reconstructed KCs (*n* = 430) from 3-5 consecutive 3D reconstructed sections per mouse (*n* = 8 mice). KCed cells were manually counted using the MultiPoint tool in Fiji. As previously described (Nato et al., 2015), KCs were defined as groups of at least 4 Ki67^{+} cells showing direct contact among their cell bodies. Of note, due to this

stringent criterion used to define clustered cells, when prNBs were sparse in KCs of pmNBs not showing direct contact among their cell bodies that KC was excluded from the analysis. As a consequence, our analyses did not include the oldest KC population in which the proliferative potential is fading off (see also Supplementary text 1, section 4.2).

Time course analyses of KC distribution, number, cellular composition and turnover

Time course analyses of KC (Figures 3 and S4) were performed on three non-consecutive 400 µm-spaced sections for each specimen. It is to be noted that 63±9% of KCs were partially included in the analysed sections (Figure S4B). However, the fraction of incomplete KCs was constant among specimens (*One-way ANOVA*, $F_{(3,9)} = 1.53, p = 0.271$).

Clonal analyses of striatal astrocyte progeny

To visualize different reporters, we used anti-GFP antibody to recognize GFP, YFP, and CFP and anti-RFP antibody for RFP. GFP^+ cells were discriminated according to the localization of the green staining: nuclear (corresponding to "nGFP"), cytoplasmic (corresponding to "cYFP"), or membrane-associated (corresponding to "CFP").
For each Confetti specimen (Figures 4 and S5), all the consecutive sections containing striatal newborn cells were 3D reconstructed as described in the 3D reconstruction section (#C1: *n* = 26 slices; #C2: *n* = 24 slices; #C3: *n* = 32 slices; #C4: *n* = 29 slices). The spatial localization and the type of each KC were manually annotated in TrakEM2, allowing to map the KCs containing at least 1 Confetti $Reporter^+$ cell in 3D space. KC coordinates were exported from TrakEM2 using the script *"EXPORT_Balls_3.3.py"* for further spatial statistical analyses.
The cellular composition and reporter expression of these Confetti KCs was manually evaluated on 3D reconstructions from high resolution confocal acquisitions (see the "Confocal imaging" section). The same stringent criterion used to define clustered cells was applied for considering dormant astrocytes as part of the KC - and thus clonally related to the other cells it gave rise to. The same criteria were used for the quantification of clonally-related dormant astrocytes in subsequent fate-mapping analyses with *Glast*$^{CreERT2/+}$x*R26R*-YFP mice.
Striatal astrocytes recombination efficiency was measured as the fraction of $GFAP^+SOX9^+$ cells expressing each Confetti reporter (Figures 4 and S5), in four fields of view for each specimen.

Genetic fate-mapping analysis of striatal astrocytes activation and KC maturation

Fractions of YFP^+ STR KCs were counted over the entire thickness of 3 consecutive 3D reconstructed sections (Figure 5). The cellular composition of KC containing at least 1 YFP^+ cell was evaluated only for KCs entirely included in the volume (Figures

5 and 6). For the T-4 animals and two of the four T-7 animals that add less than 9 KCs within the first three sections, three or four additional slices were analysed.

Expression of ASCL1 and SOX9 in striatal $Ki67^+$ cells

$Ki67^+$ cells organized as single cells, pairs, trios or KCs were analysed on two consecutive reconstructed slices for each mouse (*n* = 3; Figures 6 and S6). We distinguished between $SOX9^{High}$ cells expressing this transcription factor at a similar level as the non-proliferating parenchymal astrocytes and $SOX9^{Low}$ cells expressing it at barely detectable levels (Figures 6F-6I).

Estimate of astrocyte activation rate

To estimate striatal astrocyte activation rate within the neurogenic area we segmented the $SOX9^+$ nuclei using Ilastik (Berg et al., 2019). Briefly, we trained a pixel classification model recognizing the nuclei centres, subsequently applied a watershed separation of individual objects using hysteresis thresholding and further trained an object classification model to recognise nuclei that were not separated, almost only couples of nuclei, or that were splitted, never more than in two pieces. The total number of nuclei was thus obtained by adding the number of individual nuclei, unsplitted nuclei*2 and splitted objects/2. The number of astrocytes and active $Ki67^+$ structures were counted over the neurogenic area and its core, using the R packages spatstat (Baddeley et al., 2015) and RImageJROI (https://github.com/davidcsterratt/RImageJROI). The neurogenic area and core were obtained by computing 3D kernel density estimates of the KC in each specimen with the R package ks (Duong, 2007), and included respectively the 95% and 25% of KCs with increasing density (see also Figure S6I).
Due to the extremely high density of $SOX9^+$ cells in the SVZ, quantifications in this region were performed manually, by counting the $SOX9^+Ki67^-$, the $SOX9^+Ki67^+$ and the $SOX9^-Ki67^+$ cells in the lateral wall of 3 SVZ from healthy mice. All counted objects, in both striatum and SVZ were counted stereologically by excluding those that touched one of the section surfaces.

Analysis of astrocyte reactivity

Astrocyte proliferation was assessed by quantifying the percentage of $SOX9^+$ cells, included in a focal plane, that expressed the endogenous marker of proliferation Ki67 (2dpl: *n* = 5; 7dpl: *n* = 4; 14dpl: *n* = 2; 17dpl: *n* = 3; 35dpl *n* = 3). The border of the lesion was drawn by registering the sections with the immediately adjacent rostral section labeled with CTIP2. For 7dpl specimens, an entire series of sections was automatically segmented for SOX9 and Ki67 as described in the previous section. One representative section of this analysis is shown in Figure S7B. The results were very similar to those obtained by the manual count revealing a rather constant level of astrocytes proliferation along the rostro-caudal axis of the lesion (data not shown). The number of GFAP and C3 labeled cells was counted on one section per

specimen (n=3) in the neurogenic area. For 1 wpl specimens, where no KCs are present, the counted area spanned a 2-300 µm band of tissue centered on the lesion border. Proliferating KCs and dormant astrocytes associated with YFP^+ KCs have been counted on 8 50 µm-thick sections from 4 *Glast*$^{CreERT2/+}$x*R26R*-YFP mice 14 days after tamoxifen injection.

Statistical tests

The results of all the analyses are reported in Table S1. We preferred to not overload the figures with the statistics to allow a better visualization of the data. We reported in the main text only the p-values of the comparisons we specifically cited there.
All analyses were performed using R Statistical Software (R Core Team 2021).

Means comparisons

To select the proper statistical test we always checked if the data were normally distributed, using the Shapiro–Wilk test, and homoscedastic, using Levene's test. Data that fulfilled those requirements were compared with the Test-t in the case of two groups or with the ANOVA in the case of three or more groups. ANOVA analyses that returned significant F values were followed by Tukey's post hoc test. In the case of non-normally distributed and/or non-homoscedastic data, we used the nonparametric alternatives: the Mann–Whitney U test and the Kruskal-Wallis test to compare two, or more than two groups, respectively. Significant Kruskal-Wallis tests were followed by Dunn's test for multiple comparisons (p-values were adjusted according to the Benjamini-Hochberg method). In the case of paired samples, the Wilcoxon signed-rank test was used instead of the Mann-Whitney U test.

Comparison of proportions

Fisher's Exact Test was used to compare proportions among count data with binary outcomes (e.g. fraction of KC associated or not associated with pmNBs). We use this test instead of the more conventional Chi-square test as it is more suitable to compare groups with low numbers of observations. For comparisons among more than two groups, significant Fisher's Exact Tests were followed by Pairwise Fisher's Exact Tests in which p-values were adjusted according to the Holm-Sidak method.

Comparison of frequency distributions

The Kolmogorov-Smirnov test was used to compare two distributions, while the Anderson-Darling k-sample test to compare three or more distributions.

Spatial statistics (point pattern analysis)

Evaluation of KC distribution

To assess if KCs distribute according to specific spatial patterns we used the G(r) function (Baddeley et al., 2015). This summary function represents the cumulative

frequency distribution of the first-order nearest neighbor distances (*r*) and it is one of the simplest techniques to find clustering or dispersion in a point pattern. Although a 3D version of this function is implemented in spatstat, we preferred to use its 2D counterpart as it allows, unlike the 3D version, the use of irregular polygonal areas describing the region from which the point pattern was retrieved. As shown in Figures 1 and S1, the striata and neurogenic areas are highly irregular and cannot be approximated to a parallelepiped. Further, since the analyses were performed on a few consecutive sections, KC dispersion was much greater on the X and Y axes than in Z, suggesting the reliability of a 2D projection analysis.
The G function calculated from the data ($G_{(obs)}(r)$) is visually inspected and compared to the theoretical one obtained from a homogeneous Poisson process ($G_{(theo,H0)}$) that represents the null hypothesis (*H0*) of complete spatial randomness (CSR). If $G_{(obs)}(r)$ lies at the left or at the right of $G_{(theo,H0)}(r)$ it suggests clustering or dispersion, respectively. Yet, to avoid false-positive results, each $G_{(obs)}(r)$ was compared with 999 Monte Carlo simulations of $G_{(theo,H0)}(r)$, and the graphs in Figures 2G and S3A-S3B report the mean of these simulations $G_{(mean,H0)}(r)$ and the simulation envelopes. For statistical comparisons (Figures S3A-S3B), we constructed simultaneous envelopes having a constant width around $G_{(mean,H0)}(r)$. The width is defined by finding the most extreme deviation, at any value of *r*, of the 999 simulated $G_{(theo,H0)}(r)$ from $G_{(mean,H0)}(r)$. Thus, the visual interpretation of the graphs coincides with the results of Maximum Absolute Deviation Tests (Baddeley et al., 2015). We rejected the null hypothesis of CSR if $G_{(obs)}(r)$ felt outside of the envelope at any value of *r*. As a complementary strategy to compare the experimental distributions with the simulations, we used the more powerful Diggle-Cressie-Loosmore-Ford test (Baddeley et al., 2015), that, instead of the maximum absolute deviation, uses the integrated squared deviation between $G_{(obs)}(r)$ and $G_{(theo,H0)}(r)$ over the range of measured *r*. For representative purposes, in Figure 2G we constructed pointwise envelopes instead of simultaneous ones, as we thought they were simpler to visually interpret (e.g $G_{(theo,H0)}(r) > 0$ at every value of *r*). However, we did not use such envelopes for statistical purposes as they would be more suitable for testing if clustering/dispersion exists at specific values of *r*, which was not what we intended to test here (Baddeley et al., 2015).
To perform these analyses, the R packages spatstat (Baddeley et al., 2015) and RImageJROI (https://github.com/davidcsterratt/RImageJROI) were used. The pooled p-values shown in the main text were obtained with the R package poolr (Cinar and Viechtbauer, 2022) by using Fisher's method on the p-values reported in Figures S3A-S3B.

Global spatial autocorrelation

To analyse the 3D distribution of KC features (cellular composition in Figure 2 and Confetti reporter expression in Figure 4I) we used Moran's I index of global spatial autocorrelation(Moran, 1950). It's a widely used technique in spatial econometrics(Anselin and Rey) that, similarly to a correlation coefficient, provides an estimate of how closer objects are more similar than distant ones. Thus, it's a

powerful technique to search for spatial clustering of similar objects. It bears the great advantages of being a “distance-based” metric. So, unlike other summary functions such as the G function, there’s no need to specify the study area/volume from which the point pattern was retrieved. Further, no edge correction methods are needed, avoiding the possible introduction of biases by the selection of specific methods or areas. This greatly helps in assessing the distribution of objects embedded in highly irregular and variable environments like the lesioned striata.

A positive Moran’s I index suggests clustering of similar objects, while a negative one is indicative of homogeneous dispersion. Yet, the Index value depends on the number of data points and the bigger the sample the more it approaches 0, which represents the absence of spatial autocorrelation. Thus, the sole examination of the Moran’s I Index is difficult to interpret. To understand if the observed data distribution was significantly different from complete spatial randomness, we performed a permutation test based on Monte Carlo simulations. The simulations randomly permuted the KC features over the same point pattern distribution and every time the resulting Moran’s I Index was measured. Finally, a p-value was calculated based on the proportion of simulations giving a statistic that is as or more extreme than the observed data. To perform this analysis, the R package ade4 (Dray and Dufour, 2007) was used.

Key resources tableSOX

REAGENT or RESOURCE	SOURCE	IDENTIFIER
Antibodies		
Rat monoclonal anti-BrdU (clone BU1/75 (ICR1)) (1:1500)	AbD Serotec	Cat#OBT0030CX; RRID:AB_609566
Rabbit anti-Ki67 (1:1000)	Novocastra	Cat#NCL-Ki67p; RRID:AB_442102
Rabbit polyclonal anti-Ki67 (1:1500)	Abcam	Cat#AB15580; RRID:
Rabbit anti-Ki67 (1:750)	Thermo Fisher Scientific	Cat#MA5-14520; RRID:AB_10979488
Goat polyclonal anti-DCX (1:2000)	Santa Cruz Biotechnology	Cat#Sc-8066; RRID:AB_2088494
Guinea Pig polyclonal anti-DCX (1:1500)	Millipore	Cat#AB2253; RRID:AB_1586992
Goat anti-SOX9 (1:1200)	R and D Systems	Cat# AF3075; RRID:AB_2194160
Chicken polyclonal anti-GFP (1:1000)	Aves labs	Cat#GFP-1020; RRID:AB_10000240
Rabbit polyclonal anti-RFP (1:1000)	Rockland	Cat# 600-401-379, RRID:AB_2209751
Rat monoclonal anti-CTIP2 (clone 25B6) (1:750)	Abcam	Cat#ab18465, RRID:AB_2064130

Mouse monoclonal anti-NEUN (1:1000)	Chemicon	Cat#MAB377; RRID:AB_2298772
Mouse anti-GFAP (1:1000)	Millipore	Cat#MAB360; RRID:AB_11212597
Mouse monoclonal anti-ASCL1 (1:100)	BD PharMingen	Cat#556604; RRID:AB_396479
Rabbit anti-IBA1 (1:1000)	FUJIFILM Wako Shibayagi	Cat# 019-19741; RRID:AB_839504
Rabbit anti-S100β (1:10000)	Swant	Cat#37A; RRID:AB_2315305
Goat polyclonal anti-SOX10 (1:750)	Santa Cruz Biotechnology	Cat#Sc-17342; RRID:AB_2195374
Rat polyclonal anti-C3 (1:250)	Abcam	Cat# ab11862; RRID:AB_2066623
Rabbit polyclonal anti-C3 (1:250)	Dako	Cat# A006302, RRID:AB_578478
Rat monoclonal anti-SOX2 (1:1000)	Thermo Fisher Scientific	Cat# 14-9811-82; RRID:AB_11219471
Goat polyclonal anti-NESTIN (C-20) (1:1000)	Santa Cruz Biotechnology	Cat# sc-21247; RRID:AB_650014
Cy3 AffiniPure Donkey anti-Rabbit IgG (1:800)	Jackson ImmunoResearch	Cat#711-165-152; RRID:AB_2307443
Cy3 AffiniPure Donkey anti-Mouse IgG (1:800)	Jackson ImmunoResearch	Cat#715-165-151; RRID:AB_2315777

Cy3 AffiniPure Donkey anti-Goat IgG (1:800)	Jackson ImmunoResearch	Cat#705-165-147; RRID:AB_2307351
Alexa Fluor 488 AffiniPure Donkey anti-Rat IgG (1:400)	Jackson ImmunoResearch	Cat#712-545-153; RRID:AB_2340684
Alexa Fluor 488 AffiniPure Donkey anti-Chicken IgY (1:400)	Jackson ImmunoResearch	Cat#703-545-155; RRID: AB_2340375
Alexa Fluor 488 AffiniPure Donkey anti-Mouse IgG (1:8400)	Jackson ImmunoResearch	Cat#715-545-151; RRID:AB_2341099
Alexa Fluor 647 AffiniPure Donkey anti-Rabbit IgG (1:400)	Jackson ImmunoResearch	Cat#711-605-152; RRID:AB_2492288
Alexa Fluor 647 AffiniPure Donkey anti-Goat IgG (1:800)	Jackson ImmunoResearch	Cat#705-605-147; RRID:AB_2340437
DyLight405 AffiniPure Donkey anti-Guinea Pig IgG (1:400)	Jackson ImmunoResearch	Cat#706-475-148; RRID:AB_2340470
DyLight405 AffiniPure Donkey anti-Rat Pig IgG (1:400)	Jackson ImmunoResearch	Cat#712-475-153; RRID:AB_2340681
Alexa Fluor 594 AffiniPure Donkey anti-Goat IgG (1:600)	Jackson ImmunoResearch	Cat#705-585-003; RRID:AB_2340432
Horse Anti-Goat IgG, Biotinylated (1:100)	VectorLabs	Cat#BA-8000; RRID:AB_2336140
AMCA-avidinD (1:100)	VectorLabs	Cat# A-2008, RRID:AB_2336102

Fab fragment Donkey anti-Rabbit IgG (1:100)	Jackson ImmunoResearch	Cat#711-007-003; RRID:AB_2340587
Chemicals, peptides, and recombinant proteins		
Tamoxifen	Sigma-Aldrich	Cat#T5648
Corn oil	Sigma-Aldrich	Cat#C8267
5-bromo-2-deoxyuridine (BrdU)	Sigma-Aldrich	Cat#B5002
Quinolinic acid	Sigma-Aldrich	Cat#Q104
Experimental models: Organisms/strains		
Slc1a3$^{tm1(cre/ERT2)Mgoe}$ (*Glast*^{CreERT2})		MGI:5466676
Gt(ROSA)26Sor$^{tm1(EYFP)Cos}$ (*R26R*-YFP)		MGI:2449038
Gt(ROSA)26Sor$^{tm1(CAG-Brainbow2.1)Cle}$/J (*R26R*-Confetti)		MGI:5317215
C57BL/6JOlaHsd		MGI:2164189
Software and algorithms		
LAS X	Leica Microsystems	https://www.leica-microsystems.com/products/microscope-software/p/leica-las-x-ls/
Fiji	Fiji	https://fiji.sc,<br><br>RRID:SCR_002285

TrakEM2	(Cardona et al 2012)	https://imagej.net/plugins/trakem2/
Imaris	Bitplane	v9.7.2, http://www.imaris.oxinst.com, RRID:SCR_007370
Blender	Blender Foundation	v2.79, https://www.blender.org/download/releases/2-79/
Ilastik	(Berg et al., 2019)	v1.4.0,<br><br>https://www.ilastik.org/
The R Project for Statistical Computing	(R Core Team 2021)	http://www.r-project.org/, RRID:SCR_001905
ggplot2	(Wickham, 2016)	v3.4.4
RImageROI	https://github.com/davidcsterratt/RImageJROI	v0.1.2
spatstat	(Baddeley et al 2015)	v3.0-7
ks	(Duong 2007)	v1.14.1
rgl	(Murdoch and Adler, 2023)	v1.2.1, https://github.com/dmurdoch/rgl
ade4	(Dray and Dufour, 2007)	v1.7-22
poolr	(Cinar and Viechtbauer, 2022)	v1.1-1

Supplemental table

Table S1. *Results of all statistical analyses presented in the study*

* Indicates that a post hoc test was performed, please see below

Graph	Statistical test	Result	P Value	Post-hoc
Fig. 1F	Linear Regression	AdjR^2 = 0.8895	9.04E-4	
Fig. 2D (Total cells)	Kruskal-Wallis Test	K-W chi squared = 212.4, df = 4	8.24E-45	*
Fig. 2D (TAPs)	Kruskal-Wallis Test	K-W chi squared = 120.5, df = 3	6.12E-26	*
Fig. 2D (prNBs)	Kruskal-Wallis Test	K-W chi squared = 125.4, df = 3	5.30E-27	*
Related to Fig. 2B (N of cells vs association with pmNBs)	Logistic Regression	AdjR^2 = 0.31	5.96E-21	
Related to Fig. 2B (% of prNBs vs association with pmNBs)	Logistic Regression	AdjR^2 = 0.44	2.19E-25	
Fig. 2H	Permutation Test using Monte Carlo Simulations	Moran's I coefficients	*	
Fig. S2F	Fisher's Exact Test		4.99E-4	*
Fig. 3E	One-Way ANOVA	F(4,13) = 1.98	0.157	
Fig. 3G	Mann–Whitney Test	Mann–Whitney U = 22678	6.907E-11	
Fig. 3H	Mann–Whitney Test	Mann–Whitney U = 22711	1.955E-11	
Fig. 3I	Fisher's Exact Test		2.932E-12	
Fig. 3J	Independent samples t-test	t(6.6704) = 1.0764	0.3191	
Fig. S4C (% of TAPs-only)	One-Way ANOVA	F(3,9) = 3.883	0.049	
Fig. S4C (% of TAPs+prNBs)	One-Way ANOVA	F(3,9) = 0.23	0.873	
Fig. S4C (% of prNBs-only)	One-Way ANOVA	F(3,9) = 1.824	0.213	
Fig. S4D (N of cells in TAPs-only)	Kruskal-Wallis Test	K-W chi squared = 2.5423, df = 3	0.4677	
Fig. S4D (N of cells in TAPs+prNBs)	Kruskal-Wallis Test	K-W chi squared = 2.2104, df = 3	0.5299	
Fig. S4D (N of cells in prNBs-only)	Kruskal-Wallis Test	K-W chi squared = 6.9651, df = 3	0.07302	
Fig. S4E	Independent samples t-test	t(7.0858) = -5.9575	0.0005403	
Fig. S4F (TAPs vs prNBs)	Paired samples t-test	t(3) = 3.4728	0.04026	
Fig. S4F (TAPs in TAPs-only vs TAPs in TAPs+prNBs)	Paired samples t-test	t(3) = -2.1131	0.125	
Fig. S4F (prNBs in TAPs+prNBs vs prNBs in prNBs-only)	Paired samples t-test	t(3) = 2.0584	0.1317	
Fig. S4G	Kolmogorov-Smirnov Test	D = 0.093482	0.247	
Fig. 4F	Linear Regression	AdjR^2 = 0.88	0.042	
Fig. 4H	Linear Regression	AdjR^2 = 0.53	0.004	
Fig. 4I	Monte Carlo Permutation Test	Moran's I coefficients	*	
Fig. S5E	One-Way ANOVA	F(2,9) = 1.571	0.26	
Fig. S5F	One-Way ANOVA	F(2,9) = 0.156	0.858	
Fig. S5G	One-Way ANOVA	F(2,9) = 0.834	0.512	
Fig. S5H	One-Way ANOVA	F(2,9) = 1.834	0.219	
Fig. S5K (TAPs-only)	Fisher's Exact Test		1	
Fig. S5K (TAPs+prNBs)	Fisher's Exact Test		0.454	
Fig. S5K (prNBs-only)	Fisher's Exact Test		0.301	
Fig. 5D	One-Way ANOVA	F(3,10) = 14.550	0.00056	*
Fig. 5E (TAPs-only)	One-Way ANOVA	F(3,10) = 4.486	0.03056	*
Fig. 5E (TAPs+prNBs)	One-Way ANOVA	F(3,10) = 7.520	0.00638	*
Fig. 5E (prNBs-only)	One-Way ANOVA	F(3,10) = 17.801	0.00025	*

Fig. 5J	Fisher’s Exact Test		6.428E-05	*
Fig. 6K	Mann–Whitney Test	Mann-Whitney U = 1161	0.01312	
Fig. 6N	One-Way ANOVA	F(2,6) = 68.52	7.38E-08	*
Fig. 6O	One-Way ANOVA	F(2,6) = 2.435	0.168	
Fig. 6P	One-Way ANOVA	F(2,6) = 0.73	0.52	
Fig. S6A (Total)	Kruskal-Wallis Test	K-W chi squared = 37.595, df = 3	3.443E-08	*
Fig. S6A (100%)	Kruskal-Wallis Test	K-W chi squared = 52.062, df = 3	2.905E-11	*
Fig. S6B (Total)	Kruskal-Wallis Test	K-W chi squared = 43.687, df = 3	1.759E-09	*
Fig. S6B (100%)	Kruskal-Wallis Test	K-W chi squared = 31.305, df = 3	7.334E-07	*
Fig. S6F (including the 5 orange)	Wilcoxon signed-rank Test	V = 63.5	0.0414	
Fig. S6F (excluding the 5 orange)	Wilcoxon signed-rank Test	V = 52	0.2512	
Fig. S6G (including the 5 orange)	Wilcoxon signed-rank Test	V = 12	0.03756	
Fig. S6G (excluding the 5 orange)	Wilcoxon signed-rank Test	V = 9	0.4469	
Fig. S6H (SOX9$^+$ASCL1$^-$ cells)	Fisher’s Exact Test		1.014E-11	*
Fig. S6H (SOX9$^+$ASCL1$^+$ cells)	Fisher’s Exact Test		<2.2E-16	*
Fig. S6H (SOX9$^-$ASCL1$^+$ cells)	Fisher’s Exact Test		4.492E-10	*
Fig. S7C (intact)	One-Way ANOVA	F(5,14) = 0.849	0.37361	
Fig. S7C (lesioned)	One-Way ANOVA	F(5,14) = 5.654	0.00466	*
Fig. S7C (neurogenic area)	One-Way ANOVA	F(5,14) = 10.1	0.00029	*
Fig. S7H (GFAP 1W vs 5W)	Independent samples t-test	t(4.49) = -1.90	0.12212	
Fig. S7H (NESTIN 1W vs 5W)	Independent samples t-test	t(6.9953) = 33.221	5.857E-09	
Fig. S7H (GFAP NESTIN 1W vs 5W)	Independent samples t-test	t(3.6308) = 29.9	1.758E-051	
Fig. S7K (C3 1W vs 5W)	Independent samples t-test	t(3.3483) = 8.7313	0.00204	

Post hoc analyses

**Figures 2D and S2F. Post hoc**	Figure 2D Dunn's Test for multiple comparisons			Figure S2F
Comparison	Total cells	TAPs	prNBs	Pairwise Fisher’s Exact Test
TAPs-only - TAPs+prNBs_Low	0.0412478	0.179955		0.0303
TAPs-only - TAPs+prNBs_Med	8.74E-13	0.064751		7.44E-09
TAPs-only - TAPs+prNBs_High	1.21E-44	9.51E-23		1.85E-38
TAPs-only - prNBs-only	7.18E-22			1.31E-41
TAPs+prNBs_Low - TAPs+prNBs_Med	0.1065108	0.034045	0.024782	0.542
TAPs+prNBs_Low - TAPs+prNBs_High	6.74E-05	1.43E-08	3.47E-12	0.000136
TAPs+prNBs_Low - prNBs-only	0.0265778		6.50E-11	1.64E-05
TAPs+prNBs_Med - TAPs+prNBs_High	7.84E-06	1.26E-10	1.72E-18	2.31E-09
TAPs+prNBs_Med - prNBs-only	0.2312437		5.97E-15	1.36E-11
TAPs+prNBs_High - prNBs-only	0.0001211		0.362679	0.542

**Figure 2H. Moran’s I analysis**	AnimalID	Moran's I Index	P value
Total cells	G14.3	-0.003	0.34
Total cells	G14.4	-0.058	0.821
Total cells	N2	-0.033	0.683
Total cells	N3	-0.005	0.422
Total cells	N1	0.013	0.388
% of prNBs	G14.3	-0.029	0.553
% of prNBs	G14.4	-0.085	0.516
% of prNBs	N2	-0.014	0.984
% of prNBs	N3	-0.019	0.727

% of prNBs	N1	0.001	0.724
Association w/ pmNB	G14.3	-0.038	0.247
Association w/ pmNB	G14.4	-0.057	0.929
Association w/ pmNB	N2	0.033	0.745
Association w/ pmNB	N3	-0.042	0.63
Association w/ pmNB	N1	0.034	0.222

**Figure S4C (TAPs-only). Post hoc**	Comparison	P value
	3wpl-4wpl	0.920
	3wpl-5wpl	0.807
	3wpl-8wpl	0.216
	4wpl-5wpl	0.996
	4wpl-8wpl	0.087
	5wpl-8wpl	0.047

**Figure 4I. Moran's I analysis**	Specimen	Moran's I Index	P value
	#C1	-0.005	0.622
	#C2	-0.025	0.649
	#C3	-0.005	0.991
	#C4	-0.002	0.519

**Figures 5D and 5E. Tukey Post hoc**	Total clusters	TAPs-only	TAPs+prNBs	prNBs-only
T-4 vs T-7	0.4186	0.9744	0.1374	0.7795
T-4 vs T-14	0.0021	0.3591	0.0092	0.0006
T-4 vs T-bQA	0.0016	0.0369	0.0098	0.0053
T-7 vs T-14	0.0150	0.5167	0.3027	0.0012
T-7 vs T-bQA	0.0096	0.0501	0.2642	0.0142
T-14 vs T-bQA	0.9383	0.3520	0.9949	0.5948

**Figure 5J. Pairwise Fisher's Exact Test**	P value
T-4 vs T-7	1
T-4 vs T-14	0.0039
T-4 vs REF	0.0033
T-7 vs T-14	0.00984
T-7 vs REF	0.00984
T-14 vs REF	1

**Figure 6N. Tukey Post hoc**	P value
N_Area vs N_Core	0.989
N_Area vs hSVZ	0.00013
N_Core vs hSVZ	0.00014

**Figures S6A-S6B. Dunn's Test or multiple comparisons**	Figure S6A (N of cells)		Figure S6B (% of prNBs)	
Comparison	Total	100%	Total	100%
T-4 vs T-7	0.9090	0.0288	0.0740	0.0611
T-4 vs T-14	0.0027	6.085E-05	7.069E-06	0.0006
T-4 vs REF	0.00001	4.558E-08	9.117E-09	1.621E-05
T-7 vs T-14	0.0055	0.0265	0.0267	0.0613
T-7 vs REF	0.0001	3.658E-05	0.0030	0.0022
T-14 vs REF	0.2486	0.0293	0.5487	0.2340

**Figure S6H. Pairwise Fisher's Exact Test**	$SOX9^{+}ASCL1^{-}$	$SOX9^{+}ASCL1^{+}$	$SOX9^{-}ASCL1^{+}$
Single VS Pair	0.116	0.284	0.556
Single VS Trio	0.116	1	0.237
Single VS Cluster	4.36E-07	2.8E-03	1.05E-03
Pair VS Trio	1	0.106	0.237
Pair VS Cluster	1.68E-04	1.11E-19	1.36E-06
Trio VS Cluster	6.08E-03	2.1E-04	0.237

**Figure S7C. Tukey Post hoc**	lesioned	neurogenic area
2d VS 7d	0.0049571	0.0003787
7d VS 10d	0.0856219	0.0158508
7d VS 14d	0.0511228	0.0028811
7d VS 17d	0.0122696	0.0011219
7d VS 35d	0.0122024	0.0014631
2d VS 10d	0.8896538	0.7136573
2d VS 14d	0.9998048	0.9999466
2d VS 17d	0.9999998	0.9999979
2d VS 35d	0.9999998	0.9999999
10d VS 14d	0.9884671	0.7697894
10d VS 17d	0.9095828	0.7457391
10d VS 35d	0.9086443	0.8182281
14d VS 17d	0.9996753	0.9999983
14d VS 35d	0.9996613	0.9999001
17d VS 35d	1.0000000	0.9999902

Supplemental references

Anselin, L., and Rey, S.J. Modern spatial econometrics in practice: AGuideto Geo Da, Geo DaSpace and PySAL.

Baddeley, A., Rubak, E., and Turner, R. (2015). Spatial Point Patterns: Methodology and Applications with R (CRC Press).

Berg, S., Kutra, D., Kroeger, T., Straehle, C.N., Kausler, B.X., Haubold, C., Schiegg, M., Ales, J., Beier, T., Rudy, M., et al. (2019). ilastik: interactive machine learning for (bio)image analysis. Nat. Methods *16*, 1226–1232. .

Cardona, A., Saalfeld, S., Schindelin, J., Arganda-Carreras, I., Preibisch, S., Longair, M., Tomancak, P., Hartenstein, V., and Douglas, R.J. (2012). TrakEM2 software for neural circuit reconstruction. PLoS One *7*, e38011. .

Cinar, O., and Viechtbauer, W. (2022). The poolr Package for Combining Independent and Dependent p Values. J. Stat. Softw. *101*, 1–42. .

Dray, S., and Dufour, A.-B. (2007). The ade4 Package: Implementing the Duality Diagram for Ecologists. J. Stat. Softw. *22*, 1–20. .

Duong, T. (2007). ks: Kernel Density Estimation and Kernel Discriminant Analysis for Multivariate Data in R. J. Stat. Softw. *21*, 1–16. .

Hintiryan, H., Foster, N.N., Bowman, I., Bay, M., Song, M.Y., Gou, L., Yamashita, S., Bienkowski, M.S., Zingg, B., Zhu, M., et al. (2016). The mouse cortico-striatal projectome. Nat. Neurosci. *19*, 1100–1114. .

Luzzati, F., Fasolo, A., and Peretto, P. (2011). Combining Confocal Laser Scanning Microscopy with Serial Section Reconstruction in the Study of Adult Neurogenesis. Front. Neurosci. *5*, 70. .

Moran, P.A.P. (1950). Notes on continuous stochastic phenomena. Biometrika *37*, 17–23. .

Wickham, H. ggplot2 (Springer International Publishing).
