# Supplementary material for: Dynamic spatiotemporal activation of a pervasive neurogenic competence in striatal astrocytes supports continuous neurogenesis following injury
Source: Stem Cell Reports. 2024 Sep 19;19(10):1432–50. doi: 10.1016/j.stemcr.2024.08.006 (PMC11561465; doi:10.1016/j.stemcr.2024.08.006)
Supplement: Document S2. Article plus supplemental information [file mmc5.pdf]

Stem Cell Reports
Article
OPEN ACCESS

Dynamic spatiotemporal activation of a pervasive neurogenic competence in striatal astrocytes supports continuous neurogenesis following injury

Marco Fogli,[1,2,6] Giulia Nato,[1,3,6] Philip Greulich,[4,5] Jacopo Pinto,[2] Marta Ribodino,[1,3] Gregorio Valsania,[1,2] Paolo Peretto,[1,2] Annalisa Buffo,[1,3] and Federico Luzzati[1,2,7,*]
[1]Neuroscience Institute Cavalieri Ottolenghi, Orbassano (Turin), Italy
[2]Department of Life Sciences and System Biology, University of Turin, Turin, Italy
[3]Department of Neurosciences "Rita Levi Montalcini", University of Turin, Turin, Italy
[4]School of Mathematical Sciences, University of Southampton, Southampton, UK
[5]Institute for Life Sciences (IfLS), University of Southampton, Southampton, UK
[6]These authors contributed equally
[7]Lead contact
*Correspondence: federico.luzzati@unito.it
https://doi.org/10.1016/j.stemcr.2024.08.006

SUMMARY

Adult neural stem cells (NSCs) are conventionally regarded as rare cells restricted to two niches: the subventricular zone (SVZ) and the subgranular zone. Parenchymal astrocytes (ASs) can also contribute to neurogenesis after injury; however, the prevalence, distribution, and behavior of these latent NSCs remained elusive. To tackle these issues, we reconstructed the spatiotemporal pattern of striatal (STR) AS neurogenic activation after excitotoxic lesion in mice. Our results indicate that neurogenic potential is widespread among STR ASs but is focally activated at the lesion border, where it associates with different reactive AS subtypes. In this region, similarly to canonical niches, steady-state neurogenesis is ensured by the continuous stochastic activation of local ASs. Activated ASs quickly return to quiescence, while their progeny transiently expand following a stochastic behavior that features an acceleration in differentiation propensity. Notably, STR AS activation rate matches that of SVZ ASs indicating a comparable prevalence of NSC potential.

INTRODUCTION

Stem cells are typically rare cells confined to specialized anatomical regions, called niches, that ensure their maintenance and regulate their activity (Altshuler et al., 2023). In the adult mammalian brain, two such regions have been identified, the subventricular zone (SVZ) and the subgranular zone (SGZ), where astrocyte (AS)-like cells act as neural stem cells (NSCs) throughout life (Bond et al., 2015; Dray et al., 2021a; Obernier and Alvarez-Buylla, 2019). These cells are mostly quiescent (qNSC) but sporadically activate generating transit amplifying progenitors (TAPs) that further divide before differentiating into neuroblasts (NBs) (Pilz et al., 2018; Ponti et al., 2013). The stochastic activation of widespread qNSCs within the niche ensures continuous neuron production (Basak et al., 2018; Than-Trong et al., 2020). Outside these niches, the mature brain parenchyma has been traditionally considered non-permissive for neurogenesis (Götz et al., 2015). However, single-cell RNA sequencing (RNA-seq) revealed striking similarities between parenchymal ASs and qNSC (Llorens-Bobadilla et al., 2015). and all major elements maintaining stem cells in canonical niches are present in the parenchyma (Boda et al., 2017). Parenchymal ASs may thus represent NSCs in a deep quiescent state. According to this notion, during early postnatal development (Laywell et al., 2000) or after injuries inducing proliferative AS reactivity, subsets of parenchymal ASs can expand *in vitro* as neurospheres (Buffo et al., 2008; Sirko et al., 2013) and become transcriptionally similar to NSC primed for activation (Zamboni et al., 2020). Notably, in the mouse striatum (STR), some ASs move beyond the primed state and express their neurogenic capacity *in vivo*, after stroke or quinolinic acid (QA)-mediated excitotoxic lesion, supporting neurogenesis for several months (Magnusson et al., 2014; Nato et al., 2015; Thored et al., 2006). However, the prevalence, spatial distribution, and dynamics of these ectopic NSCs were not resolved. Consequently, how widespread is NSC potential among parenchymal ASs and to what extent the parenchyma is permissive for its maintenance and expression remain unclear. STR neurogenic ASs may simply represent a new rare NSC population. Accordingly, the mainstream view in the field still adheres to the concept of the anatomical restriction of NSCs potential (Urbán et al., 2019). To address these issues, we investigated the spatiotemporal dynamics of neurogenic activity and lineage progression of STR ASs after QA lesion.

To reach this goal, we built upon our previous demonstration that in this model new STR neurons originate exclusively from STR ASs through neurogenic foci scattered around the lesion border. These foci are made of cells expressing the proliferation marker Ki67 and organized in clusters (KCs). The KCs include a few putative ASs but mostly TAPs-like cells and proliferating NBs (prNBs) and are often associated with their early postmitotic NBs (pmNBs) (Nato et al., 2015).

 | © 2024 The Author(s).
Published by Elsevier Inc. on behalf of International Society for Stem Cell Research.
This is an open access article under the CC BY license (http://creativecommons.org/licenses/by/4.0/).

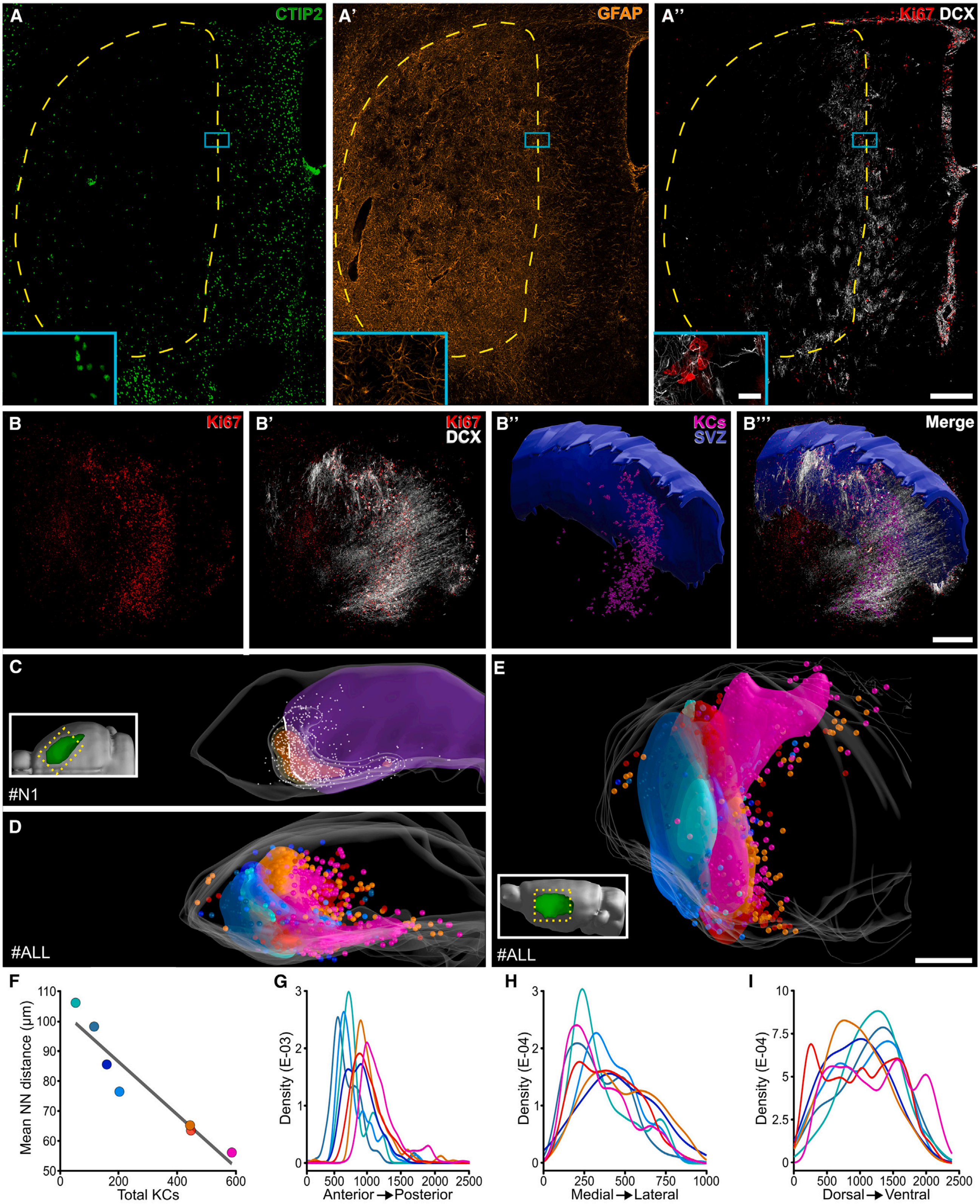

(legend on next page)

Now we demonstrate that these neurogenic foci are transient structures continuously generated by the stochastic activation of a widespread population of neurogenic ASs residing in a globally permissive environment. By clarifying the spatiotemporal dynamics of these atypical progenitors, we unveiled an unprecedented level of neurogenic competence among parenchymal ASs, comparable to that of canonical niches ASs.

RESULTS

Neurogenic foci organize in a 3D germinal matrix centered around the lesion border

To analyze the spatial distribution of the KCs, defined as groups of at least 4 cells in direct contact, we 3D reconstructed the STR of 7 specimens at 5 weeks post lesion (wpl). As previously described (Nato et al., 2015), QA lesions caused the loss of STR neurons in a large dorsolateral domain that was filled by densely arranged GFAP+ ASs (Figures 1A–1A′). KCs organized in a 3D germinal matrix centered around the rostro-medial part of the lesion border in both lesioned and spared tissue (Figures 1A″–1C, S1A, and S1B; Video S1).

The peak KC density was always far from the SVZ, in line with the STR origin of these structures (Figures 1C and S1A). The KC number varied greatly, ranging from 55 to 586 (mean ± SD = 288 ± 202), and correlated with their mean nearest neighbor distance (Figure 1F; Table S1, $p < 0.001$), indicating that stronger neurogenic responses resulted in increased foci density within similar areas. When all specimens were registered together, neurogenic areas collectively occupied most of the rostral and medial STR (Figures 1D, 1E, 1G–1I, S1A, and S1B; Video S1). In particular, the neurogenic foci were preferentially distributed in the medial STR (Figure 1H), an associative functional domain (Figures S1A′ and S1B′; Hintiryan et al., 2016). By contrast, these structures were more sparse in the lateral somato-motor STR and extremely rare in the caudal multi-modal domain (Figures S1C–S1E′).

In summary, STR neurogenic foci organize in a complex 3D germinal matrix whose spatial disposition is contingent on the lesion border. The potential of neurogenic foci induction is widespread in the STR with a higher probability in its medial domain.

Neurogenic foci exist along a continuum of maturation profiles

KCs have been described in different models of STR neurogenesis, but their individual cellular composition has never been resolved (Luzzati et al., 2006, 2011a; Magnusson et al., 2014; Nato et al., 2015). The size and fraction of TAPs and prNBs in these foci could (1) vary along a continuum of maturation profiles as in adult neurogenic niches or (2) could be more invariant as in stem cell systems, like the skin, where continuous activity at fixed locations maintains a stationary proportion of maturation stages (Altshuler et al., 2023). To distinguish between these models, here we evaluated the TAPs (DCX$^-$Ki67$^+$) and prNBs (DCX$^+$Ki67$^+$) content of 430 KCs 3D reconstructed from serial sections at 5 wpl ($n = 8$ mice; Figures 2A and S2A–S2E).

The KCs varied greatly in size, ranging from 4 to 38 cells (mean ± SD = 11.8 ± 7.6 cells), and in the proportion of TAPs and prNBs (Figures 2A and 2B). About half of the KCs were composed of only TAPs (TAPs-only KC) or only prNBs (prNBs-only KC) while the other half included a mix of the two cell types (TAPs+prNBs KC; Figures 2A–2C). We further subdivided TAPs+prNBs KCs into TAPs+prNBs_Low, Med, and High according to the prNB fraction (Figures 2A–2C). Both KC size and number of prNBs increased with the prNB fraction, progressing from TAPs-only to TAPs+prNBs_High KCs (Figure 2D; Table S1, *size:* $p < 0.001$; *prNBs*: $p < 0.001$). Conversely, TAPs exhibited an opposite trend (Figure 2D; Table S1, $p < 0.001$). The increase in prNB content and size further correlated with a higher probability of pmNBs (DCX$^+$Ki67$^-$) being part of the neurogenic foci (Figures 2B and S2F; Table S1, *percentage of prNBs:* $p < 0.001$; *size:* $p < 0.001$). Of note, in all the TAPs-only KCs, most of the cells expressed the TAP marker ASCL1 (Parras et al., 2004) but not the AS marker SOX9 (Cheng et al., 2009; Sun et al., 2017; Figures 2E and S2G–S2G′), confirming the neuronal commitment of these structures.

Figure 1. Neurogenic foci form a 3D germinal matrix

(A–A″) CTIP2-, GFAP-, Ki67-, and DCX-stained section. Dashed yellow line: lesioned area. Inset: high magnification of a KC.

(B–B‴) 3D reconstruction of STR Ki67 and DCX staining (specimen #N1; see also Video S1). SVZ is hidden. In (B″-B‴) segmented STR KCs are in magenta.

(C) Dorsal view of #N1. The lesion is in purple. White dashed line: the lesion border in the neurogenic area. Gray dots: KC (25 μm diameter). Increasing relative KC density is rendered as transparent, yellow, and orange volumes.

(D and E) Overlap of 7 specimens in a common coordinate frame. The KC (100 μm radius) and the volume rendering of KC density are colored by specimen.

(F) Correlation between the number of KC and mean nearest neighbor distance among them. Black line: linear regression.

(G–I) Relative KC distribution along the antero-posterior (G), medio-lateral (H), and dorsoventral (I) axis. Scale: (A) 200 μm; (inset A) 20 μm; (B–B‴), (C), (D), and (E) 500 μm.

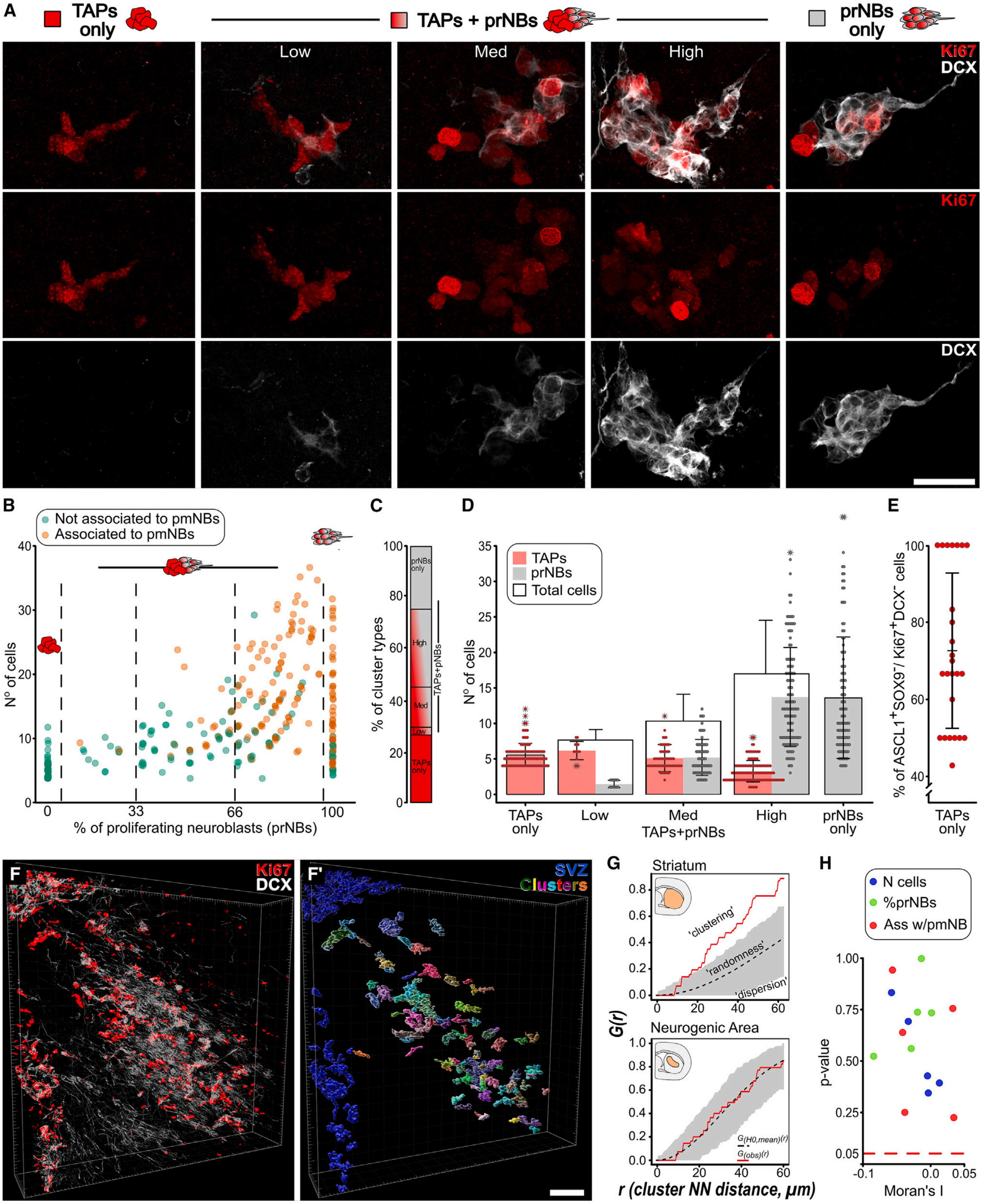

(legend on next page)

Neurogenic foci cellular composition thus varies along a continuous spectrum of transitions that may represent sequential stages of a common developmental process. KCs may be initially composed only of TAPs, progressively accumulate prNBs, generate pmNB, and ultimately deplete the progenitor pool. Alternatively, this variability may be contingent on spatial or temporal factors.

Neurogenic foci are spatially independent

STR neurogenic foci heterogeneity could result at least in part from regional differences in lineage progression. To verify this possibility we performed a spatial analysis of 318 3D reconstructed KCs at 5 wpl (n = 5 mice; Figures 2F–2F′; Video S2). As expected, spatial point pattern analyses detected significant clustering of KCs along the lesion border (Figures 2G and S3A; *pooled* $p < 0.001$). On the contrary, within the neurogenic area, KCs were randomly distributed (Figures 2G and S3B; *pooled* $p = 0.229$). We next employed a measure of global spatial autocorrelation, the Moran's I index, to understand if the KC size, prNB content, and association with pmNBs are distributed following specific spatial patterns (Figures S3C–S3C″). For all the specimens, the spatial distribution of these features did not deviate significantly from simulations of complete spatial randomness (Figure 2H; Table S1).

Hence, we conclude that KCs are randomly distributed in the neurogenic area and that their maturation profiles are spatially independent.

Neurogenic foci number, distribution, and cellular composition are stable after neurogenesis onset

To ascertain the temporal dynamics of STR neurogenesis, KC number, distribution, and composition were analyzed at 3, 4, 5, or 8 wpl. As observed at 5 wpl, KCs were always centered around the lesion border and their overall distribution did not vary in time (Figure S4A). Similarly, their total number remained stable over time (Figures 3B–3E and S4A; Table S1, $p = 0.157$). Of note, all the KC types described by the 3D reconstruction analysis (Figures 2A–2C) were identified in all specimens and time points and both their relative proportions and size were constant (Figures S4B–S4D; Table S1). These data show that, shortly after their appearance, between 2 and 3 wpl, the number, distribution, and composition of STR neurogenic foci reach a stable level that is maintained up to 8 wpl.

Neurogenic foci undergo continuous turnover

The KCs stable heterogeneity suggests they are turned over. To assess KC turnover rates, we first established a saturating bromodeoxyuridine (BrdU) protocol. Two BrdU injections over 8 h ("B-8h" group) at 5 wpl labeled 97% ± 2% of the KCs (Figures 3A and 3F) and 74% ± 6% of their cells (Figures 3A′ and S4E) indicating that virtually all KCs are made of actively dividing cells. As in the SVZ (Ponti et al., 2013), TAPs had a slightly higher proliferation rate than prNBs (Figure S4F; Table S1, $p = 0.040$), independent of the KC maturation profile (Figure S4F; Table S1, *TAPs:* $p = 0.125$; *prNBs:* $p = 0.132$).

We then analyzed the fraction of BrdU$^+$ KCs after a four-day chase at 3, 4, 5, or 8 wpl ("B-4d" groups; Figures 3B–3D). At all time points, about 40% of the KCs lacked BrdU$^+$ cells (BrdU$^-$ KCs, Figure 3F). Compared to BrdU$^+$ KCs, the BrdU$^-$ KCs exhibited smaller sizes, lower fractions of prNBs, and less association with pmNBs (Figures 3B′ and 3G–3I; Table S1, $p < 0.001$ *in each case*). The lack of BrdU$^+$ cells and lower maturation profile indicate that BrdU$^-$ KCs were established within four days after the BrdU injection. Interestingly, when only the BrdU$^+$ KCs were considered, neither the percentage of BrdU$^+$ cells (Figure 3J, $p = 0.319$) nor the frequency distribution of the BrdU$^+$ cells per KC varied between B-8h and B-4d (Figure S4G; Table S1, *BrdU$^+$ KCs*: $p = 0.247$). Thus, in the BrdU$^+$ KCs, neither BrdU dilution under detection levels nor the addition of new TAPs from quiescent cells occurred in the four-day chase. Hence, BrdU$^-$ KCs are not derived from loss of BrdU staining, but they actually represent newly formed KCs. To sum up, for at least 5 weeks, new neurogenic foci are continually established and undergo progressive maturation in the STR parenchyma. Since their number remains stable (Figure 3E), the genesis of new KCs must be balanced by the exhaustion of old ones. KCs are thus transient

Figure 2. Neurogenic foci exhibit heterogeneous cellular composition and are spatially independent
(A) DCX and Ki67 staining in KC subtypes.
(B) n° of cells vs. percentage of prNBs per KC; KCs associated with pmNBs are in orange.
(C) Percentage of KC types among reconstructed KC.
(D) N° of TAPs (red) and prNBs (gray) per KC type (see also Table S1).
(E) Percentage of ASCL1$^+$ cells in TAPs-only (n = 25).
(F and F′) 3D reconstruction of rostral neurogenic area and (F′) segmented KC in STR (random colors) and SVZ (blue) (see also Video S2).
(G and H) Representative G functions of STR or neurogenic area KC distribution of the specimen in (F). Gray areas: pointwise envelopes of 999 simulations of complete spatial randomness. $G_{(obs)}(r)$ (red line): experimental value; $G_{(H0,mean)}(r)$ (black dashed line): mean of the simulations. (H) Moran's I index and associated p value for each tested specimen (n = 5) and variable (n = 3) (see method details and Table S1). Data in (D) and (E) are expressed as mean ± SD. Scale: (A) 25 μm (F) 100 μm.

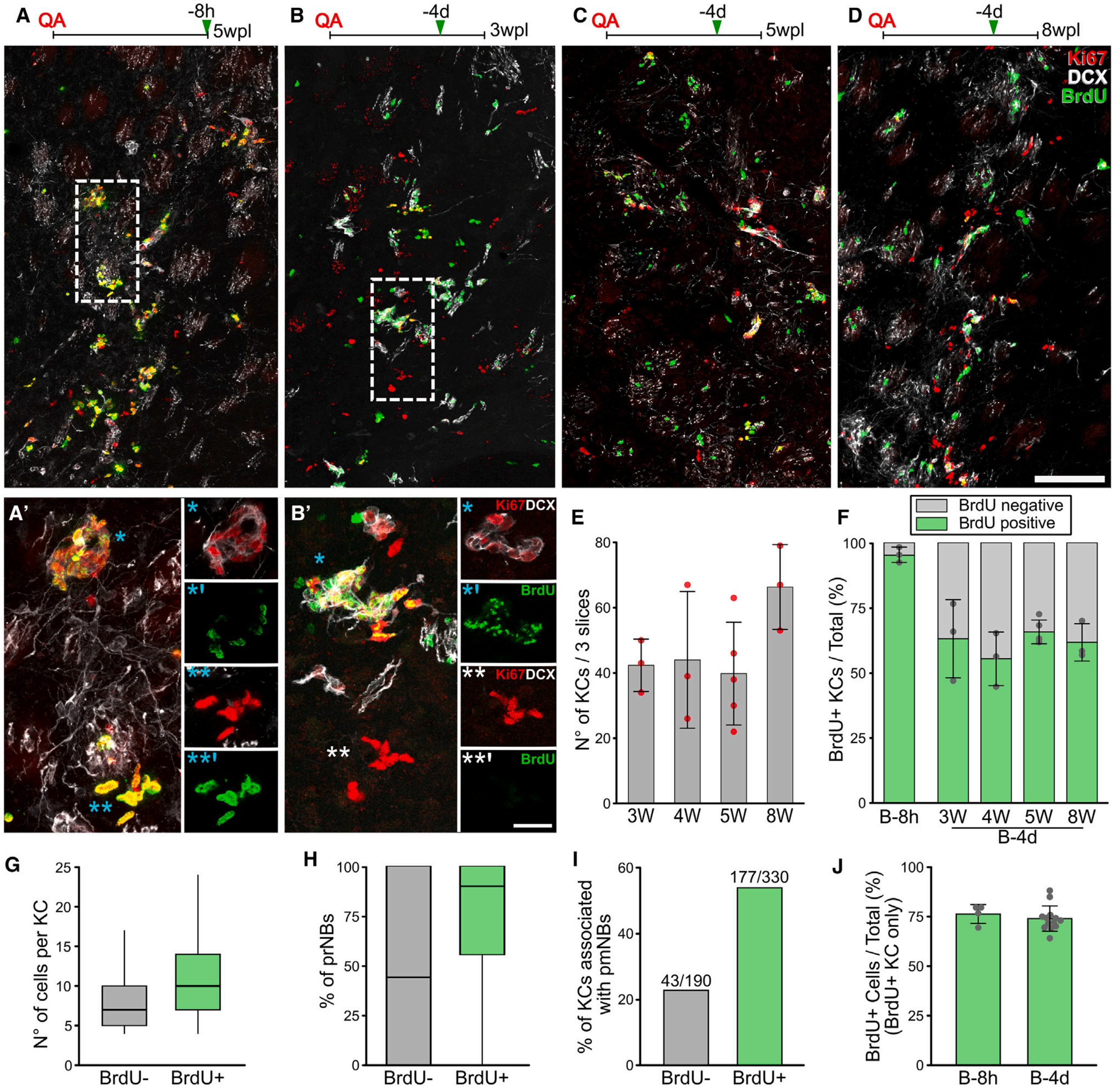

Figure 3. Neurogenic foci undergo a constant turnover

(A–D) BrdU, Ki67, and DCX labeling from B-8h (A) and B-4d specimens at 3 wpl (B), 5 wpl (C), and 8 wpl (D) in the neurogenic core.

(A′–B′) Higher magnification of (A) and (B). Maximum intensity (MAX) projections and single focal planes, respectively, in the left and right panels. * indicates more mature and ** more immature KC.

(E) n°r of KC in three non-consecutive slices.

(F) Percentage of $BrdU^+$ KC at B-8h and B-4d.

(G–I) Comparison of the n° of cells (G), percentage of prNBs (H), and the fraction associated with pmNBs (I) per KC between the $BrdU^-$ and the $BrdU^+$ KC at B-4d.

(J) Mean ± SD percentage of $BrdU^+$ cells in $BrdU^+$ KC in B-8h and B-4d specimens. Data in (E–F) and (J) are expressed as mean ± SD. Data in (G–H) are reporterd as box and whisker plots. See Table S1 for statistical analyses of (E–J). Scale: (A–D) 100 μm; (A′) and (B′) 20 μm.

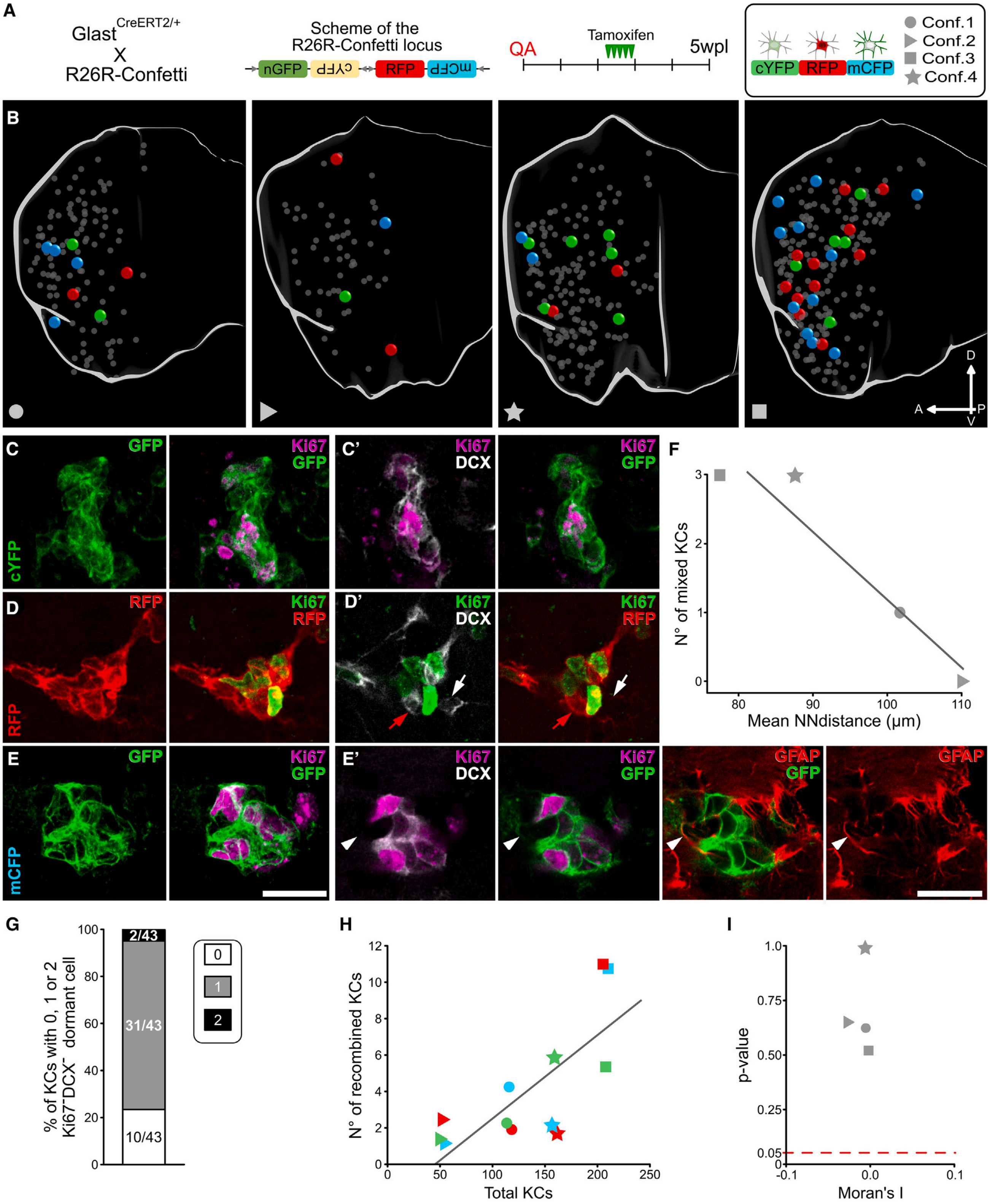

(legend on next page)

structures for which we calculated a lifetime of 10.6 ± 2.1 days and a turnover rate of 9.4%/day (Methods S1, section 5).

Together with the lack of spatial patterns in KC organization, these data suggest that in the neurogenic area KC initiation continuously occurs at random locations within a globally permissive environment.

Clonal expansion of STR ASs generates individual KCs

AS could support the establishment of these transient KCs in several ways. Each KC could arise from individual or multiple ASs. In parallel, each ASs could expand locally to generate an individual clone or produce multiple migrating progenitors that secondarily expand. To distinguish among these possibilities, we conducted a clonal analysis of STR ASs.

Glast$^{CreERT2/+}$ mice expressing the inducible form of CRE recombinase under the astrocytic promoter *Glast* (Mori et al., 2006) were crossed with *R26R*-Confetti reporter mice (Snippert et al., 2010) in which tamoxifen (TAM) administration leads to the stochastic expression of 1 out of 4 fluorescent proteins (Figures 4A and S5A). At 5 wpl, 15 days after TAM administration, 2.4% ± 0.7%, 1.2% ± 0.7%, and 1.7% ± 1.4% of the SOX9$^+$GFAP$^+$ STR ASs expressed RFP, mCFP, and cYFP, respectively (Figures S5B–S5E, see also S5I–S5J).

Whole STR 3D reconstruction of four specimens (Figure 4B) led to the identification of 537 KCs (mean ± SD = 134 ± 63), 49 of which showed reporter expression (Confetti KCs; 8.4% ± 3.3%; Figures 4C–4E′). The Confetti KC maturation profiles matched those observed at 5 wpl (Figure S5K). Among Confetti KCs, 42/49 were clones of cells expressing the same reporter (87% ± 11%; Figures 4C–4E′) and thus originated from the same AS. Non-recombined cells represented only 2% of all Confetti KC cells (25/1,048 of total counted cells) and were confined to 7 KCs. These few mixed KCs were found in the specimens with the lowest mean KCs nearest neighbor distance (Figure 4F; Table S1, $p = 0.042$), suggesting that increased density favored the rare fusion of neurogenic foci.

Interestingly, about 75% of Confetti KCs included a clonally related Ki67$^-$DCX$^-$ cell, rarely two, (Figure 4G) that in 70% of the cases we could confirm being a GFAP$^+$ AS (Figure 4E′; 10/14 tested cells). Overall, these results demonstrate that STR KCs derive almost exclusively from the clonal expansion of a single AS and suggest that it often remains associated with its progeny in a dormant state.

Further, we addressed if a single AS can give rise to multiple KCs. The number of KCs sharing the same reporter in each specimen was extremely low, ranging from 1 to 11 (mean ± SD = 4.1 ± 3.6). However, the same-color KCs were still within the expected values given the number of KCs in that specimen (Figures 4H and S5F–S5H; Table S1, $p = 0.004$). Moreover, Confetti KCs sharing the same reporter did not show any evident spatial association (Figure 4B), as confirmed statistically by Moran's I and by permutation tests (Figures 4I; Table S1). This indicates that KCs are clonally independent structures deriving from distinct ASs. The presence of a dormant AS within the neurogenic foci (Figures 4E′ and 4G) further supports this conclusion and indicates that these cells locally expand. STR neurogenic activity thus closely resembles that of canonical niches but is distributed over a much wider 3D environment, thus leading to a negligible intermixing of different AS progeny.

STR ASs activate at a constant rate

BrdU analysis did not detect any cellular turnover in neurogenic foci, strongly suggesting that AS activation initiates new KCs that subsequently autonomously expand and mature. To validate this model, we used lineage tracing by dating back the AS activation events contributing cells to 5 wpl KCs. Different cohorts of *Glast*$^{CreERT2/+}$x*R26R*-YFP animals were sacrificed at 5 wpl and received TAM 4, 7, or 14 days before sacrifice (T-4, T-7, and T-14; Figure 5A). At each time point we measured the KC labeling index (LI), that is, the fraction of KCs that received at least one YFP$^+$ cell from their AS progenitor (YFP$^+$ KCs; Figures 5B and 5C). As a reference for the maximum LI, we analyzed specimens receiving TAM before the lesion (T-bQA; Figure 5A).

Figure 4. Clonal expansion of STR ASs generates individual KC

(A) *R26R*-Confetti locus and experimental timeline. Right panel: reporter color and specimen symbols legend.

(B) Medial view of the four 3D reconstructed STR. KCs are depicted as spheres, bigger and colored for Confetti KCs, smaller and gray for the others.

(C–E) MAX projection of confetti KC: expressing cYFP, RFP, and mCFP, respectively.

(C′–E′) Single focal planes of (C), (D), and (E), respectively. Red and white arrows in (D′): color-matching and unlabeled pmNB, respectively. Arrowhead in (E′): dormant GFAP$^+$ AS.

(F) Correlation between the mean KC nearest neighbor distances and the n° of mixed KC.

(G) Percentage of Confetti KC containing 0, 1, or 2 dormant Ki67$^-$DCX$^-$ cell.

(H) Correlation between the total n° of KC and the n° of KC expressing each reporter.

(I) Moran's I index and associated *p* value calculated for each specimen (see method details and Table S1). Black lines in (F) and (H) represent linear regression models. Scale: (C–E′) 20 μm.

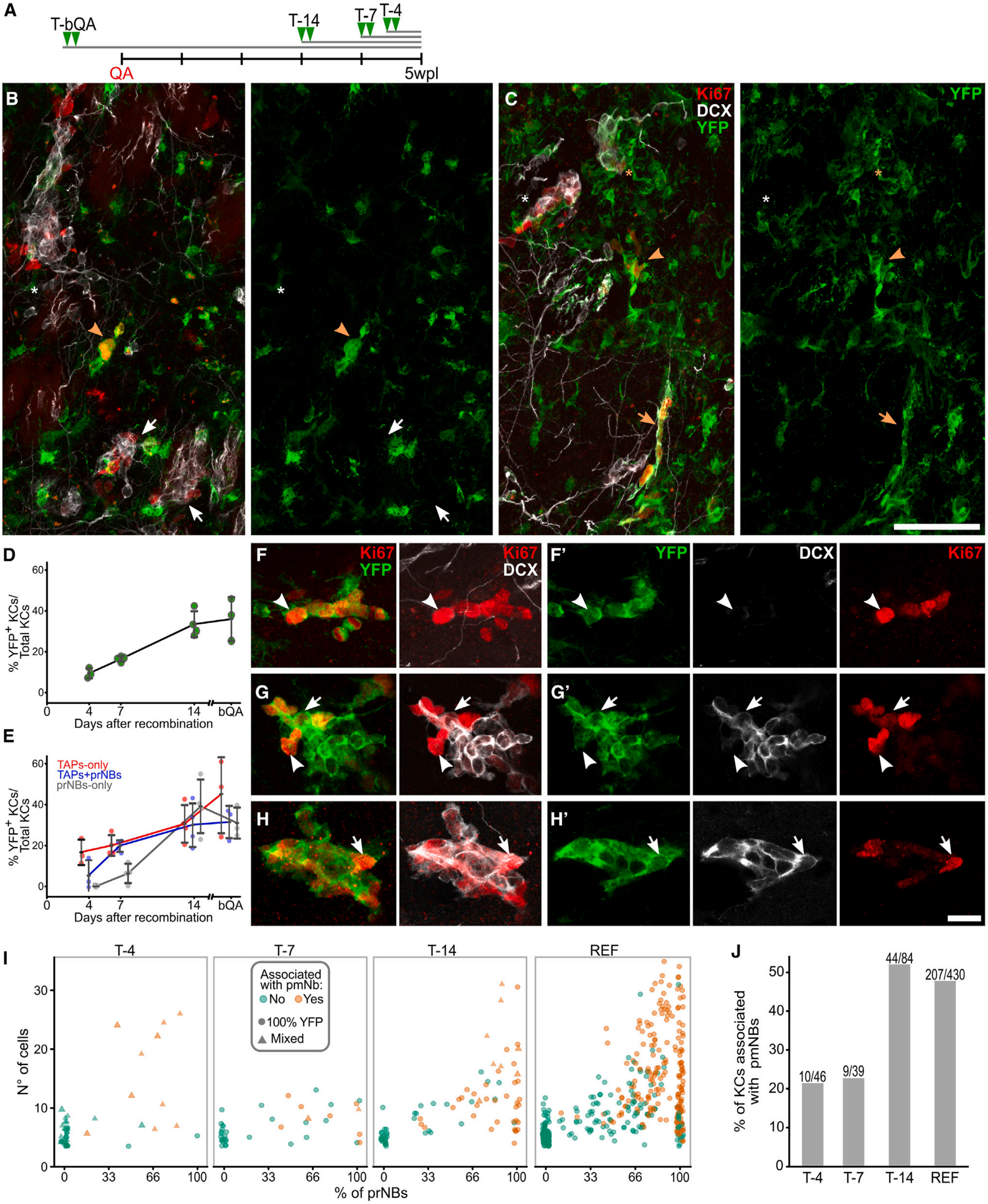

(legend on next page)

The KC LI linearly increased from 9.4% ± 2.5% in T-4 to 33.5% ± 6.3% in T-14 and remained stable in T-bQA (36.1% ± 10.9%; Figure 5D; Table S1, *p < 0.001*; *T-4* vs. *T-14: p = 0.002*; *T-14* vs. *T-bQA: p = 0.938*). This linear increase in the YFP$^+$ KC proportion means that AS activation occurs in a staggered manner at a constant rate (see mathematical analysis in the following) until it reaches a plateau: around 14 days are needed for all the 5 wpl KCs to have experienced at least one AS activation event.

Each AS activation event initiates a new KC that progressively matures

To explore if AS activation preferentially occurs in specific phases of the KC life, we evaluated the LI of the main KC subtypes across time points (Figures 5E–5H′). The LI of TAPs-only KCs was already at plateau at T-4 (Figures 5E–5F′; Table S1, *p* = 0.359), showing that TAPs-only KCs are on average within four days from an AS activation event. By contrast, TAPs+prNBs and prNBs-only KCs showed a delayed increase reaching their peaks only by T-7 and T-14, respectively (Figures 5E and 5G–5H′; Table S1, *p = 0.006, p = 0.001*). This indicates that these KC types are progressively more distant in time from the last AS activation event. In line with these observations, the number of Ki67$^+$ cells, the fraction of prNBs, and the association with pmNBs progressively increased with time (Figures 5I, S6A, S6B, and 5J; Table S1, *p < 0.001 in each case*) and became indistinguishable from the reference population by T-14 (Figures 5I, S6A, S6B, and 5J; Table S1, *T-14 vs. REF: p=0.249, p=0.549, and p=1, respectively*). Thus, STR AS activation predominantly occurs in the initial phases of KC life, giving rise to TAPs-only KCs which then gradually mature.

To further validate that AS activates only at KC initiation, we examined the prevalence of YFP labeling in YFP$^+$ KCs (Figure 6A). In contrast to T-14 and T-7, at T-4 only 40% of the YFP$^+$ KCs were 100% recombined (18/46; Figures 6A, S6A, and S6B; Table S1). These 100% YFP$^+$ KCs were almost exclusively small TAPs-only KCs (Figure S6D). Interestingly, with the exception of 5 putative fused KCs (Figures S6C–S6C′), the mixed KCs (including YFP$^+$ and YFP$^-$ cells) had only slightly more mature profiles suggesting they have a similar age as the 100% YFP KCs (Figures 6B, S6A, S6B, S6E–S6G, and S6I; Table S1). As most mixed KCs contained about 30%–60% of YFP$^+$ cells (Figure 6C), we can infer that in these KCs *Glast*$^{CreERT2/+}$-driven recombination occurred at 2- or 3-cell stage in ASs or their earliest progeny. These results confirm that AS activates virtually exclusively to initiate a new KC. As for Confetti mice, at all time points a Ki67$^-$DCX$^-$ dormant cell, in rare cases two, clustered with 75% of YFP$^+$ KCs (Figures 6D and 6E), and it turned out to be GFAP$^+$ in 26/27 tested cases (Figure 6D). Thus STR ASs rapidly return to quiescence after division and their reactivation is extremely rare, at least within KC life.

A mathematical analysis of KC initiation dynamics (Methods S1, section 1) shows that the initial slope of the increase of the KC LI over time corresponds to the KC initiation rate (Figure 5D), which also applies to the AS activation rate. Given that this slope is approximately linear, we can conclude that the AS initiation rate is approximately constant. When approaching the KC lifetime, the KC LI is expected to saturate to a plateau (Methods S1, section 1). This occurs at around 10–14 days, consistent with the previously estimated KC lifetime of 10.6 days. These results thus conclusively demonstrate that KCs undergo steady-state turnover and provide direct proof that ASs initiate new KCs that progressively mature before undergoing exhaustion.

ASs activate in neurogenic foci-free areas

The aforementioned data predict that ASs activate in KC-free areas. To directly verify this possibility, we analyzed the organization and distribution of proliferating ASs in the neurogenic area at 5 wpl (Figures 6F–6I, S6I, and S2G–S2G′). We distinguished between cells expressing SOX9 at similar or slightly lower levels as the non-proliferating ASs (SOX9^{High}) from those showing substantially lower levels (SOX9^{Low}; Figures 6F–6I) that may represent TAPs (Cheng et al., 2009). Most Ki67$^+$SOX9$^+$ cells were in groups smaller than four cells with a strong prevalence for pairs (Figures 6G and 6J). The fraction of SOX9^{High} cells among Ki67$^+$SOX9$^+$ cells progressively decreased from 78% in individual cells to 62% in pairs, 44% in trios, and 7% in KCs (Figures 6F–6I). This decrease was paralleled by an increase

Figure 5. Constant AS activation generates TAPs-only KCs that autonomously mature overtime

(A) Experimental timeline.

(B and C) YFP$^+$ and YFP$^-$ KC in T-4 (B) and T-14 (C). Arrowheads: TAPs-only; asterisks: TAPs+prNBs; arrows: prNBs-only. White and orange: YFP$^-$ and YFP$^+$ KC, respectively.

(D) LI by TAM administration of all KC or (E) divided by KC type.

(F–H) MAX projections of completely YFP$^+$ TAPs-only (F), TAPs+prNBs (G), and prNBs-only (H) KC.

(F′–H′) Single focal planes of (F–H), respectively. Arrowheads: TAPs; arrows: prNBs.

(I) Percentage of prNBs vs. n° of cells per KC at different time points (see also Figures S6A and S6B). KCs associated with pmNBs are in orange. REF as in Figure 2B. Circles: fully recombined KC; triangles: mixed YFP$^+$ and YFP$^-$ KC.

(J) Percentage of KC associated with pmNBs at different time points and in the REF (see also Table S1). Data in (D) and (E) are shown as mean ± SD at each time point (see also Table S1). Scale: (B and C) 50 μm; (F–H′) 15 μm.

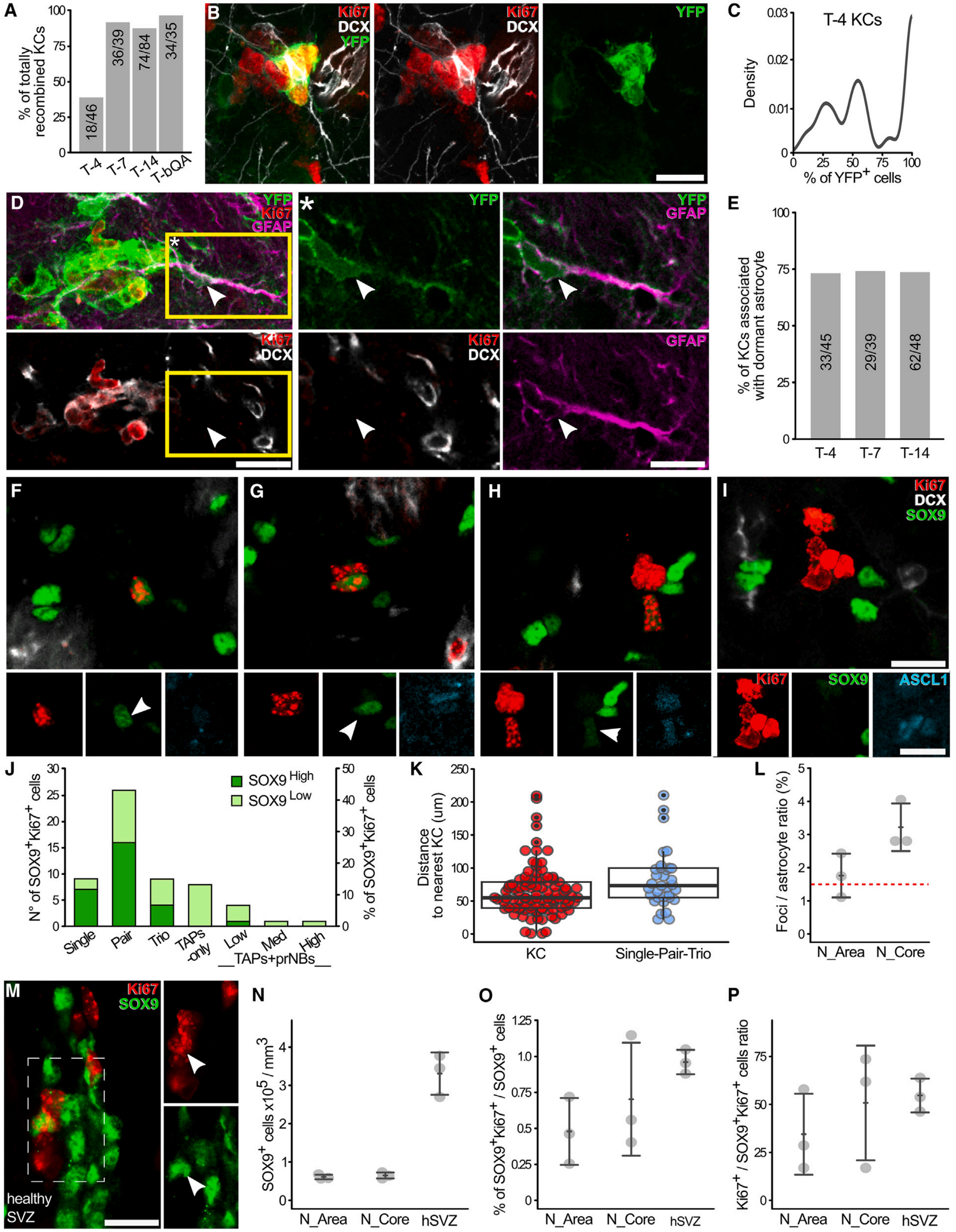

(legend on next page)

in ASCL1 expression (Figure S6H; Table S1, $p < 0.001$ in both cases) confirming that early neurogenic foci cells lose their astrocytic identity before reaching the KC stage.

The groups of less than four Ki67+ cells containing activated ASs never contacted pmNBs, and their nearest KC was on average farther away than the mean nearest neighbor distance between KCs (Figures 6K and S6I; Table S1, $p = 0.013$). These results show that STR ASs preferentially activate in areas devoid of pre-existing KCs and far from previous activation events.

STR and SVZ ASs activate at similar rates

Neurogenic foci represent activation events that occurred over the last 10 days. To evaluate STR AS activation rate, we thus evaluated the KC/AS ratio in the neurogenic area or its core, respectively, comprising 95% or 25% of the KCs. The AS density did not differ between these areas, and, although it tended to be higher than in the rest of the STR (Figure S6I), it was still about 5 times lower than in the SVZ of healthy mice (hSVZ; Figures 6M and 6N; Table S1, *N_Area vs. hSVZ:* $p = 0.0001$, *N_Area vs. hSVZ:* $p = 0.0001$). The KC/AS ratio ranges from 1% to 4%, corresponding on average to 0.18% activations per day in the neurogenic area, a value that is strikingly similar to that calculated for SVZ ASs (0.15% activations per day; Figure 6L; Calzolari et al., 2015). In support of this observation, the fraction of proliferating ASs (Ki67+SOX9+/SOX9+ cells) as well as the ratio between these cells and clustered Ki67+ cells in SVZ and STR were also similar (Figures 6O and 6P; Table S1, $p = 0.168$, $p = 0.52$). STR ASs thus share with SVZ ASs not only the activity pattern but also the activation rate, implying a comparable prevalence of NSC potential.

Reactivity of neurogenic ASs

Neurogenic activation and reactivity are independent AS states that can co-occur, particularly after acute lesions inducing AS proliferation (Sirko et al., 2013). To unveil the overall relationships between these states after QA, we analyzed the AS reactive response. At 1 wpl, STR ASs were all strongly reactive: they were hypertrophic, and virtually all upregulated GFAP, NESTIN, and C3 (Figure S7A–S7K; Escartin et al., 2021; O'Shea et al., 2024). Inside the lesion they further strongly proliferated at this stage (Figures S7A–S7C; Table S1). Interestingly, at 1 wpl, C3 was transiently upregulated also in virtually all SVZ ASs, indicating that it is compatible with the neurogenic state (data not shown). At 5 wpl, while generic features of AS reactivity such as GFAP expression were still present, NESTIN and to a lesser extent C3 expression was decreased and mostly confined to the lesion and bordering intact tissue (Figures S7F–S7K; Table S1). In this area, both proliferating SOX9+ and *Glast*-CreERT2::YFP+ quiescent SOX9+ ASs associated with YFP+ KCs were similarly heterogeneous for the expression of C3 as their SOX9+ neighbors (Figures S7I–S7O). Thus, as in other models of acute lesions (Escartin et al., 2021; O'Shea et al., 2024), QA causes an early strong proliferative AS reactivity that subsequently matures in border ASs showing a reduced but persistent reactive state. Neurogenically active ASs are part of the overall AS reactivity, but interestingly their activation probability does not perfectly coincide with the early AS proliferation gradient being more equally distributed around the lesion border (Figure S7D). Activation of the neurogenic program is thus likely compatible with multiple reactive AS states, at least after QA.

Cell fate choice timings in the turnover of neurogenic foci

As ASs divide mainly once per KC, TAPs and prNBs account for most of the NB production. The autonomous growth and steady-state turnover of STR neurogenic clones allowed us to exploit our rich collection of reconstructed KCs

Figure 6. ASs activate in KC-free areas and shortly come back to quiescence
(A) Percentage of fully YFP+ over total YFP+ KC.
(B) Immature mixed KC at T-4.
(C) Frequency distribution of the fraction of YFP+ cells per KC at T-4.
(D) YFP+ KC associated to a dormant GFAP+ AS. (*) single focal plane of the yellow box in (D).
(E) Percentage of KC associated with at least a YFP+ dormant cell over time.
(F–I) Single (F), pair (G), trio (H), or clustered (I) Ki67+ cells in which at least 1 proliferating cell is SOX9+. The lower panels show SOX9 and ASCL1 expression in Ki67+ cells. Arrowheads: SOX9^{High} cells; arrows SOX9^{Low} cells.
(J) n°(left) and percentage (right) of Ki67+SOX9^{High} and SOX9^{Low} cells in Ki67+ groups.
(K) Comparison of the distance to the nearest KC between KC (red) and single/pair/trio (gray).
(L) Foci/AS ratio in the neurogenic area (N_Area) and in its core (N_Core). Red dashed line: AS activation rate in the SVZ (Calzolari et al., 2015).
(M) A SOX9^{High}Ki67+ cell (arrowhead) in the SVZ of a healthy mouse. Left panel: MAX projection. Right panel: single focal plane of the dashed rectangle.
(N–P) Comparison of the AS density (N), the percentage of proliferating ASs (O), and the ratio between TAPs/prNBs and proliferating ASs, among STR neurogenic areas and healthy SVZ. Data in (K) are reported as box and whisker plot. Data in (L) and (N–P) are expressed as mean ± SD. Scale: (B), (D), (F–I), and (M) 15 μm.

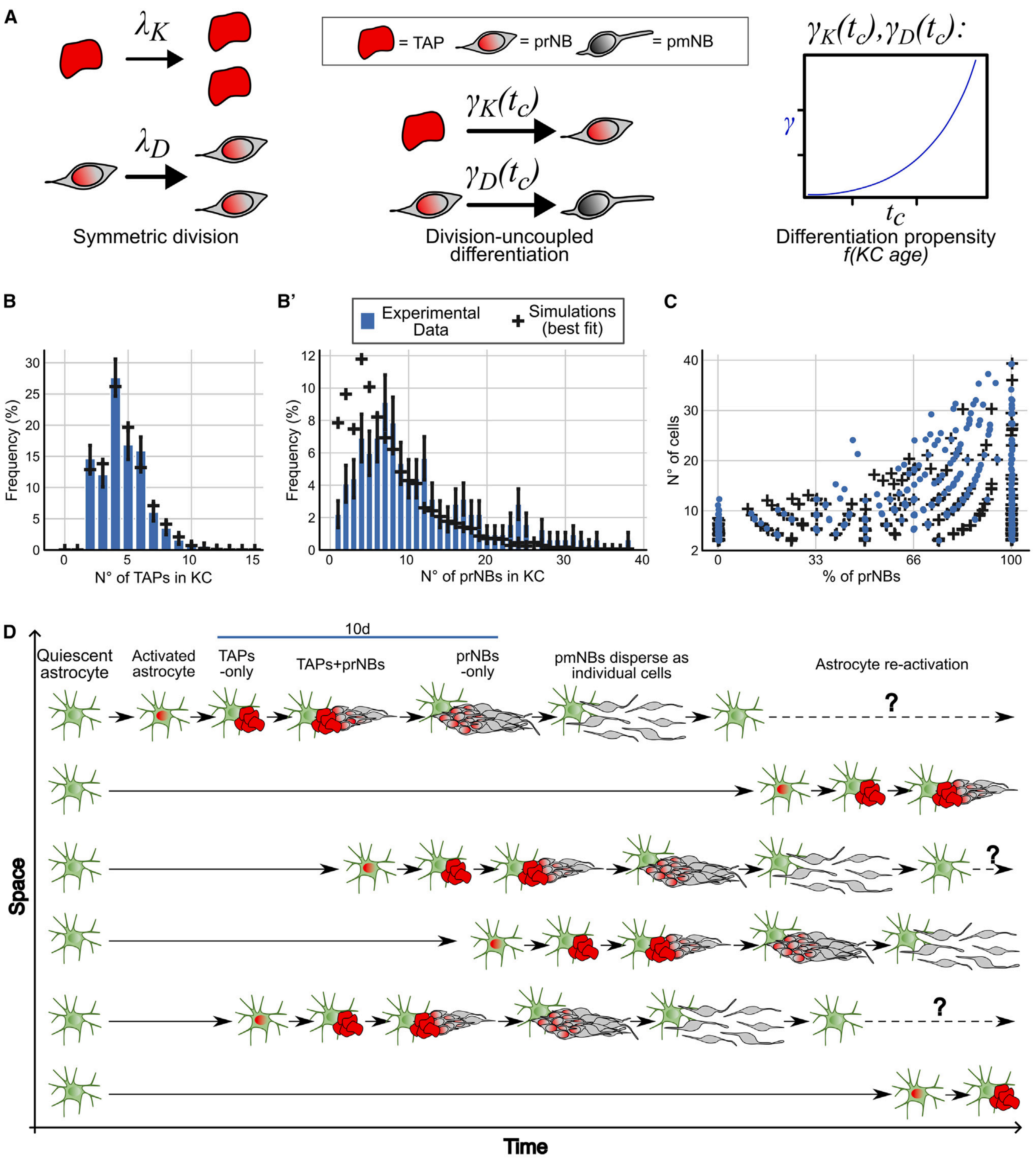

Figure 7. Mathematical modeling of cell fate choices in KC

(A) Schematics of the best fitting model features. TAPs and prNBs mostly undergo symmetric duplicating division with λ_K and λ_D rates, respectively. The differentiation propensities of TAPs and prNBs (γ_K and γ_D, respectively) change as a power law function over the KC age.

(legend continued on next page)

(Figure 2) to model TAPs and prNBs expansion and fate choices. Intermediate progenitors are usually thought to expand stereotypically; however, their behavior is still poorly characterized (Götz, 2018). We implemented a stochastic mathematical model (Haccou et al., 2007; Methods S1) to discern (1) how the differentiation propensity (TAP → prNB; prNB → pmNB transitions) varies over time and whether it depends on KC ages and (2) whether the differentiation of TAPs and prNBs is coupled to cell division via asymmetric and symmetric divisions, or whether it can occur independently of cell divisions.

Models of the distribution of TAPs per KC showed an excellent fit by allowing cell fate choices to be stochastic, yet only if the differentiation propensity increases in an accelerated way over time (Figures 7A and 7B; Methods S1; section 4.1.3). The best fit is achieved if we assume that differentiation can occur independently of cell division although we cannot strictly exclude the option that differentiation is coupled to symmetric cell division (Methods S1, sections 4.1.2 and 4.1.3).

As for TAPs, only if the prNB differentiation propensity increases over time in an accelerated way, do we obtain a reasonable model fit (Figures 7A and 7B′; Methods S1; section 4.2). However, for prNBs, we can exclude that differentiation is coupled to cell division; thus, the exit of the cell cycle occurs independently of the previous division. We note that some deviations between data and model predictions remain even for the best fit, for very small and very large cell numbers, respectively, over- and underrepresented in the model (Figure 7B′). This is likely due to the dispersion of the last prNBs within clustered NB at KC end (very small) and KC fusions (very large).

Neurogenic foci cells thus show features of both stochastic and deterministic fate choices that ultimately result in their invariant exit from the cell cycle but with consistent variability. Hence, these data fully validate the presented model of neurogenic foci initiation, maturation, and dynamic turnover (Figures 7C and 7D) and mechanistically confirm the intermediate progenitor nature of the KC cells.

DISCUSSION

Here, we demonstrated that the spatiotemporal pattern of STR AS neurogenic activity closely resembles that of classic adult vertebrate brain niches. However, these patterns emerged within the 3D lattice of parenchymal ASs, distinguishing them from conventional NSC monolayers. STR AS activation events were constant in time, at a rate similar to SVZ ASs, and random in space resulting in a steady state of continuous and widespread neurogenesis. The mouse STR can thus be considered as a dormant neurogenic niche hosting a widespread population of qNSCs that can be focally activated.

NSC activation dynamics in the STR neurogenic niche

The local expansion and minimum overlap of AS proliferating progeny allowed us to use neurogenic foci as a proxy for the location of ASs activation events over the last ~10 days. By integrating two complementary dynamic approaches, we unveiled that ASs neurogenically activate at a constant rate. In parallel, the neurogenic foci spatial independence revealed that activation events are randomly distributed in space and time. These events preferentially occur in new locations, far from pre-existing foci, overall demonstrating the widespread presence of neurogenically competent ASs within the STR. A similar random pattern of NSC activations inhibited by previous events was described in zebrafish (Dray et al., 2021b). In that case, inhibition was proposed to be mediated by NBs, which inhibit NSC activation also in mammalian niches through γ-aminobutyric acid (GABA) release or cell-cell contacts (Liu et al., 2005; Rolando et al., 2012). The fact that proliferating STR ASs rarely contact NBs supports the hypothesis that similar inhibitory mechanisms might be at play in the STR. Following activation, in both SVZ and SGZ, NSCs undergo consuming neurogenic divisions with high probability (Calzolari et al., 2015; Obernier et al., 2018; Pilz et al., 2018), while asymmetric self-renewing divisions characterize neurogenesis during aging (Bast et al., 2018; Harris et al., 2021). This latter mode of division is preferred also by STR ASs, ensuring the maintenance of the population. Of note, a recent study suggests that in the SVZ NSCs divide always symmetrically and daughter cells locally compete for the occupancy of "a restricted niche" (Basak et al., 2018). According to this model, the lower AS density in the parenchyma may increase the chance of at least one cell reoccupying the niche. The division of TAPs and NBs was regulated by stochastic processes although an accelerating differentiation propensity introduces a deterministic factor constraining their expansion. Similar stochastic behavior of TAPs has been described in the SGZ by *in vivo* imaging (Götz, 2018; Pilz et al., 2018).

(B and B′) Best model fitting of TAPs (B) and prNBs (B′) frequency distributions in KC. Bars are measured frequencies, and black crosses represent simulation output, obtained from the best fitting model (see also Methods S1).
(C) Scatterplot showing the n° of cells vs. percentage of prNBs per KC. Similar to Figure 2B, with the addition of simulated KC obtained from the best fitting model (data not used for fitting the model).
(D) Scheme of the spatiotemporal dynamics of STR AS activation and lineage progression.

Neurogenic permissiveness of the STR parenchyma

Our results demonstrated that the STR acts as a neurogenic niche by constitutively hosting a widespread population of dormant NSCs. Notably, all major known NSC quiescence stimulating pathways such as NOTCH (Imayoshi et al., 2010), BMP (Lim et al., 2000), β1 INTEGRIN (Porcheri et al., 2014), S1P (Codega et al., 2014), or GABA (Liu et al., 2005; Song et al., 2012) are active in parenchymal AS (Lim et al., 2000; Magnusson et al., 2020; Nagai et al., 2019; Robel et al., 2009; Singh et al., 2022). Unlike canonical niches, in the parenchyma, inhibition of these pathways alone mostly fails to induce neurogenic activation. Nonetheless, at least for NOTCH inhibition, single-cell RNA-seq indicates induction of a primed NSC state (Magnusson et al., 2020; Zamboni et al., 2020). Acute lesions inducing ASs to proliferate and upregulate immature markers such as NESTIN can also reduce NSC quiescence in both canonical niches (Llorens-Bobadilla et al., 2015), and parenchyma (Sirko et al., 2023; Zamboni et al., 2020). Only in the striatum after QA, stroke, and to a lesser extent NOTCH inhibition (Magnusson et al., 2014; Nato et al., 2015) however, these state transitions progress to neurogenic activation *in vivo*, suggesting that additional signals are needed to regulate this switch. After QA, neurogenic ASs were likely part of the early reactivity response and still show some reactive features during the neurogenic phase, such as GFAP, NESTIN, and some C3 expression. Interestingly, however, both lesioned and intact sides of the lesion border, originally associated with strong or no AS proliferation, can host neurogenic activation later on. Activation of the neurogenic program is also temporally dissociated from this early proliferative response as it starts during the third wpl, which may be consistent with the tissue remodeling phase (Burda and Sofroniew, 2014). This suggests that neurogenic activation is compatible with multiple AS reactive states and could be regulated by specific factors. Moreover, although neurogenic potential was present throughout the STR, it was more likely to activate in the medial functional domain. Interestingly, this domain showed a higher frequency of neurogenic foci also in normal rabbits (Luzzati et al., 2006), under progressive degeneration (Luzzati et al., 2011a), stroke, or NOTCH inhibition (Magnusson et al., 2014). Activatory stimuli diffusing from the SVZ have been proposed to cause this spatial bias (Magnusson et al., 2020); however, at least after QA the pattern of activation events was not shaped as an SVZ centered gradient but peaked more laterally, at the lesion border. This suggests the involvement of local factors preferentially associated with the overlap between the medial striatum and the mature lesion border. Whether the medial striatum has a higher density of dormant NSCs or is subjected to stronger stimulating factors remains to be determined. The existence of such factors is supported by the high variability in neurogenic foci number in both pathological (this work) and physiological (Luzzati et al., 2006) conditions. An intriguing possibility is that activation factors may be related to neuronal afferents, which have been shown to modulate neurogenic activation in neurogenic niches (Káradóttir and Kuo, 2018) and represent the main structural difference between the striatal domains.

How widespread is the neurogenic potential among ASs?

NOTCH pathway blockage causes widespread NSC priming in cortical and STR ASs (Magnusson et al., 2020; Zamboni et al., 2020), leading the authors to speculate that all AS possess a neurogenic potential. Here, we demonstrate that naive STR ASs do have such widespread potential. Establishing whether all STR ASs harbor NSC potential is challenging, a question that is unresolved even for the canonical NSC (Dray et al., 2021a; Obernier et al., 2018). The close similarity in both the pattern and rate of neurogenic activation indicates that these two populations have comparable prevalence of NSCs. However, as in both cases the reactivation rate is unknown, the exact AS fraction that is recruited to neurogenesis cannot be established. In STR, ASs do not reactivate at least over the KC lifetime, but their quiescence likely lasts longer because (1) clusters of pmNBs remain for some time after proliferative pool exhaustion (data not shown) and (2) AS activate far from pre-existing KC. Moreover, the local activation density may be much higher than the average, as suggested by cases of KC fusion. Thus, the maximum density of recruited ASs, a proxy of the neurogenic potential per unit area, is likely much higher than our measure over 10 days (up to 1/25 ASs), potentially including all ASs.

In non-mammalian vertebrates, radial glia persists into adulthood where they act both as NSC and AS-like support cells (Jurisch-Yaksi et al., 2020). In mammals, it was thought that these two functions were fulfilled by distinct cell types and that AS differentiation implies a permanent loss of NSC potential. However, our results definitely demonstrate that this potential can be maintained in parenchymal ASs. Whether all AS subtypes/states have the same probability to activate an NSC potential, both between and within regions, remains to be established. NSCs are a heterogeneous population of progenitors committed to generate specific neuron types and, at least in adults, are regulated by distinct conditions and neuronal circuits (Chaker et al., 2023; Káradóttir and Kuo, 2018). Within the mammalian parenchyma, ASs, while specializing in local circuit-specific control, may have also acquired a circuit-specific regulation of their neurogenic capacity.

We already showed activation of a dormant niche in the ventral STR around weaning in guinea pigs, but, similarly

to the dorsal SVZ, this niche was confined to the narrow pallial-subpallial-boundary (Luzzati et al., 2014). Our new study now extends the complexity of adult NSC spatial heterogeneity to the 3D lattice of parenchymal ASs. This radically challenges a traditional view of neurogenic niches as unique domains for stem cell maintenance acting as barriers to parenchymal differentiation signals. The brain may rather have a widespread quiescent neurogenic potential organized as a mosaic of progenitor domains regulated by specific activation signals. In canonical niches, these signals are constitutively active, while in other regions they may be triggered only in specific conditions. The size, distribution, regulation, and cell fate potential of these progenitor domains await further analysis, thereby paving the way for a deeper understanding of the full adult brain neurogenic potential.

Limitation of the study

We could not precisely measure how many ASs harbor an NSC potential, yet distinguishing between reversible state transitions of multipotent cells and epigenetically constrained subpopulations is a major unsolved issue in AS and NSC biology. Our data provided the first indication for the dynamic regulation of a functionally defined AS reactive state, specifically the neurogenically active state, and suggest that it acts as an independent module. However, more detailed analyses of the spatiotemporal dynamics of other functionally defined AS states/modules will be required to understand their neurogenic potential and more in general to define the limits and regulation of AS plasticity.

EXPERIMENTAL PROCEDURES

Experimental model and subject details

Animal procedures

The experimental plan was designed according to the guidelines of the European Communities Council (2010/63/EU) and the Italian Law for Care and Use of Experimental Animals (DL26/2014). It was also approved by the Italian Ministry of Health (authorization 327/2020-PR) and the Bioethical Committee of the University of Turin. The study was conducted according to the Animal Research: Reporting of In Vivo Experiments guidelines.

Mouse lines

Experiments were performed on 8- to12-weeks-old animals of the following mouse lines: C57BL/6J mice (Harlan Laboratories; n = 18 males), *Glast*$^{\mathrm{CreERT2}}$ (Mori et al., 2006), *R26R*-YFP (Srinivas et al., 2001), and *R26R*-Confetti (Snippert et al., 2010).

Method details

Stereotaxic injections

Mice were anesthetized with 0.3 mL/kg ketamine (Ketavet, Gellini) and 0.2 mL/kg xylazine (Rompun, Bayer), positioned in a stereotaxic apparatus (Stoelting) and injected with a pneumatic pressure injection apparatus (Picospritzer II, General Valve Corporation). Injection coordinates are as follows: QA (1 μL; diluted to 120 mM in 0.1 M PB), +0.1 mm AP, −2.1 mm ML, and −2.6 mm DV.

BrdU pulse labeling

C57BL/6J lesioned mice received two intraperitoneal injections of BrdU (Merck; 50 mg/kg in 0.1 M Tris pH 7.4) 6 h apart and were sacrificed either 2 h (B-8h) or 4 days (B-4d) after the last injection. Animals of the B-8h group were sacrificed at 5 wpl while B-4d at 3, 4, 5, or 8 wpl.

TAM administration

TAM (Merck) was dissolved in corn oil (Merck) and administered by oral gavage at a dose of 2.5 mg per administration with a 24-h interval. *Glast*$^{\mathrm{CreERT2/+}}$x*R26R*-YFP mice received 2 TAM administrations, while *Glast*$^{\mathrm{CreERT2/+}}$x*R26R*-Confetti mice received 5 administrations.

Histology and immunofluorescence staining

Coronal sections of 50 μm were obtained and immunostained as described previously (Nato et al., 2015, see also supplemental information). Images were acquired on a Leica SP5 confocal microscope (Leica Microsystems) equipped with 40× and 63× objectives (HCX PL APO lambda blue: 40×, NA 1.25; 63×, NA 1.4). Voxel size is 0.76 × 0.76 × 2.5 μm, for whole striatal 3D reconstruction, 0.38 × 0.38 × 1.5 μm for AS and KC analyses, and 0.24 × 0.24 × 1 μm for GFAP staining.

3D reconstructions

3D reconstructions were performed by modifying a previous method (Luzzati et al., 2011b). For detailed protocol, see supplemental information.

Imaris visualization

Videos S1 and S2 and Figures 1B–1B‴ and 2F–2F′ were prepared in Imaris by importing the Ki67 and DCX channels of aligned #N1 and #G14.3 specimens in Imaris (v9.7.2). After adjusting brightness and contrast, the SVZ was manually drawn in order to exclude the fluorescence in that area. KCs in Videos S1 and S2 and Figures 1B′–1B‴′ and 2F′ were segmented semi-automatically based on fluorescence intensity, object size, and striatal location.

Data visualization

See supplemental information.

Statistical analysis

Quantifications

See supplemental information.

Spatial statistics (point pattern analysis)

To define KC distribution, we used the G(r) function. To analyze the 3D distribution of KC features, we used the Moran's I index of global spatial autocorrelation (see supplemental information).

RESOURCE AVAILABILITY

Lead contact

Further information and requests for resources and reagents should be directed to the corresponding author, Federico Luzzati (federico.luzzati@unito.it).

Materials availability

This study did not generate any unique reagents.

Data and code availability

Primary data and information to re-analyze them are available from the lead contact upon request. The collection of scripts originally created to perform the 3D reconstructions is available at the following link: https://github.com/bunbunet/FogliNato2024_3Drec.

ACKNOWLEDGMENTS

We thank Sara Trova and Alessia Caramello for preliminary work; Valentina Cerrato, Stefano Zucca, and Ilaria Bertocchi for critically reviewing the manuscript; and reviewer 2 for urging the analysis of AS reactivity. This research was supported by: Fondazione Cecilia Gilardi, Fondazione Umberto Veronesi, funding from PNRR MUR – M4C2 – Investimento 1.4 to P.P., the European Union's Horizon 2020 research and innovation programme under grant agreement no. 874758 to A.B., funds of the University of Turin and Compagnia di San Paolo (S1618 grant) to A.B. and F.L., MIUR project "Dipartimenti di Eccellenza 2018–2022 and 2023–2027 to Dept. of Neuroscience "Rita Levi Montalcini.", and UKRI Medical Research Council grant (MR/R026610/1) to P.G.

AUTHOR CONTRIBUTIONS

Conceptualization, M.F., G.N., and F.L.; software – 3D reconstructions, F.L.; investigation, M.F. and G.N.; formal analysis, M.F., G.N., and F.L.; spatial analyses, M.F.; mathematical modeling, P.G.; formal analyses – BrdU time course, J.P.; AS proliferation time course, M.R.; 3D reconstruction assistance, G.V.; writing – original draft, M.F., G.N., P.G., and F.L.; writing – review and editing, M.F., G.N., P.G., P.P., A.B., and F.L.; visualization, M.F., G.N., and F.L.; supervision, P.P., A.B., and F.L.; funding acquisition, P.P., A.B., and F.L.

DECLARATION OF INTERESTS

The authors declare no competing interests.

SUPPLEMENTAL INFORMATION

Supplemental information can be found online at https://doi.org/10.1016/j.stemcr.2024.08.006.

Received: March 23, 2024
Revised: August 20, 2024
Accepted: August 21, 2024
Published: September 19, 2024

REFERENCES

Altshuler, A., Wickström, S.A., and Shalom-Feuerstein, R. (2023). Spotlighting adult stem cells: advances, pitfalls, and challenges. Trends Cell Biol. *33*, 477–494.

Basak, O., Krieger, T.G., Muraro, M.J., Wiebrands, K., Stange, D.E., Frias-Aldeguer, J., Rivron, N.C., van de Wetering, M., van Es, J.H., van Oudenaarden, A., et al. (2018). Troy+ brain stem cells cycle through quiescence and regulate their number by sensing niche occupancy. Proc. Natl. Acad. Sci. USA *115*, E610–E619.

Bast, L., Calzolari, F., Strasser, M.K., Hasenauer, J., Theis, F.J., Ninkovic, J., and Marr, C. (2018). Increasing Neural Stem Cell Division Asymmetry and Quiescence Are Predicted to Contribute to the Age-Related Decline in Neurogenesis. Cell Rep. *25*, 3231–3240.e8.

Boda, E., Nato, G., and Buffo, A. (2017). Emerging pharmacological approaches to promote neurogenesis from endogenous glial cells. Biochem. Pharmacol. *141*, 23–41.

Bond, A.M., Ming, G.-L., and Song, H. (2015). Adult Mammalian Neural Stem Cells and Neurogenesis: Five Decades Later. Cell Stem Cell *17*, 385–395.

Buffo, A., Rite, I., Tripathi, P., Lepier, A., Colak, D., Horn, A.P., Mori, T., and Götz, M. (2008). Origin and progeny of reactive gliosis: A source of multipotent cells in the injured brain. Proc. Natl. Acad. Sci. USA *105*, 3581–3586.

Burda, J.E., and Sofroniew, M.V. (2014). Reactive gliosis and the multicellular response to CNS damage and disease. Neuron *81*, 229–248.

Calzolari, F., Michel, J., Baumgart, E.V., Theis, F., Götz, M., and Ninkovic, J. (2015). Fast clonal expansion and limited neural stem cell self-renewal in the adult subependymal zone. Nat. Neurosci. *18*, 490–492.

Chaker, Z., Segalada, C., Kretz, J.A., Acar, I.E., Delgado, A.C., Crotet, V., Moor, A.E., and Doetsch, F. (2023). Pregnancy-responsive pools of adult neural stem cells for transient neurogenesis in mothers. Science *382*, 958–963.

Cheng, L.-C., Pastrana, E., Tavazoie, M., and Doetsch, F. (2009). miR-124 regulates adult neurogenesis in the subventricular zone stem cell niche. Nat. Neurosci. *12*, 399–408.

Codega, P., Silva-Vargas, V., Paul, A., Maldonado-Soto, A.R., Deleo, A.M., Pastrana, E., and Doetsch, F. (2014). Prospective identification and purification of quiescent adult neural stem cells from their in vivo niche. Neuron *82*, 545–559.

Dray, N., Than-Trong, E., and Bally-Cuif, L. (2021a). Neural stem cell pools in the vertebrate adult brain: Homeostasis from cell-autonomous decisions or community rules? Bioessays *43*, 2000228.

Dray, N., Mancini, L., Binshtok, U., Cheysson, F., Supatto, W., Mahou, P., Bedu, S., Ortica, S., Than-Trong, E., Krecsmarik, M., et al. (2021b). Dynamic spatiotemporal coordination of neural stem cell fate decisions occurs through local feedback in the adult vertebrate brain. Cell Stem Cell *28*, 1457–1472.e12.

Escartin, C., Galea, E., Lakatos, A., O'Callaghan, J.P., Petzold, G.C., Serrano-Pozo, A., Steinhäuser, C., Volterra, A., Carmignoto, G., Agarwal, A., et al. (2021). Reactive astrocyte nomenclature, definitions, and future directions. Nat. Neurosci. *24*, 312–325.

Götz, M. (2018). Revising concepts about adult stem cells. Science *359*, 639–640.

Götz, M., Sirko, S., Beckers, J., and Irmler, M. (2015). Reactive astrocytes as neural stem or progenitor cells: In vivo lineage, In vitro potential, and Genome-wide expression analysis. Glia *63*, 1452–1468.

Haccou, P., Jagers, P., and Vatutin, V.A. (2007). Branching Processes: Variation, Growth, and Extinction of Populations (Cambridge University Press).

Harris, L., Rigo, P., Stiehl, T., Gaber, Z.B., Austin, S.H.L., Masdeu, M.D.M., Edwards, A., Urbán, N., Marciniak-Czochra, A., and Guillemot, F. (2021). Coordinated changes in cellular behavior ensure

the lifelong maintenance of the hippocampal stem cell population. Cell Stem Cell *28*, 863–876.e6.

Hintiryan, H., Foster, N.N., Bowman, I., Bay, M., Song, M.Y., Gou, L., Yamashita, S., Bienkowski, M.S., Zingg, B., Zhu, M., et al. (2016). The mouse cortico-striatal projectome. Nat. Neurosci. *19*, 1100–1114.

Imayoshi, I., Sakamoto, M., Yamaguchi, M., Mori, K., and Kageyama, R. (2010). Essential roles of Notch signaling in maintenance of neural stem cells in developing and adult brains. J. Neurosci. *30*, 3489–3498.

Jurisch-Yaksi, N., Yaksi, E., and Kizil, C. (2020). Radial glia in the zebrafish brain: Functional, structural, and physiological comparison with the mammalian glia. Glia *68*, 2451–2470.

Káradóttir, R.T., and Kuo, C.T. (2018). Neuronal Activity-Dependent Control of Postnatal Neurogenesis and Gliogenesis. Annu. Rev. Neurosci. *41*, 139–161.

Laywell, E.D., Rakic, P., Kukekov, V.G., Holland, E.C., and Steindler, D.a. (2000). Identification of a multipotent astrocytic stem cell in the immature and adult mouse brain. Proc. Natl. Acad. Sci. USA *97*, 13883–13888.

Lim, D.A., Tramontin, A.D., Trevejo, J.M., Herrera, D.G., García-Verdugo, J.M., and Alvarez-Buylla, A. (2000). Noggin antagonizes BMP signaling to create a niche for adult neurogenesis. Neuron *28*, 713–726.

Liu, X., Wang, Q., Haydar, T.F., and Bordey, A. (2005). Nonsynaptic GABA signaling in postnatal subventricular zone controls proliferation of GFAP-expressing progenitors. Nat. Neurosci. *8*, 1179–1187.

Llorens-Bobadilla, E., Zhao, S., Baser, A., Saiz-Castro, G., Zwadlo, K., and Martin-Villalba, A. (2015). Single-Cell Transcriptomics Reveals a Population of Dormant Neural Stem Cells that Become Activated upon Brain Injury. Cell Stem Cell *17*, 329–340. https://doi.org/10.1016/j.stem.2015.07.002.

Luzzati, F., De Marchis, S., Fasolo, A., and Peretto, P. (2006). Neurogenesis in the caudate nucleus of the adult rabbit. J. Neurosci. *26*, 609–621.

Luzzati, F., De Marchis, S., Parlato, R., Gribaudo, S., Schütz, G., Fasolo, A., and Peretto, P. (2011a). New striatal neurons in a mouse model of progressive striatal degeneration are generated in both the subventricular zone and the striatal parenchyma. PLoS One *6*, e25088.

Luzzati, F., Fasolo, A., and Peretto, P. (2011b). Combining Confocal Laser Scanning Microscopy with Serial Section Reconstruction in the Study of Adult Neurogenesis. Front. Neurosci. *5*, 70.

Luzzati, F., Nato, G., Oboti, L., Vigna, E., Rolando, C., Armentano, M., Bonfanti, L., Fasolo, A., and Peretto, P. (2014). Quiescent neuronal progenitors are activated in the juvenile guinea pig lateral striatum and give rise to transient neurons. Development *141*, 4065–4075.

Magnusson, J.P., Göritz, C., Tatarishvili, J., Dias, D.O., Smith, E.M.K., Lindvall, O., Kokaia, Z., and Frisén, J. (2014). A latent neurogenic program in astrocytes regulated by Notch signaling in the mouse. Science *346*, 237–241.

Magnusson, J.P., Zamboni, M., Santopolo, G., Mold, J.E., Barrientos-Somarribas, M., Talavera-López, C., Andersson, B., and Frisén, J. (2020). Activation of a neural stem cell transcriptional program in parenchymal astrocytes. Elife *9*, e59733.

Mori, T., Tanaka, K., Buffo, A., Wurst, W., Kühn, R., and Götz, M. (2006). Inducible gene deletion in astroglia and radial glia–a valuable tool for functional and lineage analysis. Glia *54*, 21–34.

Nagai, J., Rajbhandari, A.K., Gangwani, M.R., Hachisuka, A., Coppola, G., Masmanidis, S.C., Fanselow, M.S., and Khakh, B.S. (2019). Hyperactivity with Disrupted Attention by Activation of an Astrocyte Synaptogenic Cue. Cell *177*, 1280–1292.e20.

Nato, G., Caramello, A., Trova, S., Avataneo, V., Rolando, C., Taylor, V., Buffo, A., Peretto, P., and Luzzati, F. (2015). Striatal astrocytes produce neuroblasts in an excitotoxic model of Huntington's disease. Development *142*, 840–845.

Obernier, K., and Alvarez-Buylla, A. (2019). Neural stem cells: Origin, heterogeneity and regulation in the adult mammalian brain. Development (Camb.) *146*, dev156059. https://doi.org/10.1242/dev.156059.

Obernier, K., Cebrian-silla, A., Thomson, M., Rodriguez, R., Alvarez-buylla, A., and Alvarez-buylla, A. (2018). Adult Neurogenesis Is Sustained by Symmetric Self- Renewal and Differentiation Article Adult Neurogenesis Is Sustained by Symmetric Self-Renewal and Differentiation. Cell Stem Cell *22*, 221–234.e8.

O'Shea, T.M., Ao, Y., Wang, S., Ren, Y., Cheng, A.L., Kawaguchi, R., Shi, Z., Swarup, V., and Sofroniew, M.V. (2024). Derivation and transcriptional reprogramming of border-forming wound repair astrocytes after spinal cord injury or stroke in mice. Nat. Neurosci. *27*, 1505–1521. https://doi.org/10.1038/s41593-024-01684-6.

Parras, C.M., Galli, R., Britz, O., Soares, S., Galichet, C., Battiste, J., Johnson, J.E., Nakafuku, M., Vescovi, A., and Guillemot, F. (2004). Mash1 specifies neurons and oligodendrocytes in the postnatal brain. EMBO J. *23*, 4495–4505.

Pilz, G.-A., Bottes, S., Betizeau, M., Jörg, D.J., Carta, S., April, S., Simons, B.D., Helmchen, F., and Jessberger, S. (2018). Live imaging of neurogenesis in the adult mouse hippocampus. Science *359*, 658–662.

Ponti, G., Obernier, K., Guinto, C., Jose, L., Bonfanti, L., and Alvarez-Buylla, A. (2013). Cell cycle and lineage progression of neural progenitors in the ventricular-subventricular zones of adult mice. Proc. Natl. Acad. Sci. USA *110*, E1045–E1054.

Porcheri, C., Suter, U., and Jessberger, S. (2014). Dissecting integrin-dependent regulation of neural stem cell proliferation in the adult brain. J. Neurosci. *34*, 5222–5232.

Robel, S., Mori, T., Zoubaa, S., Schlegel, J., Sirko, S., Faissner, A., Goebbels, S., Dimou, L., and Götz, M. (2009). Conditional deletion of beta1-integrin in astroglia causes partial reactive gliosis. Glia *57*, 1630–1647.

Rolando, C., Parolisi, R., Boda, E., Schwab, M.E., Rossi, F., and Buffo, A. (2012). Distinct roles of Nogo-a and Nogo receptor 1 in the homeostatic regulation of adult neural stem cell function and neuroblast migration. J. Neurosci. *32*, 17788–17799.

Singh, S.K., Kordula, T., and Spiegel, S. (2022). Neuronal contact upregulates astrocytic sphingosine-1-phosphate receptor 1 to coordinate astrocyte-neuron cross communication. Glia *70*, 712–727.

Sirko, S., Behrendt, G., Johansson, P.A., Tripathi, P., Costa, M., Bek, S., Heinrich, C., Tiedt, S., Colak, D., Dichgans, M., et al. (2013).

Reactive glia in the injured brain acquire stem cell properties in response to sonic hedgehog glia. Cell Stem Cell *12*, 426–439.

Sirko, S., Schichor, C., Della Vecchia, P., Metzger, F., Sonsalla, G., Simon, T., Bürkle, M., Kalpazidou, S., Ninkovic, J., Masserdotti, G., et al. (2023). Injury-specific factors in the cerebrospinal fluid regulate astrocyte plasticity in the human brain. Nat. Med. *29*, 3149–3161.

Snippert, H.J., van der Flier, L.G., Sato, T., van Es, J.H., van den Born, M., Kroon-Veenboer, C., Barker, N., Klein, A.M., van Rheenen, J., Simons, B.D., and Clevers, H. (2010). Intestinal crypt homeostasis results from neutral competition between symmetrically dividing Lgr5 stem cells. Cell *143*, 134–144.

Song, J., Zhong, C., Bonaguidi, M.A., Sun, G.J., Hsu, D., Gu, Y., Meletis, K., Huang, Z.J., Ge, S., Enikolopov, G., et al. (2012). Neuronal circuitry mechanism regulating adult quiescent neural stem-cell fate decision. Nature *489*, 150–154.

Srinivas, S., Watanabe, T., Lin, C.S., William, C.M., Tanabe, Y., Jessell, T.M., and Costantini, F. (2001). Cre reporter strains produced by targeted insertion of EYFP and ECFP into the ROSA26 locus. BMC Dev. Biol. *1*, 4.

Sun, W., Cornwell, A., Li, J., Peng, S., Osorio, M.J., Aalling, N., Wang, S., Benraiss, A., Lou, N., Goldman, S.A., and Nedergaard, M. (2017). SOX9 Is an Astrocyte-Specific Nuclear Marker in the Adult Brain Outside the Neurogenic Regions. J. Neurosci. *37*, 4493–4507.

Than-Trong, E., Kiani, B., Dray, N., Ortica, S., Simons, B., Rulands, S., Alunni, A., and Bally-Cuif, L. (2020). Lineage hierarchies and stochasticity ensure the long-term maintenance of adult neural stem cells. Sci. Adv. *6*, eaaz5424.

Thored, P., Arvidsson, A., Cacci, E., Ahlenius, H., Kallur, T., Darsalia, V., Ekdahl, C.T., Kokaia, Z., and Lindvall, O. (2006). Persistent production of neurons from adult brain stem cells during recovery after stroke. Stem Cell. *24*, 739–747.

Urbán, N., Blomfield, I.M., and Guillemot, F. (2019). Quiescence of Adult Mammalian Neural Stem Cells: A Highly Regulated Rest. Neuron *104*, 834–848. https://doi.org/10.1016/j.neuron.2019.09.026.

Zamboni, M., Llorens-Bobadilla, E., Magnusson, J.P., and Frisén, J. (2020). A Widespread Neurogenic Potential of Neocortical Astrocytes Is Induced by Injury. Cell Stem Cell *27*, 605–617.e5.

Stem Cell Reports, Volume *19*

Supplemental Information

Dynamic spatiotemporal activation of a pervasive neurogenic competence in striatal astrocytes supports continuous neurogenesis following injury

Marco Fogli, Giulia Nato, Philip Greulich, Jacopo Pinto, Marta Ribodino, Gregorio Valsania, Paolo Peretto, Annalisa Buffo, and Federico Luzzati

Supplemental figures

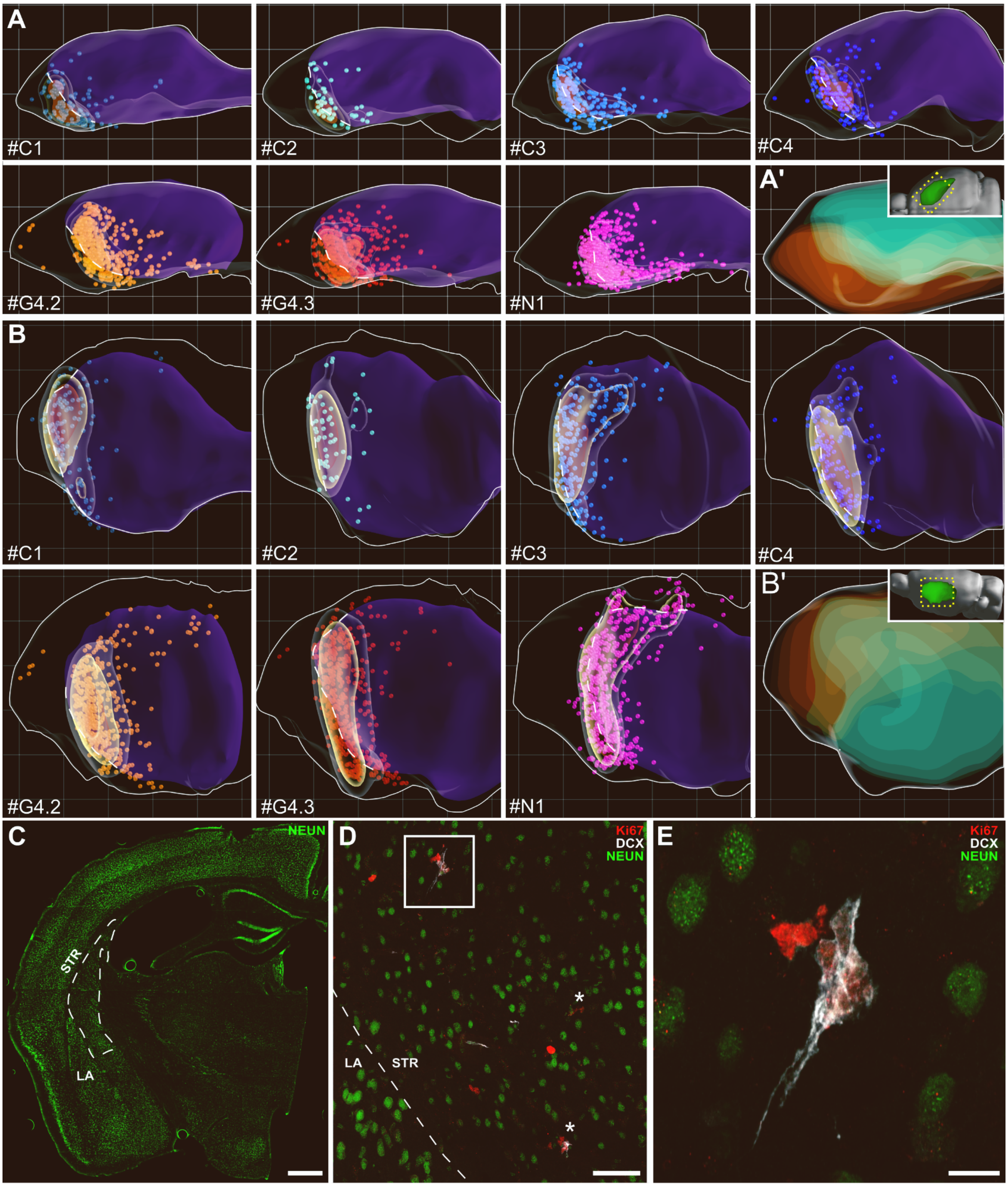

Figure S1. Neurogenic foci distribution in individual specimens, related to Figure 1

(A) Dorsal and (B) Medial views of the 3D reconstructions of the KCs over the entire STR of 7 specimens registered to a common reference space (see also Figures 1D-1E and Video S1). The lesion is in purple. A white dashed line shows the lesion

border in the neurogenic area. Dots: KCs (25 µm diameter) colour-coded as in Figures 1D-1I. Increasing KC density is rendered as transparent, yellow and orange volumes. (A’) Dorsal and (B’) medial view of a reference STR with the associative functional domain of the striatum, receiving projections from the anterior cingulate cortex, in orange and the somato-motor domain, receiving afferents from the somatosensory cortex, in cyan. It highlights the medial and lateral striatum, respectively (3D reconstructed from Hintiran et al. 2016, see Method details). Grid lines are 500 µm spaced. (C-E’) In a few specimens displaying a strong neurogenic response and partially intact caudal STR, rare KCs were found also at this level, corresponding to the caudal multimodal STR domain. These few KCs were observed on the posterior margin of the lesion border, where spared neurons were present. (C) Section labeled for neuronal marker NEUN (green) at the level of the caudal STR (dashed-line). (D) Higher magnification of the same section showing also Ki67 (red) and DCX (white) staining, highlighting the presence of KCs (asterisks). LA: Lateral Amygdala. (E) Higher magnification of the KC in the rectangle in (D). Scale: (C) 500 µm; (D) 50 µm; (E’) 10 µm.

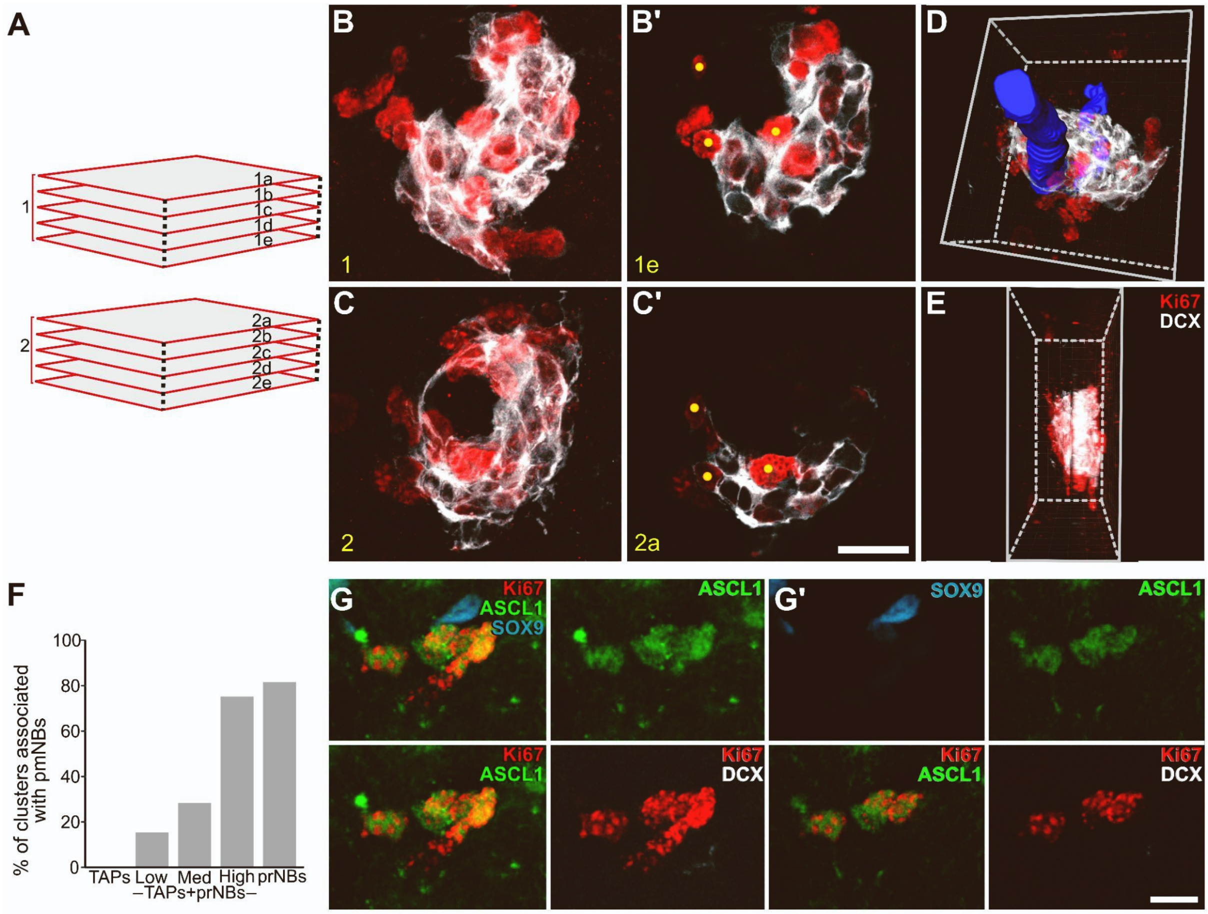

Figure S2. Method of high resolution 3D reconstruction and cellular composition of KCs, related to Figure 2

(A) Schematic view of the focal planes of two confocal stacks acquired from two consecutive sections. (B,C) Maximal intensity projection of sections 1 and 2 showing a KC splitted between the two sections. (B',C') The last intact focal plane (the section surface is usually not perfectly flat) of section 1 and the first one of section 2, respectively. Yellow dots highlight corresponding Ki67^{+} cells in the two sections. (D) Imaris 3D rendering of the reconstructed KC. A blood vessel was manually segmented and shown in blue. (E) Side view of the reconstructed KC. (F) Percentage of KCs associated with pmNBs subdivided by KC types. Note that TAPs-only KC were never associated with pmNBs, while the probability of this association progressively rose from TAPs+prNBs_Low to prNBs-only (Table S1, *p<0.001*). (G,G') TAPs-only KC stained for ASCL1 and SOX9. (G) Maximal intensity (MAX) projection; (G') Single focal plane. Scale: (B-C') 15 μm; (G,G') 5 μm.

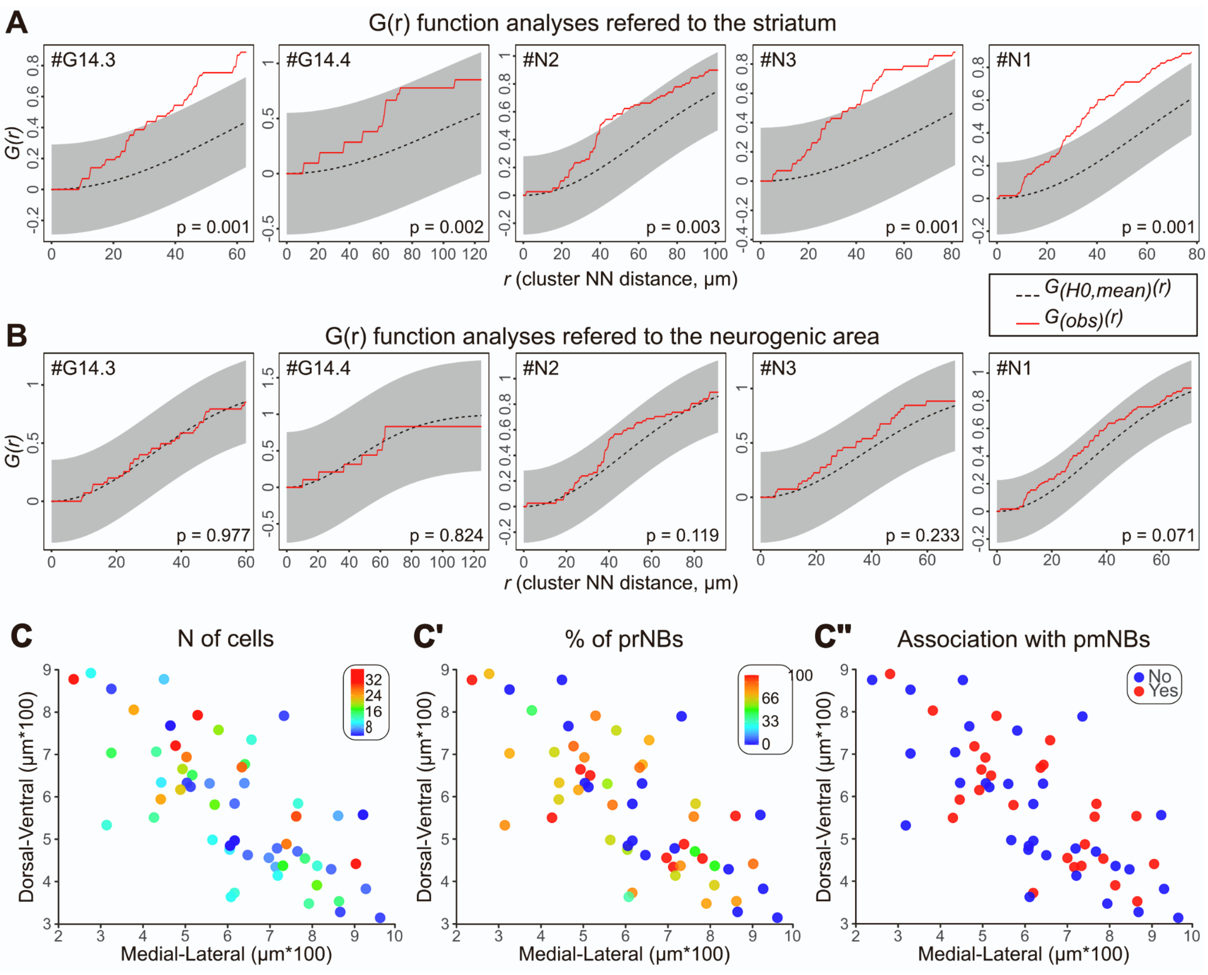

Figure S3. Spatial analysis of KC distribution, related to Figure 2

(A,B) G function analyses of KC distribution in respect of the striatum (A) or the neurogenic area (B) in five specimens. Red lines: $G_{(obs)}(r)$, functions calculated from the experimental data. Black dashed lines: $G_{(H0,mean)}(r)$, mean functions of the simulations of complete spatial randomness (*H0* = homogeneous Poisson process). Grey areas: 95% confidence envelopes. Unlike in Figure 2G, here simultaneous - instead of pointwise - envelopes were constructed so that we could reject the null hypothesis if the $G_{(obs)}(r)$ function lay outside the envelope at any value of *r.* As an additional approach for statistical comparison, the Diggle-Cressie-Loosmore-Ford (DCLF) test was performed and the resulting p-values are reported for each specimen and area (see also Method details). (C-C'') X-Y projection of KCs shown in Figure 2F coloured by size, percentage (%) of prNBs and association with pmNBs.

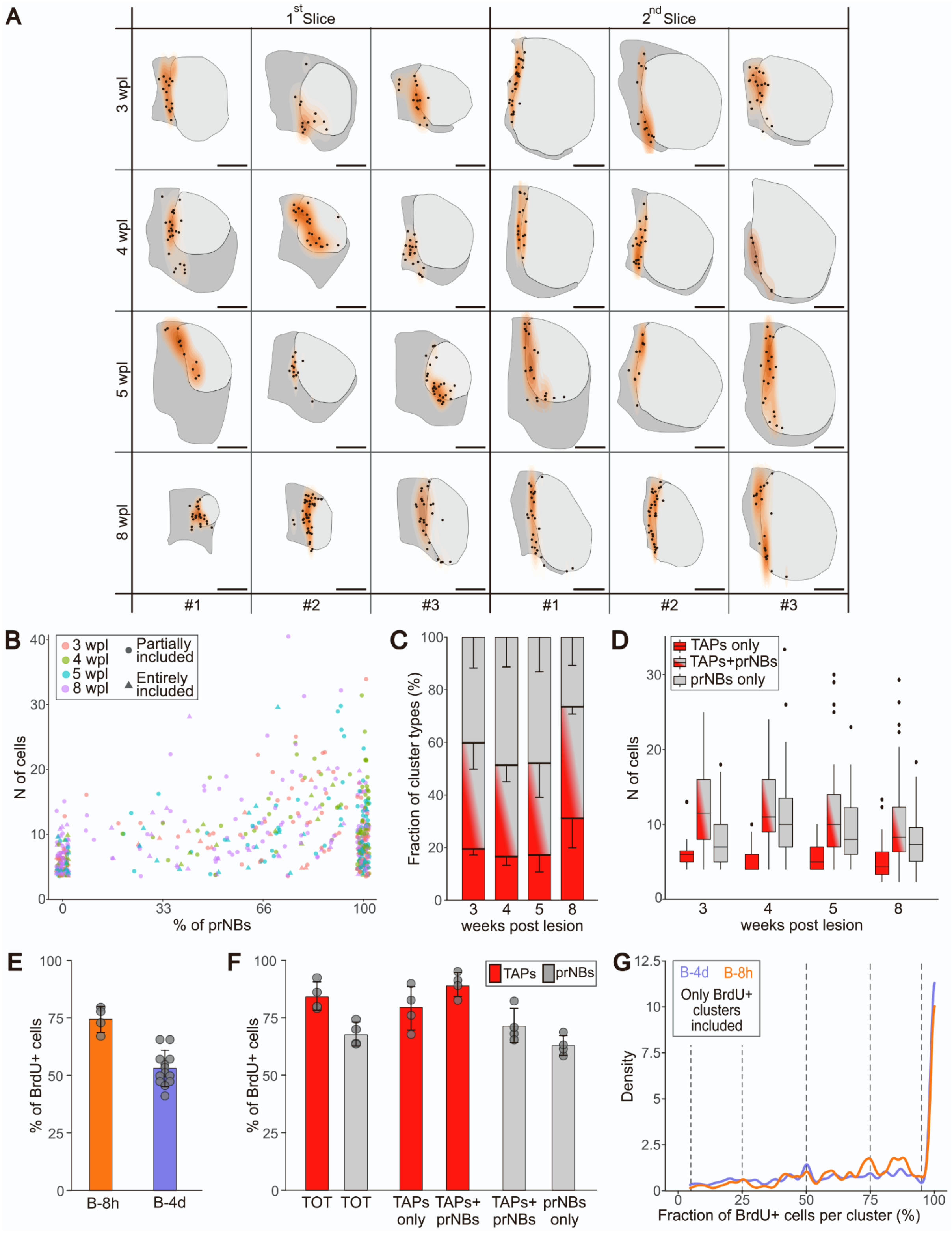

Figure S4. KC distribution and cellular composition are stable after neurogenesis onset, related to Figure 3

(A) Schematic view of the KC distribution in the two more anterior sections of the three analysed. Only three specimens (#) per group were included in this representation. Each KC is shown as a dot and the kernel density estimate of KC distribution is shown on a graded orange scale. Higher KC density corresponds to

higher orange intensity. Of note, the highest KC density is always ob-served at the border between the lesioned (light grey) and spared (darker grey) striatal tissue. (B) Percentage of prNBs vs number of cells in each KC. The colour of the dots indicates the group from which those KCs belong, while the shape indicates if the KC was partially (circle) or entirely (triangle) included in the analysed sections. (C) Relative fraction of KC types at each time point (Table S1, *TAPs-only: $p = 0.049$, TAPs+prNBs: $p = 0.873$, prNBs-only: $p = 0.213$*). Only concerning TAPs-only KCs, we found a significant difference overtime. Yet, post hoc analyses revealed a barely significant difference only when comparing 5wpl with 8wpl (Table S1, $p = 0.047$). The low number of specimens and the high variability may explain this small difference. (D) Number of cells per KC at different time points. Data were splitted into the three main KC types and shown as box and whisker plots to highlight the data distribution (Table S1, *TAPs-only: $p = 0.468$, TAPs+prNBs: $p = 0.530$, prNBs-only: $p = 0.073$*). Outliers are shown as black dots. (E) Percentage of $BrdU^+$ cells among all the KC cells in B-8h and B-4d groups. (F) Percentage of $BrdU^+$ TAPs (red) or prNBs (grey) in the whole population (TOT) or by KC type (TAPs-only, TAPs+prNBs, prNBs-only) in the B-8h group. (G) If only the $BrdU^+$ KCs are considered, the relative distribution of the % of $BrdU^+$ cells per KC is similar between the B-8h and B-4d groups (Table S1, $p = 0.247$). If BrdU dilution under detection level or cellular turnover occurred at substantial levels in KCs, we would expect a progressive leftward shifting of the orange curve. Thus, these analyses strongly support that neither BrdU dilution under detection levels, nor cellular turnover occurred within KCs. Thus, $BrdU^-$ KCs observed at each time point are newly formed structures. Data in (C), (E) and (F) are expressed as mean ± SD. Dots in (E-F) refer to individual specimens. Data in (D) are reported as box and whisker plots, where dots indicate outliers. Scale: (A) 500 µm.

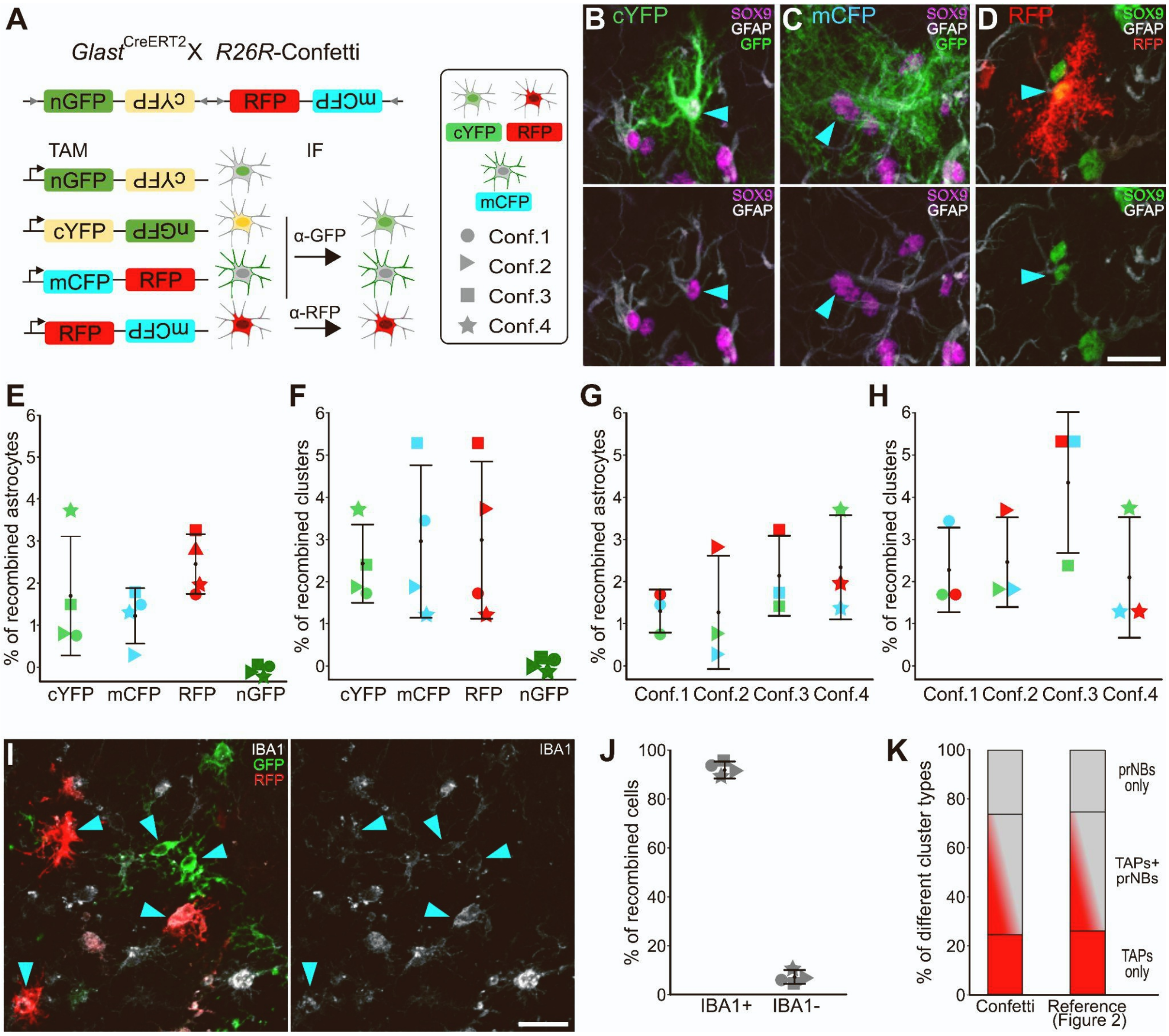

Figure S5. Confetti reporters are expressed homogeneously among striatal ASs, KCs and microglial cells of different specimens, related to Figure 4

A) Schematics of the *R26R*-Confetti locus. Tamoxifen (TAM) administration induces the stochastic and non-combinatorial expression of 1 of the 4 reporters. After immunostaining with α-GFP and α-RFP antibodies recombined cells showed either a cytoplasmic GFP staining (cYFP), a membrane-localised GFP staining (mCFP) or an RFP staining (RFP). In line with previous reports, we never observed the nGFP reporter (Calzolari et al., 2015). Each specimen is represented with a different symbol throughout the figure. (B-D) Representative images of recombined SOX9$^+$GFAP$^+$ striatal ASs. (E) Fraction of SOX9$^+$GFAP$^+$ cells (n = 1597 ASs, n = 4 mice). (F) KCs expressing the cYFP, mCFP or RFP (n = 49 KCs; n = 4 mice). (G,H) Same as (E,F) but different specimens are on the x-axis to show that they have similar recombination efficiency of striatal ASs and KCs (see also Table S1). The representations from (E) to (H) show that the fraction of striatal ASs and KCs expressing the different Confetti reporters were similar between reporter and specimens. (I) Consistent with the higher confetti recombination efficiency of confetti allele recombination in non-neural tissues (Snippert et al., 2010) we observed numerous IBA1$^+$ (grey) microglial cells, that also express *Glast*, expressed the confetti reporters (cyan arrowheads). As demonstrated by this image these cells are

easily recognized by their morphology. (J) Fraction of recombined cells (GFP^+ and RFP^+ pooled together) expressing the microglial marker IBA1. (K) Relative frequencies of KC maturation profiles in the Confetti KCs and the reference population of KCs analysed in Figure 2, (Table S1, *TAPs-only: p = 1; TAPs+prNBs: p = 0.454; prNBs-only: p = 0.301)*. Data in (E-H) and (J) are expressed as mean ± SD; dots refer to individual specimens. Scale (B-D) and (I) 20 μm.

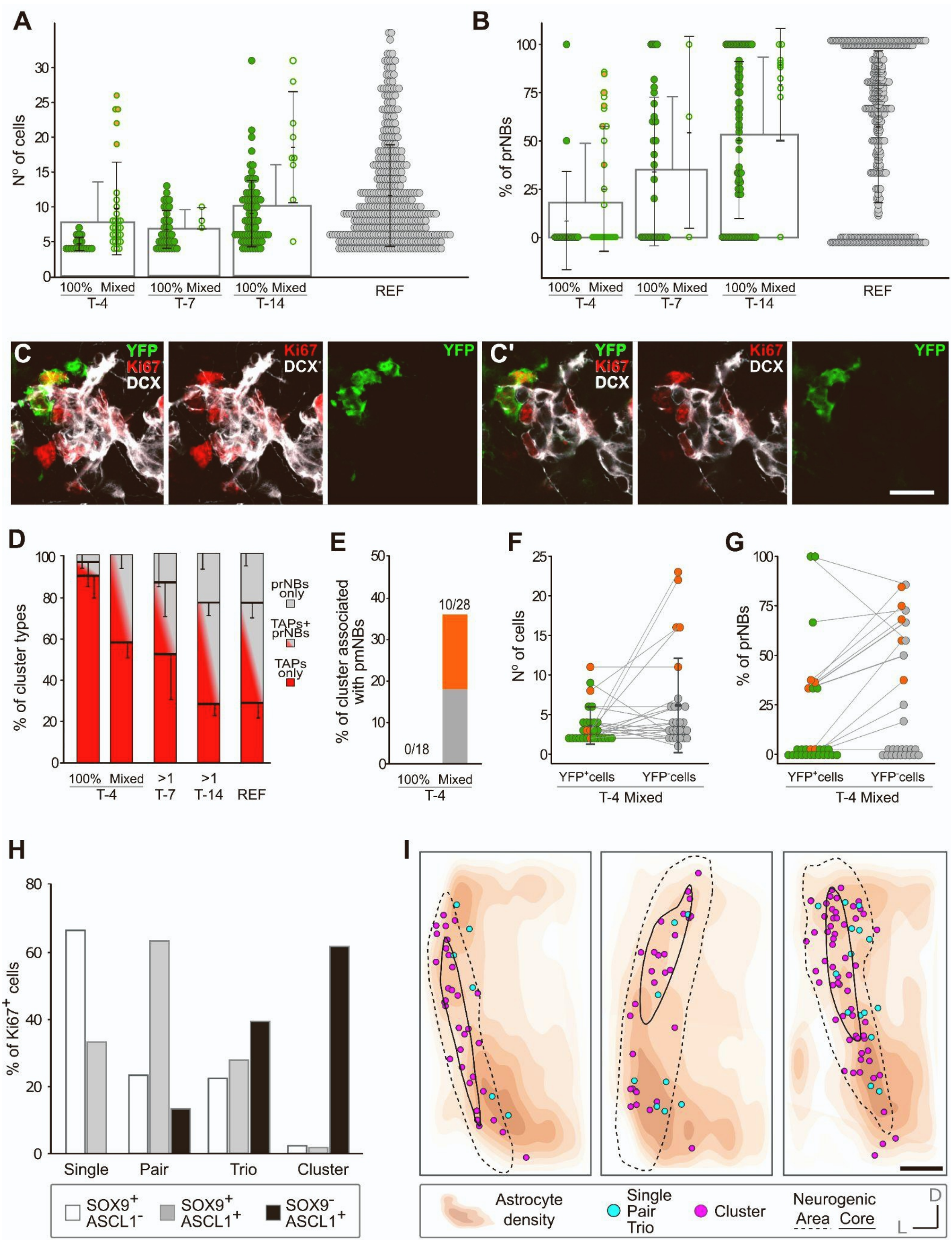

Figure S6. Striatal ASs activate only at the beginning of neurogenic foci life, related to Figures 5 and 6

(A,B) Number of Ki67+ cells and % of prNBs per KC. The data of the lineage tracing groups (T-4, T-7 and T-14) were compared with the data from the reference population of STR KCs analysed in Figure 2 (“REF”, see also Table S1). At each

time-point, data from different specimens were pooled together and shown as mean (white box with grey contour) ± SD. The data are also reported splitted between KCs entirely composed of YFP^+ cells ("100%") and those composed of a mixture of YFP^+ and YFP^- cells ("Mixed"). Like in Confetti mice, all the Mixed KCs in T-7 and T-14 likely represent KC fusion as they displayed a more advanced maturation profile than their 100% counterparts and their number correlated with the mean nearest neighbour distance among KCs, a measure of KC density (*Regression analysis with exponential decay fit: p = 0.004*). Concerning T-4 Mixed KCs, only 5 could be derived from KC fusion for the above mentioned criteria (the orange ones, as in Figures S6E-S6G; see also Figure 5I). All others displayed only slightly more advanced maturation profiles than the 100% YFP^+ ones. Further, their number did not correlate with the mean nearest neighbour distance among KCs (*Regression analysis with exponential decay fit: p = 0.957*) suggesting that fusion due to high KC density cannot explain all the T-4 mixed KCs. (C,C') Representative image of one of the five T-4 KCs that likely resulted from KC fusion. (C) MAX projection including the entire KC while (C') single focal plane in which it is easier to see which cell is YFP^+ and which one is not. (D) Relative proportion of different KC types among YFP^+ KCs. T-4 KCs were splitted into 100% YFP^+ and Mixed KCs, while in other groups all KCs with at least 1 YFP^+ cell (>1) were considered together. Data are shown as mean ± SD among specimens. The 100% T-4 KCs are the most immature ones, while the T-4 Mixed resemble the T-7 but are much more immature than T-14 and REF KCs. (E) Fraction of KCs associated with pmNBs among 100% YFP^+ and Mixed KCs of T-4. (F) Number of cells and % of prNBs (G) among the YFP^- and the YFP^+ cells of T-4 Mixed KCs. Each dot represents a KC. Of note, when the five putatively fused KCs were excluded from the analyses, we did not observe any difference in the number of cells and in the % of prNBs among the YFP^+ and YFP^- compartments of T-4 Mixed KCs (Table S1). These data indicate that both groups of cells originated almost simultaneously at the beginning of the KC life. This is in line with the hypothesis that striatal ASs activate only at the beginning of KC life and with the possibility that *Glast*^{CreERT2} recombination occurs up to the 2- or 3-cell stage. (H) Percentage of $Ki67^+$ cells organised as single, pair, trio or KCs that express only SOX9 (white), SOX9 and ASCL1 (grey) or only ASCL1 (black). (I) AS density map (increasing orange intensity) and spatial distribution of KC (magenta) and single/pair/trios (light blue) in two consecutive 3D-reconstructed slices (*n* = 3 mice). The neurogenic area and its core used to quantify cell densities in Figures 6L and 6N-SP are depicted as black lines (see also Method details). Quantifications in Figures 6K, 6L and 6N-6P were taken from these distributions, also considering the Z dimension. Scale: (C-C') 15 µm; (I) 250 µm.

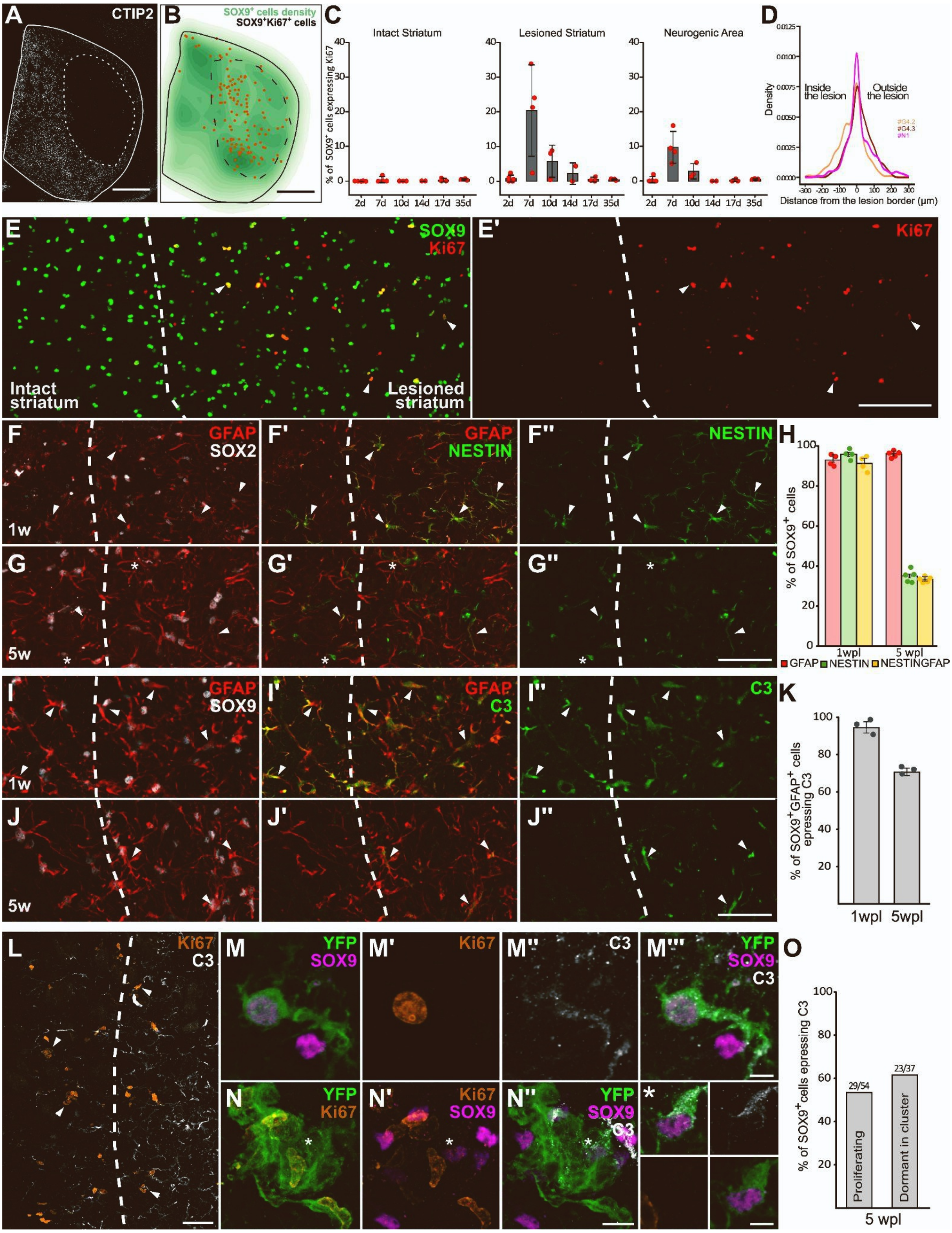

Figure S7. STR AS reactivity

(A,B) A couple of subsequent sections at the level of the rostral part of the lesion in a specimen at 1 wpl showing in B the density of SOX9^{+} cells (green) and the position of SOX9^{+}Ki67^{+} proliferating ASs (orange dots). SOX9 and Ki67^{+} cells were counted automatically after segmenting them with Ilastik. The lesion border was drawn after

aligning with the immediately adjacent rostral section (A), labeled with CTIP2 (white). Note how the lesion border is already sharp at this stage. (C) Quantification of the fraction of ASs proliferating inside and outside the lesion or in the neurogenic area, which lies in between. For this analysis cells were counted manually in a section at the level of the central part of the lesion. The neurogenic area at 1 wpl was drawn as a 200-300 μm band centered on the lesion border. (D) 3D distances of neurogenic foci relative to the lesion border in the entirely reconstructed specimens #G4.2, #G4.3 and #N1 (Figures 1 and S1; Video S1). Note that these structures are relatively homogeneously distributed around the border. (E,E') Confocal stack spanning the entire section acquired around the lesion border from the section in (B) labeled for SOX9 (green) and Ki67 (red). Double labeled cells are located mostly inside the lesion. (F-G'') Confocal stack spanning 5 μm thickness of sections labeled for GFAP (red), NESTIN (green) and the AS marker SOX2 (white) taken at 1wpl and 5wpl. Arrowheads indicate double labeled cells, NESTIN also labels non-astrocytic cells, mostly within blood vessels (asterisk). (H) Quantification of the fraction of $SOX2^+GFAP^+$, $SOX2^+NESTIN^+$ and co-labelled cells in the neurogenic areas at 1wpl and 5wpl. (I-J'') Confocal stack spanning 5 μm thickness of sections labeled for GFAP (red), C3 (green) and SOX9 (white) taken at 1wpl and 5wpl. Arrowheads indicate double labeled cells. (K) Quantification of the fraction of $C3^+SOX9^+$ cells in the neurogenic area. Although C3 was originally proposed as a marker of neurotoxic ASs (Liddelow et al. 2017), it shows a delayed upregulation also in conditions that are not expected to stimulate this state such as 7 days after stroke (Zamanian et al. 2012) or in mature border ASs after spinal cord injury and it is physiologically expressed in the glia limitans (O'Shea et al. 2024). (L) Low magnification of the neurogenic area labeled for C3 and Ki67, arrowheads indicate KCs. (M) Proliferating AS in the neurogenic area labeled with Ki67, SOX9, and YFP in a *Glast*$^{CreERT2/+}$x*R26R*-YFP mice 14 days after tamoxifen injection. (N) Recombined KC containing a dormant $YFP^+SOX9^+Ki67^-$ AS (asterisk). (O) Fraction of C3 expression among $SOX9^+$ cells also expressing $Ki67^+$ in the neurogenic area (proliferating ASs) or $YFP^+SOX9^+Ki67^-$ directly associated with a YFP^+ KC (Dormant in Cluster). Data in ©, (H) and (K) are expressed as mean ± SD; dots indicate individual specimens. Scale: (A,B) 500 μm; (E,E') 150 μm; (F-G'') 50 μm; (I-J'') 50 μm; (L) 50 μm; (M-M'''') 5 μm; (N-N'') 10 μm; (*) 5 μm.

Supplemental experimental procedures

Histology

Animals were anesthetised with a ketamine (100 mg/kg) and xylazine (33 mg/kg) solution and perfused with a solution of 4% paraformaldehyde (PFA, Sigma-Aldrich) and 2% picric acid (AnalytiCals) in 0.1 M sodium phosphate buffer (PB) pH 7.4. Brains were then post-fixed for 5 hours, cryoprotected in 30% sucrose (Sigma-Aldrich) in 0.1M PB pH 7.4, embedded at −80°C in Killik/OCT (Bio-Optica), and cryostat sectioned in series of 50 µm-thick coronal sections.

3D reconstructions

Confocal microscopy serial section 3D reconstructions were performed by modifying a previous method (Luzzati et al., 2011). All the following image processing and alignment procedures were performed with Fiji using a set of custom-made scripts available at the following link: https://github.com/bunbunet/FogliNato2024_3Drec. Whole-section epifluorescence acquisitions (Zeiss Axio scan) or cryotomography images served as a backbone anatomical reference for the 3D reconstructions of high resolution confocal acquisitions.

Low resolution whole sections registration

Epifluorescence images were extracted from the original Carl Zeiss Image (.czi) file format using the script *"Export tif from czi.ijm"*. This script allows saving as .tif files the images of each section splitted by channel, assigning a prefix with the channel name and the desired LUTs. Additionally, it includes a "zN" tag codifying the position of each section relative to the entire brain, allowing a straightforward image alignment (see below).

Epifluorescence images were then imported in the Fiji plugin TrakEM2 (Cardona et al., 2012) using the script *"Import patches z names.py"* which imports the images into the appropriate layer based on their "zN" tag. The script also scales each image based on its pixel size to reach a final resolution of 1 µm/pixel. Thus, all the images that are imported with this script can be easily overlaid even though they were acquired at different resolutions.

Sequential sections were manually aligned with a landmark-based registration method to obtain a registration of all the sections that will work as the anatomical reference of the 3D reconstruction; we only used rigid transformations, i.e. translation and rotation.

To ease this initial alignment phase, in some specimens, we performed cryotomography imaging during the cryostat cutting procedure. Briefly, images were acquired with a customised system composed of a Raspberry Pi camera (V2 8MP) with additional 16mm M12 objective mounted through a customised support on the cryostat anti-roll glass, which was controlled through the script *"CRIOpi Capture.py"*. Images were then calibrated and processed using the script *"CRIOpi Processing.ijm"*

and imported as the first images in the TrakEM2 project using the same importing script. In those cases, epifluorescence images were simply overlaid with the cryotomography images and minimal adjustments to the overall image alignment were needed.

Confocal high-resolution images

The multi-channel z-stacks confocal acquisitions of the striatum (“z-stacks”) were named including the same “zN” tags of corresponding epifluorescence/cryotomography images. The z-stacks were initially batch-converted from the Leica image file (.lif) format into .tif files with the script *“Export tif from lif.ijm”*. Then, the z-stacks of each specimen were processed separately using the script *“Split tif to MAX and Sequence.ijm”* to obtain: i) the maximal intensity projections (“MAX projections”) of each channel and section; ii) the sequence of the single focal planes for each channel and section (“sequences”); iii) a .csv file containing the information for importing the sequences (see below). Similarly to what is done with the epifluorescence images, the script allows to apply the desired LUT to each channel and adds a prefix with the marker name (e.g. “Ki67^{-}”).
Then, the MAX projections of each confocal section were imported using the same importing script and manually overlaid to the corresponding epifluorescence images. Finally, the sequences of each section were imported at the right positions with the “import from text file” TrakEM2 function, using the .csv file previously generated by the script “*Split tif to MAX and Sequence.ijm*”. As a last step, the same transformation previously applied on each MAX projection was applied to its corresponding sequences to register all the z-stacks.
For each layer in TrakEM2 are now available separate image files for each channel that could be visualised as a merged image using the script *“Set patch composite mode.py”*. For inspection and manual counting, the script *“Set visibility toggle channels.py"* enables the use of customizable keyboard shortcuts for changing the visibility of each channel.

Annotations, region drawing and calibration

The KCs were manually annotated as TrakEM2 “ball objects”. The areas of the dorsal striatum were manually drawn according to the Allen Brain Atlas subdivisions. The lesioned areas were manually drawn based on GFAP expression. To correct the section shrinking along the z-axis and adjust the voxel size accordingly, the z-spacing of each focal plane was calculated by dividing the total depth of the reconstructed volume, according to cryostat sectioning, by the total number of focal planes. Of note, this is a fundamental requirement for obtaining proper distance measurements and performing 3D spatial analyses.

3D model alignment and visualisation in Blender

Surface areas drawn in TrakEM2 of the striatum, lesioned area and lateral ventricle, were exported as .obj files from TrakEM2 using the script *“Export Arealists.py”* and

imported in Blender (v2.79) with the script “*Blender_Import Surfaces and Reference points.py*”. KC coordinates were exported using the script *“Export Balls.py”* or *“Export Balls Confetti.py”* and imported in Blender as spheres using the script “*Blender_Import_Coordinates_as_Balls.py*”. Each specimen was imported in a different layer, after assigning materials, renderings were performed exclusively as orthographic views. All surfaces have been smoothed with a *smooth* modifier and additional imperfections were manually smoothed in sculpt mode. Reconstructions of the striatal projection domains of the anterior cingulate cortex (ACA) and the primary somatosensory areas(SSp) were obtained by aligning and manually drawing in TrakEM2 the segmentations of anterograde tracers injections obtained from (http://www.mouseconnectome.org/CorticalMap/page/map/5)(Hintiryan et al., 2016) and overlaid to the Allen reference atlas CCF V3. Reference samples were the SW110323-03 and SW110321-04 for ACA and SW120525-02, SW110418-01, SW110516-02, SW110418-02, SW110419-02 for SSp. Surfaces were imported in Blender as described. The registration of the different specimens to a common reference space has been performed manually based on the overall 3D shape of the striatum.

Data visualisation

All the graphs were generated with ggplot2 (Wickham, 2016), except for the G function analyses (Figures 2G and S3A-B) whose plots were produced with a dedicated function of the spatstat package (Baddeley et al., 2015).
The relative distributions reported in Figures 1F-1I, 6C, S4G and S7D actually represent the kernel density estimates of such distributions: the area under each curve is 1 and represents 100% of the observations. Although the term “relative” is not completely correct from a mathematical point of view, it accurately describes what is shown in the graph in a more understandable manner.
The volume rendering of the KC densities reported in Figures 1C-1E, S1A and S1B were obtained by computing 3D kernel density estimates of the KC in each specimen, by using the R package ks (Duong, 2007). Increasing KC density is shown with three objects of different colours: transparent (white boundary), yellow and orange. The obtained 3D objects were visualised and exported as .obj files with the R package rgl (Murdoch and Adler, 2023).

STATISTICAL ANALYSIS

Cellular composition of KCs at 5wpl

The cellular composition of KC at 5 wpl (Figure 2) was analyzed on entirely reconstructed KCs (*n* = 430) from 3-5 consecutive 3D reconstructed sections per mouse (*n* = 8 mice). KCed cells were manually counted using the MultiPoint tool in Fiji. As previously described (Nato et al., 2015), KCs were defined as groups of at least 4 $Ki67^+$ cells showing direct contact among their cell bodies. Of note, due to this

stringent criterion used to define clustered cells, when prNBs were sparse in KCs of pmNBs not showing direct contact among their cell bodies that KC was excluded from the analysis. As a consequence, our analyses did not include the oldest KC population in which the proliferative potential is fading off (see also Supplementary text 1, section 4.2).

Time course analyses of KC distribution, number, cellular composition and turnover

Time course analyses of KC (Figures 3 and S4) were performed on three non-consecutive 400 μm-spaced sections for each specimen. It is to be noted that 63±9% of KCs were partially included in the analysed sections (Figure S4B). However, the fraction of incomplete KCs was constant among specimens (*One-way ANOVA,* $F_{(3,9)} = 1.53$, $p = 0.271$).

Clonal analyses of striatal astrocyte progeny

To visualize different reporters, we used anti-GFP antibody to recognize GFP, YFP, and CFP and anti-RFP antibody for RFP. GFP^+ cells were discriminated according to the localization of the green staining: nuclear (corresponding to “nGFP”), cytoplasmic (corresponding to “cYFP”), or membrane-associated (corresponding to “CFP”).
For each Confetti specimen (Figures 4 and S5), all the consecutive sections containing striatal newborn cells were 3D reconstructed as described in the 3D reconstruction section (#C1: *n* = 26 slices; #C2: *n* = 24 slices; #C3: *n* = 32 slices; #C4: *n* = 29 slices). The spatial localization and the type of each KC were manually annotated in TrakEM2, allowing to map the KCs containing at least 1 Confetti $Reporter^+$ cell in 3D space. KC coordinates were exported from TrakEM2 using the script *“EXPORT_Balls_3.3.py”* for further spatial statistical analyses.
The cellular composition and reporter expression of these Confetti KCs was manually evaluated on 3D reconstructions from high resolution confocal acquisitions (see the “Confocal imaging” section). The same stringent criterion used to define clustered cells was applied for considering dormant astrocytes as part of the KC - and thus clonally related to the other cells it gave rise to. The same criteria were used for the quantification of clonally-related dormant astrocytes in subsequent fate-mapping analyses with $\textit{Glast}^{CreERT2/+}$x*R26R*-YFP mice.
Striatal astrocytes recombination efficiency was measured as the fraction of $GFAP^+SOX9^+$ cells expressing each Confetti reporter (Figures 4 and S5), in four fields of view for each specimen.

Genetic fate-mapping analysis of striatal astrocytes activation and KC maturation

Fractions of YFP^+ STR KCs were counted over the entire thickness of 3 consecutive 3D reconstructed sections (Figure 5). The cellular composition of KC containing at least 1 YFP^+ cell was evaluated only for KCs entirely included in the volume (Figures

5 and 6). For the T-4 animals and two of the four T-7 animals that add less than 9 KCs within the first three sections, three or four additional slices were analysed.

Expression of ASCL1 and SOX9 in striatal Ki67$^+$ cells

Ki67$^+$ cells organized as single cells, pairs, trios or KCs were analysed on two consecutive reconstructed slices for each mouse (*n* = 3; Figures 6 and S6). We distinguished between SOX9^{High} cells expressing this transcription factor at a similar level as the non-proliferating parenchymal astrocytes and SOX9^{Low} cells expressing it at barely detectable levels (Figures 6F-6I).

Estimate of astrocyte activation rate

To estimate striatal astrocyte activation rate within the neurogenic area we segmented the SOX9$^+$ nuclei using Ilastik (Berg et al., 2019). Briefly, we trained a pixel classification model recognizing the nuclei centres, subsequently applied a watershed separation of individual objects using hysteresis thresholding and further trained an object classification model to recognise nuclei that were not separated, almost only couples of nuclei, or that were splitted, never more than in two pieces. The total number of nuclei was thus obtained by adding the number of individual nuclei, unsplitted nuclei*2 and splitted objects/2. The number of astrocytes and active Ki67$^+$ structures were counted over the neurogenic area and its core, using the R packages spatstat (Baddeley et al., 2015) and RImageJROI (https://github.com/davidcsterratt/RImageJROI). The neurogenic area and core were obtained by computing 3D kernel density estimates of the KC in each specimen with the R package ks (Duong, 2007), and included respectively the 95% and 25% of KCs with increasing density (see also Figure S6I).
Due to the extremely high density of SOX9$^+$ cells in the SVZ, quantifications in this region were performed manually, by counting the SOX9$^+$Ki67$^-$, the SOX9$^+$Ki67$^+$ and the SOX9$^-$Ki67$^+$ cells in the lateral wall of 3 SVZ from healthy mice. All counted objects, in both striatum and SVZ were counted stereologically by excluding those that touched one of the section surfaces.

Analysis of astrocyte reactivity

Astrocyte proliferation was assessed by quantifying the percentage of SOX9$^+$ cells, included in a focal plane, that expressed the endogenous marker of proliferation Ki67 (2dpl: *n* = 5; 7dpl: *n* = 4; 14dpl: *n* = 2; 17dpl: *n* = 3; 35dpl *n* = 3). The border of the lesion was drawn by registering the sections with the immediately adjacent rostral section labeled with CTIP2. For 7dpl specimens, an entire series of sections was automatically segmented for SOX9 and Ki67 as described in the previous section. One representative section of this analysis is shown in Figure S7B. The results were very similar to those obtained by the manual count revealing a rather constant level of astrocytes proliferation along the rostro-caudal axis of the lesion (data not shown). The number of GFAP and C3 labeled cells was counted on one section per

specimen (n=3) in the neurogenic area. For 1 wpl specimens, where no KCs are present, the counted area spanned a 2-300 μm band of tissue centered on the lesion border. Proliferating KCs and dormant astrocytes associated with YFP^{+} KCs have been counted on 8 50 μm-thick sections from 4 *Glast*$^{CreERT2/+}$x*R26R*-YFP mice 14 days after tamoxifen injection.

Statistical tests

The results of all the analyses are reported in Table S1. We preferred to not overload the figures with the statistics to allow a better visualization of the data. We reported in the main text only the p-values of the comparisons we specifically cited there.
All analyses were performed using R Statistical Software (R Core Team 2021).

Means comparisons

To select the proper statistical test we always checked if the data were normally distributed, using the Shapiro–Wilk test, and homoscedastic, using Levene's test. Data that fulfilled those requirements were compared with the Test-t in the case of two groups or with the ANOVA in the case of three or more groups. ANOVA analyses that returned significant F values were followed by Tukey's post hoc test. In the case of non-normally distributed and/or non-homoscedastic data, we used the nonparametric alternatives: the Mann–Whitney U test and the Kruskal-Wallis test to compare two, or more than two groups, respectively. Significant Kruskal-Wallis tests were followed by Dunn's test for multiple comparisons (p-values were adjusted according to the Benjamini-Hochberg method). In the case of paired samples, the Wilcoxon signed-rank test was used instead of the Mann-Whitney U test.

Comparison of proportions

Fisher's Exact Test was used to compare proportions among count data with binary outcomes (e.g. fraction of KC associated or not associated with pmNBs). We use this test instead of the more conventional Chi-square test as it is more suitable to compare groups with low numbers of observations. For comparisons among more than two groups, significant Fisher's Exact Tests were followed by Pairwise Fisher's Exact Tests in which p-values were adjusted according to the Holm-Sidak method.

Comparison of frequency distributions

The Kolmogorov-Smirnov test was used to compare two distributions, while the Anderson-Darling k-sample test to compare three or more distributions.

Spatial statistics (point pattern analysis)

Evaluation of KC distribution

To assess if KCs distribute according to specific spatial patterns we used the G(*r*) function (Baddeley et al., 2015). This summary function represents the cumulative

frequency distribution of the first-order nearest neighbor distances (*r*) and it is one of the simplest techniques to find clustering or dispersion in a point pattern. Although a 3D version of this function is implemented in spatstat, we preferred to use its 2D counterpart as it allows, unlike the 3D version, the use of irregular polygonal areas describing the region from which the point pattern was retrieved. As shown in Figures 1 and S1, the striata and neurogenic areas are highly irregular and cannot be approximated to a parallelepiped. Further, since the analyses were performed on a few consecutive sections, KC dispersion was much greater on the X and Y axes than in Z, suggesting the reliability of a 2D projection analysis.
The G function calculated from the data ($G_{(obs)}(r)$) is visually inspected and compared to the theoretical one obtained from a homogeneous Poisson process ($G_{(theo,H0)}$) that represents the null hypothesis (*H0*) of complete spatial randomness (CSR). If $G_{(obs)}(r)$ lies at the left or at the right of $G_{(theo,H0)}(r)$ it suggests clustering or dispersion, respectively. Yet, to avoid false-positive results, each $G_{(obs)}(r)$ was compared with 999 Monte Carlo simulations of $G_{(theo,H0)}(r)$, and the graphs in Figures 2G and S3A-S3B report the mean of these simulations $G_{(mean,H0)}(r)$ and the simulation envelopes. For statistical comparisons (Figures S3A-S3B), we constructed simultaneous envelopes having a constant width around $G_{(mean,H0)}(r)$. The width is defined by finding the most extreme deviation, at any value of *r*, of the 999 simulated $G_{(theo,H0)}(r)$ from $G_{(mean,H0)}(r)$. Thus, the visual interpretation of the graphs coincides with the results of Maximum Absolute Deviation Tests (Baddeley et al., 2015). We rejected the null hypothesis of CSR if $G_{(obs)}(r)$ felt outside of the envelope at any value of *r*. As a complementary strategy to compare the experimental distributions with the simulations, we used the more powerful Diggle-Cressie-Loosmore-Ford test (Baddeley et al., 2015), that, instead of the maximum absolute deviation, uses the integrated squared deviation between $G_{(obs)}(r)$ and $G_{(theo,H0)}(r)$ over the range of measured *r*. For representative purposes, in Figure 2G we constructed pointwise envelopes instead of simultaneous ones, as we thought they were simpler to visually interpret (e.g $G_{(theo,H0)}(r) > 0$ at every value of *r*). However, we did not use such envelopes for statistical purposes as they would be more suitable for testing if clustering/dispersion exists at specific values of *r*, which was not what we intended to test here (Baddeley et al., 2015).
To perform these analyses, the R packages spatstat (Baddeley et al., 2015) and RImageJROI (https://github.com/davidcsterratt/RImageJROI) were used. The pooled p-values shown in the main text were obtained with the R package poolr (Cinar and Viechtbauer, 2022) by using Fisher's method on the p-values reported in Figures S3A-S3B.

Global spatial autocorrelation

To analyse the 3D distribution of KC features (cellular composition in Figure 2 and Confetti reporter expression in Figure 4I) we used Moran's I index of global spatial autocorrelation(Moran, 1950). It's a widely used technique in spatial econometrics(Anselin and Rey) that, similarly to a correlation coefficient, provides an estimate of how closer objects are more similar than distant ones. Thus, it's a

powerful technique to search for spatial clustering of similar objects. It bears the great advantages of being a “distance-based” metric. So, unlike other summary functions such as the G function, there’s no need to specify the study area/volume from which the point pattern was retrieved. Further, no edge correction methods are needed, avoiding the possible introduction of biases by the selection of specific methods or areas. This greatly helps in assessing the distribution of objects embedded in highly irregular and variable environments like the lesioned striata.
A positive Moran’s I index suggests clustering of similar objects, while a negative one is indicative of homogeneous dispersion. Yet, the Index value depends on the number of data points and the bigger the sample the more it approaches 0, which represents the absence of spatial autocorrelation. Thus, the sole examination of the Moran’s I Index is difficult to interpret. To understand if the observed data distribution was significantly different from complete spatial randomness, we performed a permutation test based on Monte Carlo simulations. The simulations randomly permuted the KC features over the same point pattern distribution and every time the resulting Moran’s I Index was measured. Finally, a p-value was calculated based on the proportion of simulations giving a statistic that is as or more extreme than the observed data. To perform this analysis, the R package ade4 (Dray and Dufour, 2007) was used.

Key resources tableSOX

REAGENT or RESOURCE	SOURCE	IDENTIFIER
Antibodies		
Rat monoclonal anti-BrdU (clone BU1/75 (ICR1)) (1:1500)	AbD Serotec	Cat#OBT0030CX; RRID:AB_609566
Rabbit anti-Ki67 (1:1000)	Novocastra	Cat#NCL-Ki67p; RRID:AB_442102
Rabbit polyclonal anti-Ki67 (1:1500)	Abcam	Cat#AB15580; RRID:
Rabbit anti-Ki67 (1:750)	Thermo Fisher Scientific	Cat#MA5-14520; RRID:AB_10979488
Goat polyclonal anti-DCX (1:2000)	Santa Cruz Biotechnology	Cat#Sc-8066; RRID:AB_2088494
Guinea Pig polyclonal anti-DCX (1:1500)	Millipore	Cat#AB2253; RRID:AB_1586992
Goat anti-SOX9 (1:1200)	R and D Systems	Cat# AF3075; RRID:AB_2194160
Chicken polyclonal anti-GFP (1:1000)	Aves labs	Cat#GFP-1020; RRID:AB_10000240
Rabbit polyclonal anti-RFP (1:1000)	Rockland	Cat# 600-401-379, RRID:AB_2209751
Rat monoclonal anti-CTIP2 (clone 25B6) (1:750)	Abcam	Cat#ab18465, RRID:AB_2064130

Mouse monoclonal anti-NEUN (1:1000)	Chemicon	Cat#MAB377; RRID:AB_2298772
Mouse anti-GFAP (1:1000)	Millipore	Cat#MAB360; RRID:AB_11212597
Mouse monoclonal anti-ASCL1 (1:100)	BD PharMingen	Cat#556604; RRID:AB_396479
Rabbit anti-IBA1 (1:1000)	FUJIFILM Wako Shibayagi	Cat# 019-19741; RRID:AB_839504
Rabbit anti-S100β (1:10000)	Swant	Cat#37A; RRID:AB_2315305
Goat polyclonal anti-SOX10 (1:750)	Santa Cruz Biotechnology	Cat#Sc-17342; RRID:AB_2195374
Rat polyclonal anti-C3 (1:250)	Abcam	Cat# ab11862; RRID:AB_2066623
Rabbit polyclonal anti-C3 (1:250)	Dako	Cat# A006302, RRID:AB_578478
Rat monoclonal anti-SOX2 (1:1000)	Thermo Fisher Scientific	Cat# 14-9811-82; RRID:AB_11219471
Goat polyclonal anti-NESTIN (C-20) (1:1000)	Santa Cruz Biotechnology	Cat# sc-21247; RRID:AB_650014
Cy3 AffiniPure Donkey anti-Rabbit IgG (1:800)	Jackson ImmunoResearch	Cat#711-165-152; RRID:AB_2307443
Cy3 AffiniPure Donkey anti-Mouse IgG (1:800)	Jackson ImmunoResearch	Cat#715-165-151; RRID:AB_2315777

Cy3 AffiniPure Donkey anti-Goat IgG (1:800)	Jackson ImmunoResearch	Cat#705-165-147; RRID:AB_2307351
Alexa Fluor 488 AffiniPure Donkey anti-Rat IgG (1:400)	Jackson ImmunoResearch	Cat#712-545-153; RRID:AB_2340684
Alexa Fluor 488 AffiniPure Donkey anti-Chicken IgY (1:400)	Jackson ImmunoResearch	Cat#703-545-155; RRID: AB_2340375
Alexa Fluor 488 AffiniPure Donkey anti-Mouse IgG (1:8400)	Jackson ImmunoResearch	Cat#715-545-151; RRID:AB_2341099
Alexa Fluor 647 AffiniPure Donkey anti-Rabbit IgG (1:400)	Jackson ImmunoResearch	Cat#711-605-152; RRID:AB_2492288
Alexa Fluor 647 AffiniPure Donkey anti-Goat IgG (1:800)	Jackson ImmunoResearch	Cat#705-605-147; RRID:AB_2340437
DyLight405 AffiniPure Donkey anti-Guinea Pig IgG (1:400)	Jackson ImmunoResearch	Cat#706-475-148; RRID:AB_2340470
DyLight405 AffiniPure Donkey anti-Rat Pig IgG (1:400)	Jackson ImmunoResearch	Cat#712-475-153; RRID:AB_2340681
Alexa Fluor 594 AffiniPure Donkey anti-Goat IgG (1:600)	Jackson ImmunoResearch	Cat#705-585-003; RRID:AB_2340432
Horse Anti-Goat IgG, Biotinylated (1:100)	VectorLabs	Cat#BA-8000; RRID:AB_2336140
AMCA-avidinD (1:100)	VectorLabs	Cat# A-2008, RRID:AB_2336102

Fab fragment Donkey anti-Rabbit IgG (1:100)	Jackson ImmunoResearch	Cat#711-007-003; RRID:AB_2340587
Chemicals, peptides, and recombinant proteins		
Tamoxifen	Sigma-Aldrich	Cat#T5648
Corn oil	Sigma-Aldrich	Cat#C8267
5-bromo-2-deoxyuridine (BrdU)	Sigma-Aldrich	Cat#B5002
Quinolinic acid	Sigma-Aldrich	Cat#Q104
Experimental models: Organisms/strains		
Slc1a3$^{tm1(cre/ERT2)Mgoe}$ (*Glast*^{CreERT2})		MGI:5466676
Gt(ROSA)26Sor$^{tm1(EYFP)Cos}$ (*R26R*-YFP)		MGI:2449038
Gt(ROSA)26Sor$^{tm1(CAG-Brainbow2.1)Cle}$/J (*R26R*-Confetti)		MGI:5317215
C57BL/6JOlaHsd		MGI:2164189
Software and algorithms		
LAS X	Leica Microsystems	https://www.leica-microsystems.com/products/microscope-software/p/leica-las-x-ls/
Fiji	Fiji	https://fiji.sc,<br>RRID:SCR_002285

TrakEM2	(Cardona et al 2012)	https://imagej.net/plugins/trakem2/
Imaris	Bitplane	v9.7.2, http://www.imaris.oxinst.com, RRID:SCR_007370
Blender	Blender Foundation	v2.79, https://www.blender.org/download/releases/2-79/
Ilastik	(Berg et al., 2019)	v1.4.0,<br><br>https://www.ilastik.org/
The R Project for Statistical Computing	(R Core Team 2021)	http://www.r-project.org/, RRID:SCR_001905
ggplot2	(Wickham, 2016)	v3.4.4
RImageROI	https://github.com/davidcsterratt/RImageJROI	v0.1.2
spatstat	(Baddeley et al 2015)	v3.0-7
ks	(Duong 2007)	v1.14.1
rgl	(Murdoch and Adler, 2023)	v1.2.1, https://github.com/dmurdoch/rgl
ade4	(Dray and Dufour, 2007)	v1.7-22
poolr	(Cinar and Viechtbauer, 2022)	v1.1-1

Supplemental table

Table S1. *Results of all statistical analyses presented in the study*

* Indicates that a post hoc test was performed, please see below

Graph	Statistical test	Result	P Value	Post-hoc
Fig. 1F	Linear Regression	AdjR^2 = 0.8895	9.04E-4	
Fig. 2D (Total cells)	Kruskal-Wallis Test	K-W chi squared = 212.4, df = 4	8.24E-45	*
Fig. 2D (TAPs)	Kruskal-Wallis Test	K-W chi squared = 120.5, df = 3	6.12E-26	*
Fig. 2D (prNBs)	Kruskal-Wallis Test	K-W chi squared = 125.4, df = 3	5.30E-27	*
Related to Fig. 2B (N of cells vs association with pmNBs)	Logistic Regression	AdjR^2 = 0.31	5.96E-21	
Related to Fig. 2B (% of prNBs vs association with pmNBs)	Logistic Regression	AdjR^2 = 0.44	2.19E-25	
Fig. 2H	Permutation Test using Monte Carlo Simulations	Moran's I coefficients	*	
Fig. S2F	Fisher's Exact Test		4.99E-4	*
Fig. 3E	One-Way ANOVA	F(4,13) = 1.98	0.157	
Fig. 3G	Mann–Whitney Test	Mann–Whitney U = 22678	6.907E-11	
Fig. 3H	Mann–Whitney Test	Mann–Whitney U = 22711	1.955E-11	
Fig. 3I	Fisher's Exact Test		2.932E-12	
Fig. 3J	Independent samples t-test	t(6.6704) = 1.0764	0.3191	
Fig. S4C (% of TAPs-only)	One-Way ANOVA	F(3,9) = 3.883	0.049	
Fig. S4C (% of TAPs+prNBs)	One-Way ANOVA	F(3,9) = 0.23	0.873	
Fig. S4C (% of prNBs-only)	One-Way ANOVA	F(3,9) = 1.824	0.213	
Fig. S4D (N of cells in TAPs-only)	Kruskal-Wallis Test	K-W chi squared = 2.5423, df = 3	0.4677	
Fig. S4D (N of cells in TAPs+prNBs)	Kruskal-Wallis Test	K-W chi squared = 2.2104, df = 3	0.5299	
Fig. S4D (N of cells in prNBs-only)	Kruskal-Wallis Test	K-W chi squared = 6.9651, df = 3	0.07302	
Fig. S4E	Independent samples t-test	t(7.0858) = -5.9575	0.0005403	
Fig. S4F (TAPs vs prNBs)	Paired samples t-test	t(3) = 3.4728	0.04026	
Fig. S4F (TAPs in TAPs-only vs TAPs in TAPs+prNBs)	Paired samples t-test	t(3) = -2.1131	0.125	
Fig. S4F (prNBs in TAPs+prNBs vs prNBs in prNBs-only)	Paired samples t-test	t(3) = 2.0584	0.1317	
Fig. S4G	Kolmogorov-Smirnov Test	D = 0.093482	0.247	
Fig. 4F	Linear Regression	AdjR^2 = 0.88	0.042	
Fig. 4H	Linear Regression	AdjR^2 = 0.53	0.004	
Fig. 4I	Monte Carlo Permutation Test	Moran's I coefficients	*	
Fig. S5E	One-Way ANOVA	F(2,9) = 1.571	0.26	
Fig. S5F	One-Way ANOVA	F(2,9) = 0.156	0.858	
Fig. S5G	One-Way ANOVA	F(2,9) = 0.834	0.512	
Fig. S5H	One-Way ANOVA	F(2,9) = 1.834	0.219	
Fig. S5K (TAPs-only)	Fisher's Exact Test		1	
Fig. S5K (TAPs+prNBs)	Fisher's Exact Test		0.454	
Fig. S5K (prNBs-only)	Fisher's Exact Test		0.301	
Fig. 5D	One-Way ANOVA	F(3,10) = 14.550	0.00056	*
Fig. 5E (TAPs-only)	One-Way ANOVA	F(3,10) = 4.486	0.03056	*
Fig. 5E (TAPs+prNBs)	One-Way ANOVA	F(3,10) = 7.520	0.00638	*
Fig. 5E (prNBs-only)	One-Way ANOVA	F(3,10) = 17.801	0.00025	*

Fig. 5J	Fisher’s Exact Test		6.428E-05	*
Fig. 6K	Mann–Whitney Test	Mann-Whitney U = 1161	0.01312	
Fig. 6N	One-Way ANOVA	F(2,6) = 68.52	7.38E-08	*
Fig. 6O	One-Way ANOVA	F(2,6) = 2.435	0.168	
Fig. 6P	One-Way ANOVA	F(2,6) = 0.73	0.52	
Fig. S6A (Total)	Kruskal-Wallis Test	K-W chi squared = 37.595, df = 3	3.443E-08	*
Fig. S6A (100%)	Kruskal-Wallis Test	K-W chi squared = 52.062, df = 3	2.905E-11	*
Fig. S6B (Total)	Kruskal-Wallis Test	K-W chi squared = 43.687, df = 3	1.759E-09	*
Fig. S6B (100%)	Kruskal-Wallis Test	K-W chi squared = 31.305, df = 3	7.334E-07	*
Fig. S6F (including the 5 orange)	Wilcoxon signed-rank Test	V = 63.5	0.0414	
Fig. S6F (excluding the 5 orange)	Wilcoxon signed-rank Test	V = 52	0.2512	
Fig. S6G (including the 5 orange)	Wilcoxon signed-rank Test	V = 12	0.03756	
Fig. S6G (excluding the 5 orange)	Wilcoxon signed-rank Test	V = 9	0.4469	
Fig. S6H (SOX9^{+}ASCL1^{-} cells)	Fisher’s Exact Test		1.014E-11	*
Fig. S6H (SOX9^{+}ASCL1^{+} cells)	Fisher’s Exact Test		<2.2E-16	*
Fig. S6H (SOX9^{-}ASCL1^{+} cells)	Fisher’s Exact Test		4.492E-10	*
Fig. S7C (intact)	One-Way ANOVA	F(5,14) = 0.849	0.37361	
Fig. S7C (lesioned)	One-Way ANOVA	F(5,14) = 5.654	0.00466	*
Fig. S7C (neurogenic area)	One-Way ANOVA	F(5,14) = 10.1	0.00029	*
Fig. S7H (GFAP 1W vs 5W)	Independent samples t-test	t(4.49) = -1.90	0.12212	
Fig. S7H (NESTIN 1W vs 5W)	Independent samples t-test	t(6.9953) = 33.221	5.857E-09	
Fig. S7H (GFAP NESTIN 1W vs 5W)	Independent samples t-test	t(3.6308) = 29.9	1.758E-051	
Fig. S7K (C3 1W vs 5W)	Independent samples t-test	t(3.3483) = 8.7313	0.00204	

Post hoc analyses

**Figures 2D and S2F. Post hoc**	Figure 2D Dunn's Test for multiple comparisons			Figure S2F
Comparison	Total cells	TAPs	prNBs	Pairwise Fisher’s Exact Test
TAPs-only - TAPs+prNBs_Low	0.0412478	0.179955		0.0303
TAPs-only - TAPs+prNBs_Med	8.74E-13	0.064751		7.44E-09
TAPs-only - TAPs+prNBs_High	1.21E-44	9.51E-23		1.85E-38
TAPs-only - prNBs-only	7.18E-22			1.31E-41
TAPs+prNBs_Low - TAPs+prNBs_Med	0.1065108	0.034045	0.024782	0.542
TAPs+prNBs_Low - TAPs+prNBs_High	6.74E-05	1.43E-08	3.47E-12	0.000136
TAPs+prNBs_Low - prNBs-only	0.0265778		6.50E-11	1.64E-05
TAPs+prNBs_Med - TAPs+prNBs_High	7.84E-06	1.26E-10	1.72E-18	2.31E-09
TAPs+prNBs_Med - prNBs-only	0.2312437		5.97E-15	1.36E-11
TAPs+prNBs_High - prNBs-only	0.0001211		0.362679	0.542

**Figure 2H. Moran’s I analysis**	AnimalID	Moran's I Index	P value
Total cells	G14.3	-0.003	0.34
Total cells	G14.4	-0.058	0.821
Total cells	N2	-0.033	0.683
Total cells	N3	-0.005	0.422
Total cells	N1	0.013	0.388
% of prNBs	G14.3	-0.029	0.553
% of prNBs	G14.4	-0.085	0.516
% of prNBs	N2	-0.014	0.984
% of prNBs	N3	-0.019	0.727

% of prNBs	N1	0.001	0.724
Association w/ pmNB	G14.3	-0.038	0.247
Association w/ pmNB	G14.4	-0.057	0.929
Association w/ pmNB	N2	0.033	0.745
Association w/ pmNB	N3	-0.042	0.63
Association w/ pmNB	N1	0.034	0.222

**Figure S4C (TAPs-only). Post hoc**	Comparison	P value
	3wpl-4wpl	0.920
	3wpl-5wpl	0.807
	3wpl-8wpl	0.216
	4wpl-5wpl	0.996
	4wpl-8wpl	0.087
	5wpl-8wpl	0.047

**Figure 4I. Moran's I analysis**	Specimen	Moran's I Index	P value
	#C1	-0.005	0.622
	#C2	-0.025	0.649
	#C3	-0.005	0.991
	#C4	-0.002	0.519

**Figures 5D and 5E. Tukey Post hoc**	Total clusters	TAPs-only	TAPs+prNBs	prNBs-only
T-4 vs T-7	0.4186	0.9744	0.1374	0.7795
T-4 vs T-14	0.0021	0.3591	0.0092	0.0006
T-4 vs T-bQA	0.0016	0.0369	0.0098	0.0053
T-7 vs T-14	0.0150	0.5167	0.3027	0.0012
T-7 vs T-bQA	0.0096	0.0501	0.2642	0.0142
T-14 vs T-bQA	0.9383	0.3520	0.9949	0.5948

**Figure 5J. Pairwise Fisher's Exact Test**	P value
T-4 vs T-7	1
T-4 vs T-14	0.0039
T-4 vs REF	0.0033
T-7 vs T-14	0.00984
T-7 vs REF	0.00984
T-14 vs REF	1

**Figure 6N. Tukey Post hoc**	P value
N_Area vs N_Core	0.989
N_Area vs hSVZ	0.00013
N_Core vs hSVZ	0.00014

**Figures S6A-S6B. Dunn's Test or multiple comparisons**	Figure S6A (N of cells)		Figure S6B (% of prNBs)	
Comparison	Total	100%	Total	100%
T-4 vs T-7	0.9090	0.0288	0.0740	0.0611
T-4 vs T-14	0.0027	6.085E-05	7.069E-06	0.0006
T-4 vs REF	0.00001	4.558E-08	9.117E-09	1.621E-05
T-7 vs T-14	0.0055	0.0265	0.0267	0.0613
T-7 vs REF	0.0001	3.658E-05	0.0030	0.0022
T-14 vs REF	0.2486	0.0293	0.5487	0.2340

**Figure S6H. Pairwise Fisher's Exact Test**	SOX9^{+}ASCL1^{-}	SOX9^{+}ASCL1^{+}	SOX9^{-}ASCL1^{+}
Single VS Pair	0.116	0.284	0.556
Single VS Trio	0.116	1	0.237
Single VS Cluster	4.36E-07	2.8E-03	1.05E-03
Pair VS Trio	1	0.106	0.237
Pair VS Cluster	1.68E-04	1.11E-19	1.36E-06
Trio VS Cluster	6.08E-03	2.1E-04	0.237

**Figure S7C. Tukey Post hoc**	lesioned	neurogenic area
2d VS 7d	0.0049571	0.0003787
7d VS 10d	0.0856219	0.0158508
7d VS 14d	0.0511228	0.0028811
7d VS 17d	0.0122696	0.0011219
7d VS 35d	0.0122024	0.0014631
2d VS 10d	0.8896538	0.7136573
2d VS 14d	0.9998048	0.9999466
2d VS 17d	0.9999998	0.9999979
2d VS 35d	0.9999998	0.9999999
10d VS 14d	0.9884671	0.7697894
10d VS 17d	0.9095828	0.7457391
10d VS 35d	0.9086443	0.8182281
14d VS 17d	0.9996753	0.9999983
14d VS 35d	0.9996613	0.9999001
17d VS 35d	1.0000000	0.9999902

Supplemental references

Anselin, L., and Rey, S.J. Modern spatial econometrics in practice: AGuideto Geo Da, Geo DaSpace and PySAL.

Baddeley, A., Rubak, E., and Turner, R. (2015). Spatial Point Patterns: Methodology and Applications with R (CRC Press).

Berg, S., Kutra, D., Kroeger, T., Straehle, C.N., Kausler, B.X., Haubold, C., Schiegg, M., Ales, J., Beier, T., Rudy, M., et al. (2019). ilastik: interactive machine learning for (bio)image analysis. Nat. Methods *16*, 1226–1232. .

Cardona, A., Saalfeld, S., Schindelin, J., Arganda-Carreras, I., Preibisch, S., Longair, M., Tomancak, P., Hartenstein, V., and Douglas, R.J. (2012). TrakEM2 software for neural circuit reconstruction. PLoS One *7*, e38011. .

Cinar, O., and Viechtbauer, W. (2022). The poolr Package for Combining Independent and Dependent p Values. J. Stat. Softw. *101*, 1–42. .

Dray, S., and Dufour, A.-B. (2007). The ade4 Package: Implementing the Duality Diagram for Ecologists. J. Stat. Softw. *22*, 1–20. .

Duong, T. (2007). ks: Kernel Density Estimation and Kernel Discriminant Analysis for Multivariate Data in R. J. Stat. Softw. *21*, 1–16. .

Hintiryan, H., Foster, N.N., Bowman, I., Bay, M., Song, M.Y., Gou, L., Yamashita, S., Bienkowski, M.S., Zingg, B., Zhu, M., et al. (2016). The mouse cortico-striatal projectome. Nat. Neurosci. *19*, 1100–1114. .

Luzzati, F., Fasolo, A., and Peretto, P. (2011). Combining Confocal Laser Scanning Microscopy with Serial Section Reconstruction in the Study of Adult Neurogenesis. Front. Neurosci. *5*, 70. .

Moran, P.A.P. (1950). Notes on continuous stochastic phenomena. Biometrika *37*, 17–23. .

Wickham, H. ggplot2 (Springer International Publishing).
